# Society of Nematologists 1961

**DOI:** 10.2478/jofnem-2025-0049

**Published:** 2025-11-05

## Regulation, quarantine and impact on trade, a South American perspective

Acevedo, Oriana

Servicio Agrícola y Ganadero, Chile

### Abstract

International trade increases the risk of pests entering and spreading. To reduce this risk, importing countries establish phytosanitary entry requirements through their Plant Protection Organization (PPO) or other official agencies. In recent years, nematodes of the genera such as *Meloidogyne, Heterodera, Globodera*, and *Pratylenchus* have emerged as major plant pests in South America, impacting key crops. The economic implications of nematode infestations can be profound, with estimates suggesting billions of dollars in losses annually across the region. The response to these challenges involves a complex interplay of regulations designed to manage and mitigate the risks posed by harmful nematodes. Regulatory frameworks at both national and international levels govern the management of nematode-related plant health risks. The International Plant Protection Convention (IPPC) is an intergovernmental treaty established to develop, adopt, and promote the application of International Standards for Phytosanitary Measures (ISPMs), providing a baseline for how countries should approach the containment and management of plant pests, including nematodes. South American countries are contracting parties to the IPPC and have therefore established their own regulations, dictating quarantine procedures, pest management, and monitoring strategies, thus complying with the IPPC’s requirements regarding the adoption and promotion of international phytosanitary measures. Quarantine measures are essential for preventing the spread of nematodes across borders and within regions. The implementation of quarantine protocols involves maintaining strict guidelines for the inspection and treatment of agricultural products before export. These regulations often lead to trade restrictions when nematodes are detected, creating a dilemma for producers who must balance compliance with trade requirements and the economic impacts of pest management. The impact of nematodes on trade extends beyond immediate economic losses; it also influences the agricultural practices and pest management strategies adopted by farmers. The necessity of adhering to stringent regulations often drives the development and adoption of integrated pest management (IPM) strategies that emphasize sustainable practices and the use of resistant crop varieties. Some countries in South America are also investing in research and development to improve the identification and control of nematodes, fostering collaboration among agricultural stakeholders, researchers, and government entities, while others lack sufficient research and extension services due to a shortage of specialists. In conclusion, the regulation and quarantine measures surrounding nematodes are critical to safeguarding agricultural productivity and ensuring trade viability in South America. As global trade continues to expand, the need for harmonized approaches to nematode management will grow, necessitating ongoing research, policy development, and international cooperation. Addressing the challenges posed by nematodes through effective regulation and management will be vital for supporting the agricultural economy and ensuring food security in the region.

## Entomopathogenic nematode tolerance of and preference for salinity and the role of a Na^+^/K^+^ atpase in adaptation to salinity

Achi, Perla^1^, C. McCarthy^1^, L. Bavier^1^, R. Pena^1^, V. Iglesias^1^, P. Christensen^1^, A. Baniya^1^, C. Goldy^1^, R. Adrianza^1^, S. Reddy^1^, S. C. Groen^1,2,†^ and A. R. Dillman^1,2,†^

^1^Department of Nematology, University of California, Riverside, California, United States of America

^2^Center for Infectious Disease and Vector Research, Institute for Integrative Genome Biology, University of California, Riverside, California, United States of America

^†^These authors contributed equally

### Abstract

Soil salinity varies widely due to both natural and human-induced factors, such as road salt, agricultural practices, and drought. It can also result from ecological geography, such as coastal environments. While all species rely on salt to some extent, increasing salinity impacts biological mechanisms, forcing species to adapt and altering multi-trophic interactions. Entomopathogenic nematodes (EPNs) are parasitic roundworms that serve as biological control agents against insect pests and are important in agriculture. EPNs rely on chemical cues in the soil, such as CO_2_, salt, and vibrations, to locate suitable hosts. Meanwhile, insects frequently visit the soil to supplement their plant-based diets with salt (mud-puddling), making them vulnerable to EPN attacks. However, changes in salinity can negatively affect EPN foraging, infectivity, and behavior. For example, *Steinernema riobrave* was found to be negatively impacted by elevated salt levels, reducing its ability to locate hosts, whereas *S. carpocapsae* exhibits higher salt tolerance than *S. feltiae*. Our recent findings suggest parallel evolution of target site insensitivity (TSI) mutations in *S. carpocapsae*, a widely studied generalist species, similar to those found in milkweed-specialized insects. These mutations confer resistance to cardiac glycosides (CGs), potent toxins sequestered by milkweed-feeding herbivores, allowing *S. carpocapsae* to successfully infect these hosts. TSI involves mutations in the Na^+^/K^+^ ATPase pump, a membrane protein essential for ion flow, gradient regulation, and cell signaling. This raises questions about the potential pleiotropic effects of these mutations, particularly in salt tolerance. Our results indicate that *S. carpocapsae* exhibits higher salt tolerance and can more effectively locate and infect insect hosts in high-salinity environments. This was further confirmed using *C. elegans* mutants, demonstrating that TSI mutations contribute to salt tolerance. These findings provide valuable insights into the adaptability of EPNs, their ecological distribution, and applications in biological control.

## Facilitating high throughput nematode community studies and assessing its potential as a standalone method for direct soil analysis

Akanwari, Jerry^1,2^, S. A. H Naqvi^1,2^ and T. Sultana^2^

^1^Brock University, Department of Biological Sciences, ON, L2S 3A1

^2^Agriculture and Agri-Food Canada, London Research and Development Center, Vineland Station, ON, L0R 2E0

### Abstract

Soil extraction methods have been criticized in nematode ecological studies due to low recovery rates and poor specificity of commonly used NF1-18Sr2b primer. To address these limitations, we assessed the performance of NF1-18Sr2b and two degenerate primer sets, NemF-18Sr2b and NemFopt-18Sr2bRopt used for metabarcoding studies. We tested the performers of these primers using DNA extracted directly from the soil (SE) and nematodes isolation before DNA extraction (NE). Our results showed consistently higher proportions of nematode sequences in NE compared to SE regardless of primer type. However, in soil DNA extraction, primer choice significantly affected detection: The commonly used NF1-18Sr2b performed poorly, while NemF-18Sr2b and NemFopt-18Sr2bRopt recovered substantially more nematode sequences. Beyond sequence recovery, differences in extraction method and primer selection influenced taxonomic resolution and detection of key nematode groups. SE approach identified unique families not found in NE, suggesting complementary strengths. Our findings revealed the importance of primer optimization and DNA source in metabarcoding studies. The degenerate primer enhances nematode detection in soil extracts and may bridge the gap between current SE and NE approaches. Optimizing these methodologies could increase ecological inference, reduce sampling inefficiencies, and improve the reliability of nematode community assessments.

## Crop diversification to improve soil health and network structure of nematode communities in Lake Erie Basin

Akanwari, Jerry^1,2^ and T. Sultana^2^

^1^Brock University, Department of Biological Sciences, ON, L2S 3A1

^2^Agriculture and Agri-Food Canada, London Research and Development Center, Vineland Station, ON, L0R 2E0

### Abstract

Canadian agriculture is expanding rapidly in terms of land use, government investment, and production output. As the sector grows, new agricultural practices are being adopted to sustain productivity and minimize environmental impacts. These interventions include the use of summer and winter cover crops, as well as the integration of conservation tillage practices to improve efficiency and sustainability. Our study has been investigating the impact of cover crops (CCs) on soil nematode communities and its impact on soil health within short term vegetable production (spinach) and long term cash crop (corn–soybean) production systems. In spinach cultivation, results show that conventional practices can increase presence of plant-parasitic nematodes (PPNs) in soil by 53%. However, incorporating a mix of cover crops (cowpea and pearl millet) can reduce PPN populations and boost presence of beneficial nematodes by over 70%. In corn–soybean rotations, winter fallow resulted in a 65% increase in PPN populations compared to fields planted with winter CCs. Additionally, winter CC mixtures significantly increased nematodes Shannon diversity, maturity index (MI), and structure index (SI) values relative to fallow sites. Conservation tillage further enhanced soil food web quality, increasing bacterivore populations by 37% in the 0–5 cm soil layer and by 42% in the 5–20 cm layer. Our ongoing research aims to further clarify how these interventions have the potential to improve both economic returns and environmental sustainability in Canadian agriculture.

## Unraveling agricultural management drivers of nematode profiles using a metabarcoding approach

Akanwari, Jerry^1,2^ and T. Sultana^2^

^1^Brock University, Department of Biological Sciences, ON, L2S 3A1

^2^Agriculture and Agri-Food Canada, London Research and Development Center, Vineland Station, ON, L0R 2E0

### Abstract

The Lake Erie Basin is a key agricultural region in the province of Ontario due to its warm and fertile soils. To sustain crop production and reduce agricultural nutrients loss into Lake Erie, growers and policymakers recommend several management practices such as cover crops and conservation tillage. The implementation of winter cover crops (WCCs) and conservation tillage is increasingly adopted as best management practices to improve soil health and reduce nutrients runoff. This study uses a metabarcoding approach to evaluate the impact of these practices on plant-parasitic nematodes (PPNs) and their distribution within the soil profile in corn-soybean rotation. We examined the effects of rye as a WCC and three tillage systems: conventional tillage (CT), minimum tillage (MT) and no tillage (NT) on PPNs at 0–5 cm and 5–20 cm depths over two consecutive years. Results shows that PPNs were generally abundant in the 5–20 cm compared to 0–5 cm, during both the WCC and main crop stages. In 2021, PPN abundance was highest under CT but declined in 2022, whereas NT showed increasing PPN populations in 2022, especially at 5–20 cm depth. CT and MT had higher populations of PPNs during WCC stage compared to the cash crop stage whereas the opposite trend was observed for NT. A total of eleven PPN taxa were identified, with different distributions within the soil profile. For instance, Pratylenchus was significantly higher under CT system and at 5–20 cm across all treatments. Inversely, Ditylenchus showed higher abundance at 0–5 cm in all treatments. Soil properties such as OM, CEC and pH influenced PPN communities. In the CT system, OM was negatively correlated with PPN abundance, whereas CEC and pH showed positive correlations. The study provides key insights into the role of soil disturbance, WCCs and soil properties in shaping PPN populations.

## Irreversible effects of Reklemel™ active on root-knot nematode (*Meloidogyne incognita*) motility, mobility, and infectivity in soil

Baidoo Richard^1^, B. Bangel^1^, T. Thoden^2^ and C. Geng^1^

^1^Corteva Agriscience, 9330 Zionsville Road, Indianapolis, IN 46268

^2^Corteva Agriscience, 81677 Munich, Germany

### Abstract

The effect of a nematicide on nematodes is a function of its concentration and exposure time. The higher the concentration and longer the exposure time, the more effect is expected. Such an effect may either be reversible or irreversible which is also dependent on dose and length of exposure. For reversible effects, the nematode can recover from the nematicide impact if the exposure time is short-lived and for irreversible effects, the nematode cannot recover, and the effects continue even when the nematicide is removed. In this study, we demonstrate the irreversible effects of Reklemel™ Active on Root-knot nematode (*Meloidogyne incognita*) at sub-lethal concentrations relative to other nematicides (Fluopyram, Fluensulfone, Oxamyl, and Abamectin) using a Phenalysys imaging technology. Second stage juveniles (J2s) of RKN were exposed to different sublethal concentrations of the nematicides and the mobility or motility of the nematodes were assessed using a Phenalysys imaging system at different time points. The J2s were washed of the nematicide, and mobility/motility reassessed 1 hour and 24 hours after wash to determine recovery. The results showed that the effect of Reklemel was irreversible and continued to immobilize the nematode after washing. We also evaluated the effect of Reklemel on RKN mobility and infectivity relative to other nematicides. The nematodes were washed after exposure to sublethal concentrations for varying time points and were placed on a filter to allow active nematodes to swim through in a modified Baermann funnel system and then inoculated into pots with young cucumber plants to assess infectivity in soil. The results showed that Reklemel at sublethal concentrations (<=5 ppm) could significantly reduce RKN infectivity in soil even though the RKN may be alive and could pass through coffee filter. The irreversible effects of Reklemel and its ability to reduce RKN infectivity at sublethal doses are significant attributes that differentiate it from other nematicides on the market such as Abamectin, Fluopyram, or Fluensulfone.

## Refining entomopathogenic nematode genomes: advancing biological control through improved genomic resources

Baniya Anil^1^, E. M. Schwarz^2^ and A. R. Dillman^1^

^1^Department of Nematology, University of California, Riverside, 900 University Ave., Riverside, CA 92521

^2^Department of Molecular Biology and Genetics, Cornell University, Ithaca, NY 14853

### Abstract

Entomopathogenic nematodes (EPNs) are a group of parasitic nematodes that serve as effective biological control agents against a variety of insect pests. Beyond their agricultural relevance, EPNs exhibit unique biological features, making them valuable model systems for studying symbiosis, host-parasite interactions, and parasitology. However, progress in understanding their biology and genetic mechanisms has been hindered by the lack of high-quality genomic resources. The recent release of a high-quality genome for *Steinernema hermaphroditum* has paved the way for improved genome assembly and annotation in other EPN species. In this study, we refined the genomes of several key EPNs, including *Steinernema glaseri, Steinernema scapterisci, Steinernema carpocapsae,* and *Heterorhabditis indica*. These newly refined genomes show significant improvements in assembly quality and gene annotation compared to existing public databases, as indicated by enhanced quality scores and completeness metrics. Our genome assemblies not only provide more accurate gene and protein models but also offer insights into the broader microbial communities associated with these nematodes, potentially shedding light on their ecological interactions and symbiotic relationships. These refined genomic resources lay a critical foundation for advancing functional genomics and comparative studies across EPNs. They open new avenues for dissecting the molecular basis of parasitism, symbiosis, and adaptation in these organisms, thereby enhancing their utility both as biological control agents and as model systems in parasitology and evolutionary biology.

## Performance of various sources in dispersal of nematodes

Benetková, Petra^1,2^, L. Háněl^2^, L. Tichý^3^, R. v. Diggelen^4^, C. D Hervilly^5^ and J. Frouz^1,2^

^1^Charles University, Institute for Environmental Studies, Prague, 12800

^2^Czech Academy of Science, Biology Centre, Ceske Budejovice, 37005

^3^Masaryk University, Department of Botany and Zoology, Brno, 61137

^4^University of Antwerp, Ecosystem Management Research Group, Antwerp, 2610

^5^Umeå University, Department of Ecology and Environmental Sciences, Umeå, 901 87

### Abstract

Biodiversity loss, driven by human activities such as resource extraction and significant land-use changes, poses a major threat to ecosystems. Open-cast mining and intensive agriculture, in particular, contribute to soil degradation and the decline in soil fauna diversity. Ecosystem restoration has emerged as a crucial strategy to address these losses, with an increasing focus on soil fauna, particularly nematodes, as indicators of soil health. Some restoration measures use various materials to support target communities in order to mitigate the losses in degraded habitats.

The results of our recent studies show the succession of nematodes during the full restoration of European ecosystems, where various organic amendments were added to the substrate as a source of nematodes. Over time, there has been a noticeable difference in both the quality and quantity of nematode assemblages in restored heathlands. While the relative proportions of trophic groups in experimental plots were similar to those in target habitats, their abundance often did not reach the levels found in reference habitats. Soil water content also played a significant role in shaping the assemblages. Similar results were found in post-mining spoil heaps, where large blocks of meadow soil were installed in the middle of bare substrate. Nematode assemblages resembling those from the source block were found in close proximity; however, the initial phase of primary succession was observed in assemblages further from the source (30 meters away) even after 20 years of development. Assemblages in soil with soil amendments in an already afforested quarry did not differ from those in target habitats, which supports our hypothesis that soil development, or the lack thereof, is a major constraint in the restoration of damaged ecosystems. Intensively managed fields are not typically considered damaged ecosystems; however, their soil food webs are notably depleted. Windbreaks between fields proved to be a significant source of soil fauna, enhancing the fields’ trophic food webs and improving soil biodiversity.

## Nematode illustration master class: understanding, depicting and teaching 3D anatomy of the transparent puzzles we can only see in 2D slices

Bernard, Ernest C and J. Eisenback

Dept. of Entomology & Plant Pathology, University of Tennessee, Knoxville, TN 37996-4560

### Abstract

Nematodes are cylindrical and most of the useful characters are internal, with digestive and reproductive systems obscuring each other. While we can see the separate organs, translating the view onto a flat piece of paper (transillumination) can be difficult. Accuracy in proportions and size is all-important; nematology is strongly dependent upon illustrations to make its points in taxonomy, structure and function. Fortunately, many examples exist that can serve as instruction on 3D illustrative techniques, such as the work of illustrators like W.E. Chambers and A. Coomans, or the drawings in Chitwood & Chitwood’s 1950 book. Style rests with the individual graphic artist, who may depict nematodes as sturdy yeomen of the soil, or as delicate creatures gliding in the pores and crevices. Regardless of style, nematode illustration is an art that requires careful planning and execution, as the rendering of small details often is of great importance. What do you need to draw nematodes? Obviously, one needs paper, vellum or acetate to draw on and pens of several tip diameters and a smooth surface for the paper, such as a drafting table. Pens are a critical feature; Rapidograph or Rotring pens have been used by most artists but frustration with clogging is cumulative. Pigma Micron pens have been satisfactory for me. Observations and sketching or imaging are made more easily with a DIC microscope, which provides an optical depth of field, although a bright-field microscope is quite useful with careful adjustment of light intensity, condenser and iris. A drawing tube is essential for making direct sketches, while digital images at different planes allow for the recognition of finer details. The nematode and its parts should be drawn as seen. Avoid artificially contorting a nematode to make it fit on the sheet. The amount of “embellishment” (e.g., stippling) added to highlight depth or particular structures may provide a more attractive work but should not exaggerate the feature. Digital images are invaluable references for truthing drawings, and if it works for you, make sketches on acetate directly from the screen. Once you begin drawing for publication, be self-critical. Is this good enough for publication? If not fix it if possible (White-Out is quite handy!) or throw it away and start over. For complex 3D drawings, practice what you want to do before committing yourself to the final image. Separate drawings can be cut out and composited on a larger sheet to form a well-balanced plate, then scanned at a high resolution to provide finished illustrations for submission. Alternatively, a plate can be prepared using Adobe Illustrator. The finished plate should have a minimal amount of blank white space.

## Bridging continents in nematology education: call to the world from M.Sc. Program imanema

Bert, Wim, I. Dehennin and W. Decraemer

Ghent University, Department of Biology, Ghent, Belgium

### Abstract

Over the past 30 years, the International Master of Science in Agroand Environmental Nematology (ImaNema; previously known as PINC and EUMAINE) at Ghent University has trained about 500 nematologists, thanks to funding from the Belgian government (VLIRUOS, International Course Programme - ICP) and the European Union. Operational support and scholarships enable students from Africa, Asia, and South America to participate. The master’s program was established specifically in response to the increasing demand from students in these regions for training in nematology, and from its inception, incorporated a scholarship scheme with a strong emphasis on facilitating knowledge transfer to their home countries. Several students returned to their home countries to build local capacity and about 30% pursued doctoral studies, many continuing their PhDs in the US. To meet the sponsor’s requirement to formally establish strategic partnerships with institutions in Belgian partner countries in the Global South, IMaNema revised its curriculum in 2016 to better address the specific needs and challenges of tropical and subtropical regions (VLIR-UOS ICP Connect). This revision led to a successful partnership with NemAfrica (ICIPE and IITA), among others, and the introduction of a mobility track to Kenya, which included internships, field visits, agricultural extension work, and a short training program facilitated by students. In a later phase, Moi University’s MSc Plant Pathology program, with an enhanced nematology curriculum, directly benefited from this partnership, resulting in funding from the USA to train staff from Plant Village in nematology. Another initiative to build further capacity in Sub-Saharan African institutes was “Nematology Education in Sub-Saharan Africa (NEMEDUSSA),” an Erasmus+ Capacity Building in Higher Education project (2021–2024). NEMEDUSSA involved 16 partners and focused on six core activities: (1) Developing and enhancing nematology curricula at BSc and MSc levels; (2) Training staff to strengthen teaching capacity; (3) Upgrading laboratory facilities, particularly microscopy infrastructure, to advance hands-on learning; (4) Creating an open-access Nematology Digital Learning Platform; (5) Establishing a network of nematologists in SSA through webinars and workshops involving diverse stakeholders and; (6) Raising awareness through dissemination activities. Both NEMEDUSSA and the final five years (2022–2027) of the VLIR-UOS ICP Connect project are part of an exit strategy aimed at transferring responsibility for nematology education to higher education institutions in SSA, because IMaNema’s Belgian sponsorship will conclude after 2027. This means that there are no scholarships or operational funds to coordinate the program and its satellite projects, likely resulting in the end of IMaNema as we know it today. This transition presents serious challenges. To sustain the global impact and longstanding tradition of nematology education at Ghent University, it is crucial to explore new partnerships in Europe and beyond, and to redesign nematology education to guarantee the training of the next generation of nematologists. We call upon the global nematology community to engage in shaping the future of nematology education worldwide.

## Biological control of *Meloidogyne incognita* and growth promotion in cotton using drought tolerant plant growth promoting rhizobacteria

Bhandari, Gayatri, P. Chhetri, T. Flowers, B. R. Lawaju and K. S. Lawrence

Entomology and Plant Pathology, Auburn University, Auburn, Alabama

### Abstract

Cotton (*Gossypium hirsutum*) production in Southeastern United States is severely impacted by *Meloidogyne incognita* (Southern Root-Knot Nematode, sRKN), and drought stress, both of which cause significant root damage and yield loss. To address these issues, a study was conducted to evaluate the effectiveness of drought tolerant-plant growth promoting rhizobacteria (DT-PGPR) in reducing nematode populations and improving drought stress. A diverse collection of 235 PGPR strains isolated from plant roots in La Luz, New Mexico were first screened for three traits: nitrogen fixation, siderophore production, and nematicidal activity. Approximately 25% of the isolates demonstrated active nitrogenase function, with *Promicromonospora alba* (DT-15), *Bacillus velenzensis* (DT-59), *Bacillus cabrialesii/inaquosorum* (DT-62), *Paenibacillus endophyticus* (DT-72), *Bacillus amyloliquefaciens/siamensis* (DT-87) emerging as top nitrogen-fixing candidates based on visual calorimetric evaluations using liquid JNFb media. Siderophore production, assessed in Chrome Azurol S (CAS) agar media, showed the highest halo zone in *Pseudomonas monteilii* (DT-14) at 9.8 mm, followed by *Bacillus velezensis* (DT-105, 8.7mm), *Arthobacter nitrophenolicus* (DT-129, 7.3mm), and *Bacillus siamensis* (DT-8, 7.2 mm) as highly competitive strains for iron acquisition—an essential trait for rhizosphere dominance. In vitro nematode bioassays targeting second-stage juveniles (J2) were conducted and their viability was assessed using NaOH viability test after 48 hours. *Chryseobacterium elymi* (DT-3) induced the highest juvenile mortality at 36%, followed *by Sphingomonas kyeonggiensis* (DT-204, 32%), *Bacillus velezensis* (DT-59, 32%), and *Variovorax ureilyticus* (DT-64, 30%) all (α ≤ 0.05) higher than the untreated control. Based on these laboratory findings 37 DT-PGPR strains have been selected for greenhouse trials in Plant Science Research Centre, Auburn University, and seven strains are being evaluated for their efficacy in reducing nematodes populations and promoting cotton growth and yield at the Plant Breeding Unit, Tallassee, Auburn. The selected strains included DT-3 (*Chryseobacterium elymi*), DT-14 (*Pseudomonas piscium*), DT-62 (*Bacillus cabrialesii/inaquosorum*), DT-87 (*Bacillus amyloliquefaciens/siamensis*), DT-105 (*Bacillus velezensis*), DT-129 (*Arthrobacter nitrophenolicus*), and DT-204 (*Sphingomonas kyeonggiensis*). Greenhouse and field trials are arranged as an RCBD with 5 replications and tests are repeated once. The strains were applied at 10^7^ CFU per seed in the greenhouse and as in-furrow sprays at a concentration of 10^7^ CFU applied at 5 Gallons per Acre in the field. Controls consisted of an untreated water control, chemical Velum Prime applied at 6 fl oz/A and biological Majestene applied at a gallon/A. This research will lay the foundation for selecting top-performing strains in sRKN infested fields to assess their practical application in sustainable nematode management through biological control.

## The SCN Coalition: impact, leadership and expansion

Bird, George^1^, S. Markel^2^, G. Tylka^3^, A. Tenuta^4^, C. Bradley^5^, D. Mangel^6^, H. Lopez-Nicora^7^, T. Watson^8^ and J. Johnston^9^

^1^Michigan State University, Department of Entomology, East Lansing, MI 48842

^2^North Dakota State University, Department of Plant Pathology, Fargo, ND 58102

^3^Iowa State University, Department of Plant Pathology, Entomology and Microbiology, Ames, IA 50011

^4^Ontario Ministry of Agriculture, Food and Rural Affairs, Ridgetown, ON

^5^University of Kentucky Research and Education Center, Princeton, KY 42445

^6^University of Nebraska-Lincoln, Department of Plant Pathology, Lincoln, NE 68588

^7^The Ohio State University, Department of Plant Pathology, Columbus, OH 43210

^8^Louisiana State University, Department of Plant Pathology and Crop Physiology, Baton Rouge, LA 70803

^9^MorganMyers, a G&S Agency, Waterloo, IA 50701

### Abstract

The SCN Coalition involves multiple states, private companies and a marketing agency. The initiative employs a dynamic multimedia approach. It has generated more than 437 million potential impressions in the agricultural media and more than 15 million impressions in Facebook, X and LinkedIn. The SCN Profit Checker is an online tool that estimates potential yield and profit loss in individual fields due to SCN reproduction on soybean varieties with PI 88788 SCN resistance. The tool uses an algorithm developed with data from more than 35,000 research plots in which soybean varieties with PI 88788 SCN resistance were grown. This SCN Profit Checker has been used on thescncoalition.com more than 82,400 times between September 2023 and March 2025. Results of quantitative market research indicate that from 2018 to 2024, the outreach efforts of The SCN Coalition activities and messages have been linked to several significant changes in farming practices. There has been a 9% increase in farmer soil sampling for SCN, 17% increase in rotating sources of genetic SCN resistance, 12% increase in use of Peking as an alternative source of resistance and 20% increase in use of nematode-protectant seed treatments. In 2024, The SCN Coalition began the process of transitioning its leadership to a younger generation of nematologists. The new leadership includes Dylan Mangel, Horacio Lopez-Nicora and Tristan Watson. In addition, The SCN Coalition is in the process of expanding its focus to include four taxa: soybean cyst, root-knot, root-lesion and reniform nematodes. New soybean management guides for root-knot, root-lesion and reniform nematodes will be available by September 2025.

## Linking entomopathogenic nematodes natural occurrence with soil health: insight from vineyard soils

Blanco-Pérez, Rubén^1^ and R. Campos-Herrera^2^

^1^Departamento de Suelos, Biosistemas y Ecología Agroforestal, Misión Biológica de Galicia (MBG-CSIC), Pazo de Salcedo, Pontevedra, Spain

^2^Instituto de Ciencias de la Vid y del Vino (CSIC-Univ. de La Rioja-Gobierno de La Rioja), Logroño, Spain

### Abstract

Entomopathogenic nematodes (EPNs) are globally distributed soil organisms and are well-established biological control agents. However, conventional agricultural practices—particularly intensive tillage and agrochemical use—can negatively affect soil biodiversity and EPN populations. Given that vineyards represent one of the most intensively managed agroecosystems, they provide an ideal context to assess the impact of alternative soil management strategies on EPN dynamics and soil health. This study evaluated the occurrence, abundance, and activity of native EPNs under varying viticultural practices, including till/no-till, conventional management, cover cropping, mulching, and organic farming. EPN occurrence was assessed using *Galleria mellonella* baiting and species-specific qPCR targeting 10 EPN species and 12 associated soil organisms, including free-living nematodes (FLNs), nematophagous fungi, and ectoparasitic bacteria. Across multiple vineyard sites, seven EPN species were detected, notably *Heterorhabditis bacteriophora*, *Steinernema feltiae*, and the newly described *S. riojaense*, consistently found in all sampling campaigns. Alternative practices, such as specific cover cropping (spontaneous) and mulching (spent mushroom compost), were associated with increased EPN activity and reduced abundance of potential antagonists, such as nematophagous fungi. The most apparent results were that organic vineyards supported the highest EPN and FLN diversity, with particular species exclusive to these systems, underscoring the positive effect of reduced chemical inputs and tillage on soil biota. These findings were corroborated by a regional High Throughput Sequencing survey of the soil nematode community across 80 vineyards, comparing tilled versus untilled soils and conventional versus organic pest management. The results confirmed that EPNs respond sensitively to management-induced changes in soil ecosystems. Altogether, this work highlights the utility of EPNs as bioindicators of soil health in vineyards and emphasizes the value of integrating molecular tools into agroecological monitoring to guide sustainable viticulture, results that might also be translated to other agroecosystems.

## A soil love triangle: how soil organisms are interacting with soybean yield

Blazek, Amanda^1^, S. Pate^2^, L. Schumacher^3^ and H. Kelly^1^

^1^University of TN, Dept. of Entomology and Plant Pathology, Jackson, TN 38305

^2^LSU AgCenter, Winnsboro, LA 71295

^3^USDA – ARS Agricultural Research Services, Tifton, GA 31793

### Abstract

The soybean cyst nematode (SCN) is a plant-parasitic, microscopic roundworm that causes the most yield loss in soybean production in the United States and Canada. SCN management includes rotating to non-hosts, using SCN resistant cultivars, and nematicides. Although with the emergence and increase in SCN populations that can reproduce on cultivars with the PI88788 SCN resistance, additional management strategies are needed. Winter cover crop mixtures have been promoted to increase soil health and provide some weed management. Although, little research has been conducted in the field of understanding the management practices of cover crops in soybean production systems and their impact on soil organisms, both pathogenic and beneficial nematodes and soil fauna. A field trial was conducted over four seasons (2021–2024) to investigate the impact of cover crop mixtures, cover crop termination timing, and seed treatments on 8 plant-parasitic nematode species (*Heterodera*, *Rotylenchulus*, *Meloidogyne*, *Pratylenchus*, *Helicotylenchus*, *Hoplolaimus*, *Tylenchorhynchus*, *Paratylenchus*), 6 species of beneficial soil organisms (fungivore, bacterivore, omnivore nematodes, oligochaetes, collembola, rotifers, and tardigrades), and soybean yield within a soybean production system in West Tennessee. The trial was conducted across three different sites the first three seasons, where the fourth season was at the same site as the previous year. Two different five-way cover crop mixtures were planted each fall and compared to fallow, and comparison of two cover crop termination timings: April (early-three weeks before soybean planting) and May (late-at soybean planting). Seed treatments evaluated were: fungicide-only, insecticide-only, fungicide+insecticide, and fungicide+insecticide+nematicide. Soil samples were collected at multiple timepoints to assess soil organisms’ reproduction factor (RF). While year had the most significant effect on organisms RF, cover crop biomass, and soybean emergence and yield due to environmental conditions and differences in site locations; seed treatments had slight impact on lesion and SCN RF values. Additionally, the differences between single seasons of cover crop versus two consecutive seasons of cover crops impact on soil organisms and soybean production will be discussed.

## Overcoming resistance: unraveling the mechanisms behind root-knot nematode evasion of tomato MI gene and susceptibility

Blundell, Alison Coomer^1^, P. Shakya^1^, B. Castro^2^, D. Dai^2^, M. Winter^3^, D. Lunt^3^, V. M. Williamson^1^ and S. Siddique^2^

^1^University of California Davis, Dept. of Plant Pathology, Davis, CA, USA

^2^University of California Davis, Dept. of Entomology and Nematology, Davis, CA, USA

^3^School of Nature Natural Sciences, University of Hull, Hull, UK.

### Abstract

California is the lead producer of processing tomato worldwide. The success of this industry depends on growers’ abilities to implement management strategies such as integrated host resistance, effective pesticides, and non-host rotation crops to eliminate or control pathogens. Despite these efforts, root-knot nematodes (RKNs), *Meloidogyne* spp., cause an estimated 5% yield loss in processing tomatoes by suppressing the plant immune system, damaging root tissues, and creating entry points for secondary pathogens such as *Fusarium* species. These pathogen complexes result in a severe yield loss for growers each year. For decades, the resistance gene *Mi-1* has effectively detected and inhibited RKNs in tomatoes, but the underlying mechanisms by which it recognizes these pathogens remain largely unknown. However, resistance-breaking populations have been increasingly identified in both greenhouse and field settings, threatening the effectiveness of the *Mi-1* gene and consequently the tomato industry. To better understand how RKNs overcome Mi-1 resistance, we utilized two closely related strains of *Meloidogyne javanica*: VW4, a wild-type strain, and VW5, a lab-generated resistance-breaking strain. Through comparative genomics, we identified four nematode avirulent gene candidates that may interact with Mi-1. In addition, to assess the distribution of resistance-breaking RKNs and identify “hotspots” in California, we collected 14 field isolates from processing tomato fields across the state. These isolates are being confirmed to be resistance-breaking and are undergoing purification by single egg mass isolation. Confirmed resistance-breaking isolates will be used in infection assays on current rotation crops for processing tomatoes. With this research, we aim to improve understanding of how RKNs evade Mi-1 resistance and develop management strategies to combat these resistance breaking populations, ultimately supporting California’s tomato growers.

## Rapid identification of *Meloidogyne hapla* using species-specific primer

Borrego-Benjumea, Ana^1^, J. Akanwari^1,2^ and T. Sultana^1^

^1^Agriculture and Agri-Food Canada, London Research and Development Center, Vineland Station, ON, L0R 2E0

^2^Brock University, Dept. of Biological Sciences, St. Catharines, ON

### Abstract

Root-knot nematodes (*Meloidogyne* spp.) are among the most economically important plant-parasitic nematodes globally, with *Meloidogyne hapla*, the northern root-knot nematode, posing a significant threat in Canada by causing substantial yield losses in vegetable and ornamental crops. Morphological identification of *M. hapla* is often difficult due to its similarity with closely related species, such as *M. incognita* (Southern root-knot nematode), and traditional diagnostic methods require taxonomic expertise and are prone to error. To address these limitations, this study developed and validated a PCR-based molecular identification system using species-specific primers targeting the mitochondrial NADH subunit 5 (Nad5) gene, a highly variable and reliable region for nematode differentiation. Nad5 sequences from eight closely related *Meloidogyne* species were retrieved from GenBank and aligned using Geneious Prime to identify conserved regions flanking variable sites. Based on these alignments, several sets of forward and reverse primers were designed with optimal melting temperatures, lengths, and minimal secondary structures. DNA was extracted from pure cultures of *M. hapla* and from other *Meloidogyne* species (*M. arenaria*, *M. chitwoodi*, *M. enterolobii*, *M. incognita*, *M. javanica*, and *M. marylandi*) to evaluate primer specificity. The primer set 408F/1360R was selected for its consistent amplification of a 960 bp fragment uniquely from *M. hapla* DNA. PCR assays using this primer set showed no amplification in negative controls or with DNA from other *Meloidogyne* species, and cross-reactivity tests with mixed DNA templates confirmed the high specificity of the assay. The amplicons were sequenced and verified through BLASTn analysis, further supporting their species specificity. This PCR-based method provides a rapid, accurate, and reliable tool for identifying *M. hapla*, overcoming the limitations of morphological techniques. Its implementation can significantly enhance nematode diagnostics and monitoring, allowing for early detection and targeted management.

## Using papaya ground seed to induce systemic resistance against root-knot nematodes in Hawai‘i

Braley, Lauren and K.-H. Wang

University of Hawai‘i at Manoa, Honolulu, HI 96822

### Abstract

Plant-parasitic nematodes (PPN) pose an estimated $80 to $173 billion crop yield loss globally every year. In Hawai‘i, PPN pressure is high year-round, and the high cost of agriculture production is driving food producers in Hawai‘i to seek local resources for pest management. Papaya seeds are considered an agricultural waste in Hawai‘i, but they have been shown to possess biofumigation properties against PPN. The objective of this study was to explore the induced systemic resistance (ISR) potential of ground papaya seed (PGS) against RKN infection, while maintaining soil health. Two greenhouse pot trials and two field microplot trials were conducted to compare PGS crude extract (CE) as a soil drenching solution at 0.5% and 1% concentrations to unamended (NA) and autoclaved (greenhouse) controls. Greenhouse trials were conducted using field soil frozen for 1 week prior to use to eliminate existing populations of PPN. Roots of ‘Orange Pixie’ tomato seedlings were split and placed into two separate pots, with 4 split-pot replicates per treatment. One side of the roots was drenched with PGS CE or water (control), while the other side of the roots was inoculated with 200 *Meloidogyne incognita* (*Mi*) second stage juveniles (J2) 24 hours post-drenching. Drenching was repeated every 2 weeks for 2 months. Root tissue from the inoculated side of the pot was collected at 1, 2, and 7 days after inoculation for downstream RT-qPCR analysis. Field microplot trials were conducted using 38-L fabric pots (without splitting roots) in field soil naturally infested with RKN. Soil samples were assayed for RKN and nematode communities at transplanting, 6 weeks, and 3 months after planting (termination of experiment). In greenhouse split-pot trials, a significant reduction in the number of RKN females in the roots was observed in both PGS CE concentrations, with the greatest decrease occurring in 1% PGS CE. However, the number of J2s penetrating the roots was only decreased in 1% PGS CE. Though 0.5% PGS CE did not reduce J2 penetration, it prevented RKN from developing into the reproductive stage. The split-root assay also supported that ISR was triggered by PGS CE treatment. In Field Trial 1, initial PPN population numbers were lower (P_i_=37) compared to Field Trial 2 (P_i_=402), which led to lower gall development in Field Trial 1. However, consistent trends were observed across both trials, with PGS CE treated plants showing reduced galling and PPN abundance in the soil. PGS CE treatments additionally increased soil microbial respiration (Solvita Burst Test, Woods End Labs, LLC, Augusta, ME) and the abundance of bacterivorous nematodes (*P* ≤ 0.05), suggesting enhancement of soil nutrient enrichment. Omnivores and predators were unaffected between treatments (*P* ≤ 0.05). Overall, PGS CE not only reduced RKN penetration, delayed J2 development, activated ISR, and enriched soil nutrients, but it also posed no threat to soil health, making it a good potential use of agricultural waste.

## A close look into the nematode certification for ornamental plants implemented by the Florida Department of Agriculture and Consumer services-division of plant industry

Brito, Janete A, R. N. Inserra, S. Vau and L. Whilby

Florida Department of Agriculture and Consumer Services – Division of Plant Industry, Gainesville, FL 32608

### Abstract

The regulatory activities mandated by Florida Statutes to the Florida Department of Agriculture and Consumers Services (FDACS) include the implementation of the Nematode Certification Program for Citrus Nurseries. This program has been operated for over 55 years by the Nematology Section of the Division of Plant Industry (DPI). It was initiated due to an outbreak of a devastating citrus disease, “spreading declining” caused by the burrowing nematode (BN), *Radopholus similis*. The program aims to ensure that the production of citrus propagative planting material is free of BN and other nematode parasites of citrus. A second nematode certification program was established almost concomitantly for the Ornamental Industry with the aim to produce ornamental plants and plant parts free from BN, other regulated nematode species, and ensure compliance with national and international export requirements. The operation of these programs involves a series of coordinated actions by plant nurseries and DPI. To obtain the nematode certification, ornamental nurseries must be registered with the State, sign a compliance agreement and follow specific guidelines issued by FDACS and the importing states and/or countries. Bureau of Plant Inspection’s field trained nematode inspectors collect samples consisting of soil, roots and other plant parts from nurseries and submit them to the Nematology Section for nematological analysis using a tracking system (Laboratory Identification Sample Tracking - LIST), which registers sample information submitted by the inspectors, analysis results and the final report either granting or denying certification to the sampled nurseries. Six plant inspectors across Florida are exclusively dedicated to sample collection for nematode certification. Samples are processed by laboratory technicians (3) and morphological identifications of nematode species performed by nematologists (3). Additionally, molecular identifications are performed for samples found to be positive for *Meloidogyne* spp. For the fiscal years 2022–2024, out of Florida’s 20,573 registered nursery’s locations, 2,579 ornamental nurseries were sampled, resulting in the collection and analyses of 15,776 samples from ornamental plants. A smaller number of samples were collected and analyzed from citrus nurseries in hermetic greenhouses.

## Challenges and opportunities for international collaboration

Bui, Hung^1^, J. Desaeger^1^, Nguyen T. T. Nga^2^ and Tran V. Phen^2^

^1^University of Florida, Department of Entomology and Nematology, Gulf Coast Research and Education Center, Wimauma, FL 33598, USA

^2^Department of Plant Protection, College of Agriculture, Can Tho University, Can Tho City 90000, Vietnam

### Abstract

The Vietnamese Mekong Delta plays a very important role in agricultural production, particularly for rice, tropical fruits, and vegetables. However, plant-parasitic nematodes (PPNs) pose a serious threat to the region’s productivity, soil health, and long-term sustainability. In recent years, nematode-related crop losses have intensified, placing greater pressure on growers and raising concerns about regional food security. Several damaging PPN species are prevalent across this region. *Meloidogyne incognita* causes severe damage in banana, dragon fruit, black pepper, ginger, cucumber, coffee, and jackfruit. *Pratylenchus coffeae* is common in citrus, durian, coffee, banana, sugarcane, mango, jackfruit, star apple, pineapple, and peanut. *Rotylenchulus reniformis* damages black pepper, banana, coffee, guava, jackfruit, and pineapple. *Hirschmanniella oryzae* and *Meloidogyne graminicola* are major pests and significantly reducing rice yields. Additionally, these nematodes compromise root systems, weaken plant vigor, and increase vulnerability to secondary pathogens. Despite the widespread impact, research and management efforts in this region remain limited. There is an urgent need for science-based, regionally adapted strategies to effectively manage nematodes which demand expertise and international collaboration. Developed countries with advanced nematology programs can contribute critical expertise, tools, and technologies through joint research initiatives. Areas of collaboration may include diagnostics, nematode biology, host resistance, and the development of both chemical and biological control measures. Collaborative development of integrated nematode management (INM) strategies, tailored to the crops and conditions of the Vietnamese Mekong Delta, can significantly reduce nematode pressure while minimizing environmental impacts. Many tropical and subtropical regions face similar nematode threats, especially as global demand for high-value vegetables and fruits continues to grow. The Vietnamese Mekong Delta can serve as a model for collaborative research and implementation of practical, scalable solutions. Through collaborative international efforts such as research, technology transfer, and capacity building, countries can work together to combat nematode-related crop losses. These partnerships not only enhance agricultural resilience in Vietnam but also contribute to food security and sustainable agriculture on a global scale.

## Effect of soil storage duration and extraction method on nematode recovery from a Florida sandy soil

Bui, Hung^1^, D. Jacobs^1^, C. Xie^1^, H. Q. Nguyen^2^ and J. Desaeger^1^

^1^University of Florida, Department of Entomology and Nematology, Gulf Coast Research and Education Center, Wimauma, FL 33598, USA

^2^Department of Civil and Environmental Engineering, University of South Florida, Tampa, FL, 33620, USA

### Abstract

Soil nematodes are key bioindicators of soil health and integral players in agroecosystem functioning. Free-living nematodes (bacterial feeders, fungal feeders, omnivores, FLNs) facilitate nutrient cycling through the breakdown of organic material. In contrast, plant-parasitic nematodes (PPNs) negatively impact plant health by feeding on roots, often leading to reduced crop yields. Accurate quantification of these nematodes is critical for understanding soil ecology and for implementing effective integrated nematode management strategies. However, nematode recovery is influenced by various factors, including sampling time, extraction method, and storage duration between sampling and processing. The experiment was conducted in Fall 2023 and Spring 2024 at the University of Florida Gulf Coast Research and Education Center (GCREC). Soil was collected from 12 vegetable field plots and each sample comprised of 20–25 soil cores (30 cm deep, 2.5 cm in diameter) collected in a zigzag pattern, resulting in approximately 3 kg of soil per sample. Before storage, each soil sample was thoroughly homogenized and sealed in a plastic ziplock bag, then stored at 5°C in a cooler. Nematode extractions were performed at five storage intervals: 0, 14, 30, 60, and 90 days after storage (DAS). Two extraction methods were used: (1) an active method, the modified Baermann pan technique by using a salad spinner bowl and basket (SS) and (2) a passive method, the sugar flotation technique by using a centrifuge (SF). Extracted nematodes were enumerated and categorized as either FLNs (bacterial feeders, fungal feeders, omnivores) or PPNs (herbivores). Additionally, PPNs were further identified to the genus level (lesion, root-knot, ring, sting, stubby root and spiral nematodes) using morphological characteristics. A fixed-effect model and twoway ANOVA revealed that two main factors (extraction method and storage duration) significantly influenced nematode recovery for both FLNs and PPNs. SF extraction recovered significantly higher PPNs than SS. For extracted FLNs, SS was more effective when samples were processed immediately (DAS = 0), while SF was more efficient after longer storage durations (DAS = 90). Generalized Additive Models (GAMs) identified optimal recovery windows: DAS = 0 for SS and DAS = 90 for SF in the case of recovering FLNs, and DAS = 43.5 for SF in the case of recovering PPNs. Survival curves confirmed these trends, with a crossover point at DAS = 48 for FLNs, and consistent SF dominance for extracted PPNs. The estimated decline rates (DF) were: DF_SF_ = −0.0005 and DF_SS_ = 0.012 for FLNs and DF_SF_ = 0.01 and DF_SS_ = 0.025 for PPNs. These findings indicate that (a) immediate processing and extraction of nematode samples is ideal, particularly with SS technique, and (b) when storage is required, SF offers better nematode recovery.

## Plant-parasitic nematodes in the Vietnamese Mekong Delta: methanolic extracts of sweet orange pomace for *Meloidogyne javanica* control

Calandrelli, Angelica^1^, A. Miamoto^1^, F. A. Pereira^2^, D. C. Baldoqui^2^ and C. R. Dias-Arieira^1^

^1^State University of Maringá (UEM), Graduate Program in Agronomy, Maringá, Paraná, 87020-900, Brazil. crdarieira@uem.br

^2^UEM, Graduate Program in Chemistry, Maringá, Paraná, 87020-900, Brazil

### Abstract

Industrial waste from sweet orange (*Citrus sinensis*) juice pressing holds potential for controlling plant diseases due to its interesting chemical composition. However, there is limited information on the effects of sweet orange pomace on nematode management. This study aimed to evaluate the effect of the methanolic extract and fractions of *C. sinensis* pomace on *Meloidogyne javanica* egg hatching in vitro and nematode reproduction on soybean. For this, *C. sinensis* pomace was dried at room temperature and ground in a blender. A 200 g aliquot was extracted with methanol (p.a.) by exhaustive maceration. Samples were dried in a rotary evaporator at 35 °C under reduced pressure, yielding 7.68 g of crude methanolic extract (CME). For fractionation, 6.65 g of CME was dissolved in 500 mL of a 1:1 (v/v) mixture of water/methanol and partitioned with organic solvents of increasing polarity (hexane, dichloromethane, and ethyl acetate). The solvents were removed by rotary evaporation at 35 °C to give the hexane (HXF), dichloromethane (DCMF), ethyl acetate (EAF), and hydromethanolic (HMF) fractions. In the hatching test, fresh pomace, dried pomace, CME, HXF, DCMF, EAF, and HMF were tested at concentrations of 0 (water), 0.5%, 1.0%, and 1.5%. The assay was conducted in microtubes, and egg hatching was evaluated after 10 days of incubation at 28 °C. Nematode reproduction was evaluated under greenhouse conditions using the same treatments at a concentration of 1.5%. Treatments were applied in furrow at sowing or via seed treatment. Plants were inoculated with 2000 nematodes and evaluated at 60 days after inoculation. All treatments and concentrations reduced egg hatching compared with the control. HXF, HMF, dry pomace, and fresh pomace were the most effective, promoting reductions of 73.8%, 72.4%, 72.1%, and 69.0%, respectively. All treatments decreased *M. javanica* reproduction on soybean, in particular DCMF (74.5%) and EAF (67.6%). Seed treatment was more effective than in-furrow application. Thus, it can be concluded that *C. sinensis* pomace extract has potential to control *M. javanica.*

## Entomopathogenic nematodes in transition: from soil-dwelling biocontrol to versatile pest management tools

Campos-Herrera, Raquel

Instituto de Ciencias de la Vid y del Vino (CSIC-Univ. de La Rioja-Gobierno de La Rioja), Logroño, Spain

### Abstract

Entomopathogenic nematodes (EPNs) represent a well-established group of biological control agents with proven efficacy against a wide range of soil-dwelling insect pests. Their insecticidal activity is mediated through a mutualistic association with symbiotic bacteria, which release potent secondary metabolites that induce rapid host mortality. Historically, EPN applications have focused on below-ground pest control, with numerous successful implementations across diverse crop systems. However, recent advances in formulation technologies and aerial delivery methods have enhanced the environmental resilience of EPN infective juveniles, enabling their application in above-ground settings and expanding their potential across novel agroecosystems. Beyond the nematodes themselves, increasing attention is being directed toward the biocontrol potential of their bacterial symbionts and associated metabolites, particularly for managing pest species that are inaccessible to nematode infection, such as the spider mite *Tetranychus urticae*. In parallel, the global shift toward sustainable agriculture and reduced reliance on synthetic pesticides is driving renewed interest in EPN-based solutions. Integrated approaches that combine EPNs with other beneficial soil organisms—such as mycorrhizal fungi, *Pseudomonas* spp., and earthworms—are also under investigation, with evidence suggesting additive or synergistic effects that may enhance pest suppression. Overall, these findings highlight the versatility of EPNs and their symbiotic bacteria as multifaceted biocontrol tools and reinforce their strategic value in developing sustainable and ecologically sound pest management practices.

## Friend or foe: deciphering symbiosis in nematode-soil microbe interactions

Casey, Veronica^1^, M. D. Carter^2^, C. Allen^2^, J. C. Hong^3^, T. Lowe-Power^4^ and S. Siddique^1^

^1^University of California, Davis, Department of Entomology and Nematology, Davis, CA 95616

^2^University of Wisconsin-Madison, Department of Plant Pathology, Madison, WI 53706

^3^USDA ARS United States Horticultural Research Laboratory, Fort Pierce, FL 34945

^4^University of California, Davis, Department of Plant Pathology, Davis, CA 95616

### Abstract

A disease complex occurs when two pathogens infect the same plant, resulting in overlapping symptoms and increased disease severity. One such example is the co-infection of bacterial wilt (*Ralstonia* spp.) and root-knot nematodes (RKNs; *Meloidogyne* spp.) in crops such as tomatoes, chili peppers, potatoes, and other species. Bacterial wilt is caused by a complex of *Ralstonia* species, which enter the plant roots and colonize vascular tissues. *Ralstonia* bacterial cells move through cortical intercellular spaces until they reach the xylem vessels, where they multiply rapidly. High populations of the bacteria in the vascular system leads to wilting symptoms in infected plants. RKNs also affect the plant’s vascular system by inducing the formation of xylem and phloem elements around the giant cells for nutrient acquisition. Both *Ralstonia* and RKNs have a broad host range and are globally distributed in soil and water. Dual infection by these pathogens has been found to increase disease symptoms even in bacterial wilt resistant rootstocks. Previous studies suggest that RKN infection increases bacterial wilt severity through mechanical wounding of the roots and by inducing plant physiological changes. Despite reports of the disease complex and efforts to manage both pathogens, the exact mechanism of interaction between RKNs and *Ralstonia* remains unknown. We hypothesize that prior RKN infection increases bacterial wilt severity by physically carrying bacteria into the roots and by enabling bacteria to proliferate in newly formed xylem around the feeding site. This builds on previous studies indicating potential phoretic attachment of *Ralstonia* cells to RKN juveniles. Two greenhouse trials conducted in Florida have provided preliminary insights into the interaction between *M. incognita* and *R. pseudosolanacearum* in tomato plants. In both trials, RKN second-stage juveniles were inoculated one week prior to *Ralstonia* inoculation. Two weeks after *Ralstonia* inoculation, the RKN and *Ralstonia* co-infection treatments resulted in near-total wilting in RKN susceptible and resistant plants. Additionally, we conducted in vitro attachment assays to test whether *Ralstonia* attaches to the nematode. We utilized microscopy to visually observe if a fluorescent protein tagged *Ralstonia* strain is clustered around the RKN juveniles. Due to the significant economic impact of RKNs and bacterial wilt disease in the United States, we aim to characterize the disease complex to support effective management strategies.

## Biotechnology approaches against Root-Knot and Root-Lesion Nematodes

Castro Esparza, Bardo^1^, D. Dai^1^, Y. Zhang^1^, R. Latina^1^, P. J. Brown^2^, C. A. Leslie^2^ and S. Siddique^1^

^1^University of California, Davis, Department of Entomology and Nematology, Davis, CA, 95616, USA

^2^University of California, Davis, Department of Plant Sciences, Davis, CA, 95616, USA

### Abstract

Plant parasitic nematodes (PPNs) are a major problem for agriculture, with about 15% of total crop losses worldwide. In California, Root-Knot Nematodes (RKNs) and Root-Lesion Nematodes (RLNs) are of particular importance due to their devastating impact on the tomato and walnut industry, respectively. Currently, there are no ideal strategies to halt RKN and RLN infections. Management strategies for these PPNs include the use of nematicides and resistance genes like *Mi-1*. However, these strategies have limitations, such as the emergence of *Mi-1* resistance breaking strains for RKNs and more stringent nematicide regulations for RLNs. Although RKN and RLN differ in their parasitic cycles, understanding how they acquire nutrients to complete their life cycles provides us with strategies for management through biotechnology. In these projects we are utilizing biotechnology tools to develop new management strategies against RKNs and RLNs in tomatoes and walnuts. To target RKNs, we have performed transcriptional analysis of tomato galls to identify plant susceptibility genes (S genes) that are involved in giant cell maintenance and nutrient uptake in tomatoes. We used gene editing techniques to precisely target these candidate S genes in tomatoes and to test their effect on RKN infections. To target RLNs, we assembled the genome of *P. vulnus* and performed stage specific RNA sequencing. Utilizing this new genomic and transcriptomic information we have identified genes important during all infectious life stages of *P. vulnus*. We plan to use host-induced gene silencing (HIGS) in walnut rootstocks to target *P. vulnus* and reduce their ability to cause disease. To this end we are targeting genes important for cell wall breakdown and motility of *P. vulnus* which are important factors for RLN infections. Currently we are testing our targets through RNAi soaking in walnuts utilizing a rapid tube screening method. We expect that these approaches will yield new insights into genes and strategies to manage RKNs and RLNs.

## Minimal risk and potential benefits of pennycress as a winter oilseed crop for managing soybean cyst nematode in soybean rotation systems in the Northern climates

Chen, Senyu^1^, C. Hoerning^2^, F. Heydari^1^, J. X. Zhang^2^, J. A. Anderson^2^, M. Hunter^2^, S. Wells^2^ and D. Wyse^2^

^1^University of Minnesota, Southern Research and Outreach Center, Waseca, MN 56093

^2^University of Minnesota, Department of Agronomy and Plant Genetics, St. Paul, MN 55108

### Abstract

Pennycress (*Thlaspi arvense*) is an annual weed species in the Brassicaceae family. Previous greenhouse studies showed that pennycress is a moderate host of the soybean cyst nematode (SCN, *Heterodera glycines*), a major pathogen of soybean (*Glycine max*). Pennycress is currently being domesticated as a potential winter oilseed crop. Incorporating winter oilseed crops into the cropping systems offers ecosystem and productivity benefits versus not seeding a cover crop. However, its impact on SCN populations must be assessed before incorporation into the soybean rotation systems of the North Central U.S. Since 2016, we have conducted multiple field experiments in Minnesota to evaluate the impact of pennycress relayed in soybean-corn (corn interseeded into overwintered pennycress) and corn-soybean (soybean interseeded into pennycress) rotations on SCN population dynamics. Results showed no measurable effect of pennycress on SCN population densities within these cropping systems. In a separate overwintering experiment, SCN second-stage juveniles (J2) survived in soil under southern Minnesota winter conditions during 2024–2025, while later developmental juvenile stages (J2–J4) within pennycress roots did not. Our findings also indicate that pennycress can support a single generation of SCN reproduction in the spring-summer period prior to harvest. Overall, our results suggest that pennycress poses minimal to no risk for SCN proliferation in soybean production in Minnesota. Moreover, because J2 can survive in soil but J2-J4 in pennycress roots do not, pennycress may serve as a potential SCN trap crop, especially if early-maturing varieties are developed and used to prevent the completion of one SCN generation. Additionally, terminating pennycress by herbicide prior to SCN reproduction may provide a viable SCN management strategy. Further research is needed to assess SCN overwinter survival across multiple years.

## Comprehensive screening of common bean (*Phaseolus vulgaris*) germplasm reveals diverse resistance to soybean cyst nematode across multiple Hg types

Chen, Senyu^1^, A. Shi^2^, T. E. Michaels^3^, C. Johnson^1^, W. Gottschalk^1^ and H. Xiong^2^

^1^Southern Research & Outreach Center, University of Minnesota, Waseca, MN 56093, USA

^2^Department of Horticulture, PTSC316, University of Arkansas, Fayetteville, AR 72701, USA

^3^Department of Horticultural Science, University of Minnesota, St Paul, MN 55108, USA

### Abstract

The soybean cyst nematode (SCN, *Heterodera glycines*) has become a significant threat to common bean (*Phaseolus vulgaris*) production. Host genetic resistance offers an effective strategy for SCN management in the crop. Since 2017, we have evaluated 1,198 common bean germplasm accessions, including 504 accessions tested with single plant per accession only, from the USDA GRIN collection, representing approximately 10% of the total common bean accessions. Of the 694 accessions tested with replicated plants, 90 were identified as resistant with Female Index (FI) less than 10, and 260 as moderately resistant (10 ≤ FI < 30) to one or more of five SCN populations from Minnesota (MN). No SCN-resistant genotypes were found in MN common bean breeding program. Cluster analysis based on FI values revealed distinct groups of accessions with varying resistance profiles, suggesting the presence of genetic diversity in SCN resistance. Six accessions demonstrated strongest resistance across multiple SCN populations, highlighting their potential as sources for developing broadly adapted SCN-resistant cultivars. Among them, PI 313733, a cultivated accession selected for high yield, exhibited exceptional resistance to all six races of 24 SCN inbred lines from MN, with an average FI of 0.4 (range: 0–2.4), making it a promising candidate for breeding SCN-resistant, high-yielding cultivars. Based on the data of resistance to different SCN HG Types, the mechanisms of SCN resistance in common bean appeared to be different from that in soybean. However, inconsistencies in resistance across SCN populations and test conditions underscore the need for further testing under uniform conditions. As only approximately 10% of the USDA common bean collection has been evaluated, continued screening efforts are essential to explore the remaining germplasm and identify the best sources of SCN resistance for integration into breeding programs.

## Salt-tolerant PGPR: a dual approach to suppressing *Meloidogyne incognita* and promoting cotton production

Chhetri, Prativa, G. Bhandari, T. Flowers, B. R. Lawaju and K. S. Lawrence

Entomology and Plant Pathology, Auburn University, Auburn

### Abstract

Southern root-knot nematode (*Meloidogyne incognita*, sRKN) continues to threaten cotton production, prompting the need for sustainable biological solutions. This study explored the potential of salt-tolerant plant growth-promoting rhizobacteria (ST-PGPR) to simultaneously suppress sRKN and enhance cotton growth via nitrogen fixation, siderophore production, and direct nematicidal activity. A total of 219 ST-PGPR strains, isolated from saline environments in Gulf State Park, Alabama, were revived on tryptic soy agar and screened in vitro. Nitrogen fixation was assessed using nitrogen-free JNFb media by monitoring pH-induced color changes and pellicle formation. At the same time, siderophore production was evaluated on Chrome Azurol S (CAS) agar through halo formation. Nematicidal potential was determined via in vitro mortality assays against *M. incognita* J2s using bacterial suspensions at 1 × 10^7^ CFU/ml. Mortality data were analyzed using a generalized linear mixed model (PROC GLIMMIX), and treatment means were compared with Tukey’s HSD test (α ≤ 0.05). The strains *Curtobacterium oceanosedimentum* (ST-42), *Bacillus velezensis* (ST-68), *Pseudomonas neuropathica* (ST-177), and *Pseudomonas koreensis* (ST-217) demonstrated strong nitrogenase activity, with 38% of isolates showing notable fixation. Siderophore assays (indicative of iron-chelating activity) identified *Pseudomonas glycinae/kribbensis* (ST-170/211), *Bacillus velezensis* (ST-182), *P. koreensis* (ST-162), and *P. anguilliseptica* (ST-161) as highly efficient producers, with ST-170 forming halos up to 1.42 cm. In nematicidal assays, *Psychrobacter nivimaris* (ST-97), *Isoptericola halotolerans* (ST-105), *Bacillus vietnamensis* (ST-167), and *Bacillus safensis* (ST-172) caused the highest J2 mortality of 97, 97, 95 and 94 % respectively. Based on their strong in vitro performance for nematicidal activity, nitrogen fixation, and siderophore production, 38 top-performing ST-PGPR strains were selected for greenhouse evaluation using the cotton cultivar PHY 340 W3FE. In the greenhouse trials, each strain will be assessed for its ability to suppress sRKN infection and promote cotton growth, measured by plant biomass, height, and nematode egg counts per gram of root. In addition to the greenhouse experiment, the seven most effective strains were subsequently selected for large-scale field trials at the Plant Breeding Unit (PBU) at the E.V. Smith Research Center, Auburn University, to validate their biocontrol efficacy and plant growth-promoting as well as yield effects under natural field conditions. The greenhouse and field trials are ongoing. This study evaluates Salt tolerant-PGPR with the goal of selecting multifunctional bioinoculants, offering a sustainable, dual-action strategy for enhancing cotton growth and yields while reducing sRKN pressure.

## A new cyst nematode of the genus *Globodera* from a wild sage *Salvia polystachia* and molecular characterization of two species of this genus from Mexico

Cid del Prado Vera, Ignacio^1^, J. A. M. Ceron^1^ and S. A. Subbotin^2^

^1^Colegio de Postgraduados, 56230, Montecillo, Mexico

^2^Plant Pest Diagnostic Centre, California Department of Food and Agriculture, 3294 Meadowview Road, Sacramento, CA 95832, USA

### Abstract

In autumn 2024, during nematological surveys of solanaceous plants in several states of Mexico, a new *Globodera* species, golden potato cyst nematode *G. rostochiensis* and the Mexican cyst nematode *G. mexicana* were found in soil and root samples. Cysts of a new *Globodera* species was collected from roots of a wild sage *Salvia polystachia* (family Lamiaceae) growing in a forest in Tecalco, Mexico State. The new species was characterised by small cyst size (L=220–470 µm), small Granek ratio (0.3–2.3) and number of cuticular ridges between vulva and anus ranged from 3 to 9. The second-stage juvenile of the new species had 350–580 µm in length with stylet 20–24 μm long. *Globodera rostochiensis* was found from potato fields and wild potato plants, *Solanum demissum* growing at the edge of these fields in Mexico State. *Globodera mexicana* was collected from roots of the pubescent nightshade *S. pubigerum*. New ten D2-D3 of 28S rRNA, six ITS rRNA, fourteen *COI* and twelve *cytb* gene sequences from seven samples of three species were obtained in this study. The gene sequences clearly differentiated a new *Globodera* species. from all other *Globodera* species. Phylogenetic relationships of a new *Globodera* species, *G. rostochiensis* and *G. mexicana* from Mexico with other *Globodera* species and some representatives of the subfamily Punctoderinae were given based on the analysis of four gene sequences. In the ITS rRNA gene tree, sequences of a new *Globodera* species sequences showed a sister relationship with those of the *Globodera* clade parasitising solanaceous plants. It has been suggested that the Sierra Madre Mountains could be considered as one of the centers of diversity and origin for the genus *Globodera*.

## Evolution of MI1 resistance breaking in natural populations of *Meloidogyne incognita*

Collison, Nate and S. C. Groen

University of California-Riverside, Department of Nematology, Riverside, CA, 92507

### Abstract

Plant parasitic nematodes, including the southern root knot nematode *Meloidogyne incognita*, are among the most devastating crop pests worldwide, inflicting an estimated >$150 billion USD in damages to major crops each year. Introgression of resistance (R) genes to crops is a sustainable way to protect crops from nematode infection without relying on environmentally harmful pesticides. The *Mi*1 R gene has been widely introgressed into tomato cultivars and can confer resistance to infection by *M. incognita*. Unfortunately, *Mi*1 resistance has not been durable in agroecosystems. Many cases of *Mi*1 resistance breaking by *M. incognita* have been reported. Our preliminary data shows that different *Mi*1-virulent *M. incognita* isolates have varying levels of virulence on tomato and other crops, which suggests these isolates possess distinct genetic backgrounds. To understand the underlying genetic basis of *Mi*1 adaptation by *M. incognita*, we performed whole genome sequencing (WGS), RNA sequencing, and fitness assays of 14 different isolates of *Mi*1-virulent *M. incognita* collected from field populations throughout California. I will present analysis of the WGS and RNA sequencing data to address questions about the biology of *Mi*1 resistance breaking – 1) did *Mi*1 resistance breaking evolve once, evolve convergently, or evolve multiple times via different genetic mechanisms; 2) what are the genetic networks that underlie *M. incognita* virulence; 3) does adaptation to *Mi*1 resistance inflict tradeoffs in nematode virulence on alternative hosts? These data will provide novel insight to the evolution of virulence in parasites and can inform biotechnological strategies to protect crops from *M. incognita* infection.

## Food security and the role of nematodes in Sub-Saharan Africa

Coyne, Danny^1^ and S. Haukeland^2^

^1^International Institute of Tropical Agriculture, Nairobi, Kenya

^2^International Centre of Insect Physiology and Ecology, Nairobi, Kenya

### Abstract

While food production has steadily risen across the globe over past decades, places such as sub-Saharan Africa (SSA) has witnessed either a decline, or stagnation at best. Rapidly rising urban populations have further challenged food production and supply systems. To sustainably improve productivity and provide more and safer food to both urban and rural areas, crop production systems in SSA need to radically change. A large proportion of farmers are smallholders, often operating in a subsistence manner. Systems therefore need to intensify, which can also exacerbate the already excessive pest and disease losses. Under tropical and sub-tropical conditions nematode pests are a particular, and rising, concern. Farmers, however, are largely unaware of these pests, despite them causing serious damage. A limited presence of nematologists have traditionally undermined efforts to either mitigate nematode problems or raise awareness to them. To change this, substantial investment in training and capacity building is required, in addition to strengthening links with the agro-input industry. This presentation highlights the severe damage that nematodes are causing to crop production in places such as Africa. It provides an overview of options suitable for nematode management under such conditions and the huge potential this could deliver. It also demonstrates the power of collegiate, collective efforts towards raising awareness on nematode pests, strengthening capacity and delivering appropriate management options through NemAfrica, an institutional collaborative initiative to drive nematology research in East Africa.

## Bacterial and eukaryotic microbiomes of Tobrilid nematodes: insights from alkaline lakes of the Nebraska Sandhills

Critchfield, Ricky^1^, P. G. Mullin^2^, T. Harris^2^, K. Powers^2^, T. O. Powers^2^ and D. Porazinska^1^

^1^University of Florida, Department of Entomology and Nematology, Gainesville, FL 32611

^2^Department of Plant Pathology, University of Nebraska, Lincoln, NE 68503

### Abstract

Gut microbiomes significantly affect the fitness and ecology of their nematode hosts. However, most studies have largely focused on nematode models such as *C. elegans*. Members of the Tobrilidae family are well-known omnivores/predators believed to feed on food sources at higher trophic levels such as microbial eukaryotes. Consequently, the eukaryotic microbiome may exhibit its own diversity patterns and roles in nematode ecology. The alkaline lakes in the western Nebraska Sandhills provide a useful model system to examine Tobrilid gut microbiomes. Although Tobrilids predominate nematode communities in all lake sediments, they are the only nematode taxon at the highest alkalinity levels. Gut microbiomes, both bacterial and eukaryotic, may play a role in nematodes’ ability to live under extreme conditions. In October 2023, we collected four sediment samples from each of three lakes varying in alkalinity (pH 8-11) and extracted nematodes using sugar centrifugation and modified Baermann funnels. Sediment samples and individual hand-picked Tobrilid specimens underwent 16S (515F-926R) and 18S (Euk1391f-EukBr) rRNA metabarcoding via Illumina sequencing to characterize bacterial and eukaryotic communities, respectively. Nematode identity was determined using Sanger sequencing with an 18S NF1/18Sr2b primers. Additionally, we characterized lake sediment biogeochemistry to examine its role in shaping bacterial and eukaryotic microbiomes. We used Generalized Linear Models and the post-hoc Tukey’s HSD test to assess differences in alpha diversity among Tobrilid species and lakes. Similarly, we used PERMANOVA to evaluate the differences in microbiome compositions and visualized them with NMDS and Upset plots. Furthermore, we used Random Forest models and dbRDA to determine the importance of sediment microbial richness and biogeochemistry to alpha diversity and composition, respectively. Both bacterial and eukaryotic sediment microbiomes were significantly more diverse than Tobrilid gut microbiomes. Generally, both sediment and Tobrilid bacterial microbiomes were the least diverse in the most alkaline lake. Eukaryotic microbiomes retained this pattern in lake sediments, but in Tobrilids showed greater diversity in the most alkaline lake. Both bacterial and eukaryotic microbiomes of lake sediments clustered away from nematode microbiomes. Based on Random Forest models, the bacterial alpha diversity of Tobrilids was the most influenced by nematode identity, lake, sediment pH, and concentrations of potassium and organic matter, while the eukaryotic alpha diversity was independent of nematode identity and lake and shaped by pH and concentrations of organic matter, magnesium, calcium, potassium, zinc and manganese. Additionally, dbRDA identified the roles of nematode identity/lake interaction, organic matter, and bacterial richness in lake sediment on the composition of Tobrilid bacterial microbiomes. Similarly, the composition of Tobrilid eukaryotic microbiomes was influenced by nematode identity/lake interaction, organic matter, eukaryotic richness in lake sediment, and iron concentration. This study suggests the integral role of both bacterial and eukaryotic microbiomes in shaping nematode ecology, particularly under extreme alkalinity.

## Efficacy of dazomet fumigation on Plant-Parasitic Nematodes during golf green renovation

Crow, William T. and C. M. Green

Entomology and Nematology Department, University of Florida 32611

### Abstract

Periodically, after years or decades, golf courses will renovate putting greens to remove grass mutations, contamination from other grasses, or to switch to a more desirable grass cultivar. In past decades methyl bromide was the primary tool for this purpose, but currently many golf courses have implemented fumigation with dazomet for green renovation. While plant-parasitic nematodes (PPN) are not the primary target for this fumigant use, golf course superintendents are interested in knowing the amount of incidental nematode control achieved during dazomet fumigation. This study used soil from a golf green being fumigated for renovation. Soil was collected from 9 locations in the field immediately prior to fumigation, and post fumigation when the tarp was removed after five days. The soil was collected at three depths, 0–10, 10–20, and 20–30 cm. The soil from each depth was mixed and then placed into cylindrical “pipe-pots”, three pots for each soil depth, and sprigged with PPN-free bermudagrass. The pots were then arranged in a randomized block design in a greenhouse and maintained for 4 months to allow populations of PPNs surviving the fumigation to increase to detectable levels. Then the pots were emptied and PPNs extracted from the soil and from the grass roots, identified to genus, and counted. *Helicotylenchus* sp. was only found in non-fumigated soil indicating no fumigant survival, while two genera of PPNs, *Hoplolaimus* sp., and *Mesocriconema* sp. were detected in both non-fumigated and fumigated soil, indicating survival of the treatment. *Hoplolaimus* was reduced to a single nematode or less by fumigation at all three depths indicating high efficacy. *Mesocriconema* was reduced to a single nematode or less by fumigation at depths of 0–10 and 20–30 cm, however, it was abundant at 10–20-cm deep, with populations in fumigated no different from non-fumigated treatments.

## Distribution and spread of *Globodera pallida* in Idaho

Dandurand, Louise-Marie^1^ and J. B. Contina^2^

^1^875 Perimeter Drive MS 2339, University of Idaho, Moscow, ID 83844-2339

^2^22000 Bend Ferry Rd, Driscoll’s Inc., Red Bluff, CA 96080

### Abstract

The nematode parasite of potato, *Globodera pallida*, is limited in host range to potato and a few other solanaceous crops and is well adapted to survive in soil for many years. A regulated pest worldwide, its discovery in Idaho in 2006 prompted immediate phytosanitary containment and eradication measures. Because *G. pallida* poses a substantial economic risk to the entire Idaho potato industry, it is regulated by USDA-APHIS and ISDA and is the focus of intense containment and eradication efforts. As of 2025, 2,611 acres of Idaho farmland are regulated, of which 1,432 acres are infested with *G. pallida* and 1,179 acres are regulated by association with an infested field. The stringent adherence to phytosanitary measures has contained *G. pallida* to two counties in Idaho within a 13.6-km radius. Infested land remains less than 1% of the total acreage planted to potato in Idaho. A spatial analysis was used to understand the distribution of *G. pallida* in Idaho and to evaluate the potential threat of *G. pallida* entry into new areas. Spatial analysis indicated that *G. pallida* infested fields are spatially aggregated and that 80% of the infested fields are located within a radius of less than 10 kilometers. This result showed that the infested fields, rather than being distributed randomly across the study area, followed an aggregated pattern of distribution. Prior to the establishment of containment measures, the spread of *G. pallida* followed a contagion effect scenario where infested fields contributed to the infestations of nearby fields, probably by agricultural machinery or plant materials contaminated with soil infested with *G. pallida*. However, containment measures disrupted the momentum of *G. pallida* spread. We determined that the presence of *G. pallida* in southern Idaho is unlikely to be associated with new introductions from outside the state of Idaho. On entering the USDA-APHIS regulatory program, fields infested with *G. pallida* had an average of 4,263 cysts/ha and egg viability of 25%. With fumigation, *G. pallida* spread was prevented, and the viability of this invasive nematode has fallen below detection in many fields. Through continued use of soil fumigants, de-regulation of infested fields is in process. The impact of containment and fumigation on the spread of *G. pallida* will be discussed, as well as the progress which has been made to deregulate infested fields.

## Examination of a nematode community from a bermudagrass golf green

Davidson, Wade, C. Green and W. T. Crow

Entomology and Nematology Dept., University of Florida, Gainesville, FL 32611

### Abstract

As part of a six-year study on TifEagle™ bermudagrass, nematode communities were sampled for assessment. The bermudagrass was managed intensively to maintain as a golf green, except for the application of nematicides, and succinate dehydrogenase inhibitor (SDHI) fungicides. Nematode samples consisting of four 3.8-cm-diam, and 6.35-cm-deep cores were collected from five 3.34 m plots. Nematodes were extracted from soil and plant-thatch material separately and then the extracted nematodes from both extractions were combined for each plot. Nematode-based indices were calculated and compared to other studies featuring perennial and agricultural nematode communities. The bermudagrass nematode community indices demonstrated a robust and mature community. Though plant parasitic nematodes were more abundant and diverse than other sites, the abundance and distribution of the different functional groups were well represented and demonstrated by the maturity and structure indexes. The enrichment index measured in the moderate range and structure index was relatively high showing that even with constant and intense mowing, fertilization, and chemical inputs, the bermudagrass was able to maintain a well-functioning, structured, and diverse nematode community.

## Nematode wrangling: managing nematodes as a resource

Davidson, Wade, C. Green, I. Scramoncin and W. T. Crow

Entomology and Nematology Dept., University of Florida, Gainesville, FL 32611

### Abstract

The study of nematodes often requires live cultures of different species of plant-parasitic nematodes (PPN) for research and teaching. However, it is often difficult to grow or obtain enough nematodes of a given species for the desired purposes. Different kinds of plant parasitic nematodes have differing environmental needs, time requirements, host plants, and methodologies for population establishment and maintenance. It is necessar y to maintain healthy host plants to grow nematodes in abundance. Greenhouse management must be a priority when maintaining multiple host plants with different nematode species and isolates. Keeping the plants healthy requires fertilizer and proper pest management. The practical use of integrated pest management involves careful observation and manipulation of a variety of possible variables: temperature, hydration, host plant selection, geographical arrangement, isolation, chemistry rotation, etc. Periodic quality control is required to ensure that the species’ isolates remain pure. The University of Florida Entomology and Nematology Dept.’s Landscape Nematology Lab maintains one of the largest collections of PPNs in the world and provides them to educators and researchers throughout the United States. An overview of their techniques will be presented.

## Comparing the efficacy of fluopyram (Broadform) and spirotetramat (Kontos) to the commercial standard chlorphenapyr (Pylon) on *Aphelenchoides pseudobesseyi* in ornamental plants

Demesyeux, Lynhe, L. Wheeler and W. T. Crow

University of Florida, Dept. of Entomology and Nematology, Gainesville, FL 32608

### Abstract

*Aphelenchoides pseudobesseyi* is a recently described foliar nematode that was separated from the A. besseyi species complex. Affected plants present similar symptoms to other foliar nematodes such as: vein-limited leaf lesions, leaf crinkling, bud and leaf abscission, and reduced plant vigor which decrease the aesthetic value of the plants. Phylogenetic analysis of multiple *Aphelenchoides* populations suggest that this nematode primarily infects soybeans, common beans, and ornamentals plants. *Aphelenchoides* species are general feeders that feed on a large variety of plants. Additionally, the production settings of ornamentals make it ideal for foliar nematodes spread. Considering the lack of research on this new parasite and the threat it represents to the ornamental industry in Florida, this research compared the efficacy of ornamental plant-labeled formulations of fluopyram (Broadform) and spirotetramat (Kontos) to the commercial standard chlorphenapyr (Pylon) on *A. pseudobesseyi* infecting chrysanthemum sp. The experiment was conducted under greenhouse conditions where each product was applied either once, twice, or three times at a two-week intervals. Data on nematode recovery; plant quality and severity of the damage were recorded 2 weeks after the last treatment for each plant of the 11 treatments including the positive and negative controls. All three products were effective at reducing the number of nematodes in the treated plants compared to the untreated control. However, fluopyram and chlorfynapyr applied at least two times and spirotetramat applied at least three times were the most effective (P < 0.05) at suppressing A. pseudobesseyi on chrysanthemum. These results suggest that fluopyram and spirotetramat are good candidates for the management of foliar nematodes on ornamental plants.

## Fluopyram and the new wave of flu(orine) crop nematicides – the Florida experience

Desaeger, Johan, H. Bui, C. Xie and J. Carter

University of Florida, Department of Entomology and Nematology, Gulf Coast Research and Education Center, Wimauma, FL 33598, USA

### Abstract

Ever since their first use in the mid-1900s, crop nematicides have always been among the most toxic and environmentally unfriendly products around (e.g. soil fumigants and organophosphate and carbamate nematicides). It is only since the last two decades that there has been a resurgence of nematicide discovery in the crop protection industry, and a new generation of low-tox fluorine-based (flu) nematicides has become available. The first such products were Nimitz (a.i. fluensulfone) and Velum (a.i. fluopyram), which now have been around for ten years. Last year, Salibro (a.i. fluazaindolizine) also became available to growers in Florida. The new nematicides have different modes of action, are more selective, less toxic, and more user-friendly than previous nematicides. In this presentation, we will give an overview of our experience testing these products in Florida for the past 8 years on crops such as tomatoes, peppers, cucurbits and strawberries. Experiments were done at the University of Florida research farm near Tampa and in growers’ fields. Results indicate that while the new nematicides should not be considered direct replacements for fumigants, they can be effective tools to reduce nematode damage. All products showed good potential to reduce root-knot nematodes in vegetables, while Velum was the most effective against sting nematodes in strawberries. Adoption by growers was slow initially but has increased in recent years as growers are getting more familiar with the new products, and because fumigation costs continue to increase. Especially, for longer season crops like tomato and strawberry, the ability to apply products during the crop season, as is the case for Velum and Salibro, is something that Florida growers value greatly. We expect that grower adoption of these new nematicides will continue to grow, thereby reducing Florida’s growers’ dependency on fumigants, and leading to more integrated and sustainable nematode management programs.

## Ecto- and endoparasite treatment for reduced greenhouse gas emission and improved calf-cow production outcomes

DeVore, Melette^1^, S. Puri^1^, S. Heil^1^, S. Mishra^2^, A. Rodgriguez^2^, T. Burgess^2^ and P. DiGennaro^1^

^1^University of Wisconsin-Madison, Dept. of Plant Pathology, Madison, WI 53706

^2^University of Florida, Dept. of Entomology and Nematology, Gainesville, FL 32611

### Abstract

With the US pledging 50% reduction in greenhouse gas (GHG) emissions by 2030, aggressive mitigation strategies are needed to meet this deadline. Livestock operations are a major contributor to United States agriculture GHG emissions, with cow-calf production accounting for 70% of all GHG emissions in beef production and 15% of total US GHG emissions. These emissions are primarily enteric methane production. However, GHG emission management strategies in feedlot animals do not translate well to pasture-based systems; to promote adoption, mitigation strategies must have an economic benefit for the producer. Reducing stress and increasing weight gain performance can lower GHG emissions in feedlot cattle and other animal commodities. We hypothesize that parasite management is a potential method to reduce cow-calf stress, possibly improving production outcomes and reducing GHG emissions. Various gastrointestinal nematodes (GIN), such as the medium stomach worm, *Ostertagia ostertagi* are prevalent in US beef production, contributing to economic loss resulting in significant calf weight reduction. Unfortunately, there is surprisingly high variability in parasite control adoption among growers. This variability presents a unique opportunity to reduce GHG emissions while improving cow and calf health and providing economic gains for producers. Here, we evaluate cow-calf treatments for gastrointestinal nematodes using metabarcoding to determine to what extent endo-parasite-induced animal stress and weight gain performance loss translates to GHG emissions, and how the nematode community in cow rumen attenuates this effect. We aim to demonstrate that higher adoption of endoparasite control improves cow and calf health and productivity while also lowering GHG emissions by influencing nematode community structure in the rumen.

## Outpaced by nematodes? Rethinking research and collaboration in Brazil

Dias-Arieira, Claudia R

Universidade Estadual de Maringá, Centro de Ciências Agrárias, Departamento de Agronomia. São Cristovão, Brasil

### Abstract

Brazil’s tropical climate and intensive cropping systems create a favorable environment for plant-parasitic nematodes, which significantly impact crop productivity. In regions like the Cerrado, up to 3.5 cropping seasons per year are possible, often involving soybean, maize, cotton, and wheat—all susceptible to nematodes such as *Meloidogyne javanica*, *M. incognita*, *Pratylenchus brachyurus*, *Heterodera glycines*, and *Rotylenchulus reniformis*. Continuous cultivation of susceptible crops, declining organic matter, and intensive land use have led to rising nematode populations and the emergence of previously secondary species like *Helicotylenchus dihystera* and *Scutellonema brachyurus*. A 2022 survey by the Brazilian Society of Nematology, Syngenta, and AgroConsult estimated annual losses exceeding US$11 billion due to nematodes, with soybean alone accounting for US$5 billion. These figures underscore the urgent need for coordinated investment and collaboration in research, extension, and policy. Public investment in Brazil mainly takes the form of fellowships for postgraduate training. While generally available, low stipends discourage retention of young researchers. Competitive funding calls exist at federal and state levels but often fall short of demand or are hindered by bureaucratic hurdles, particularly at the state level. Public–private partnerships have emerged as a crucial support mechanism, enabling more flexible financial execution through university-affiliated foundations. These collaborations primarily fund short-term needs like consumables but rarely support infrastructure or innovation at scale. Moreover, strict regulations designed to ensure accountability can delay research progress, deterring private partners concerned about market agility. Cooperation among researchers remains underdeveloped. Differences in goals, timelines, and expectations, as well as limited information exchange, hinder collaborative projects from translating into tangible outcomes such as publications, technologies, or policy recommendations. Climate change and population growth compound the challenge. Nematodes are adapting quickly, shortening their life cycles, and expanding their geographic range. Meanwhile, Brazil, and the world, must increase food production without further degrading ecosystems. Addressing this dual pressure demands more than scientific innovation; it calls for strategic alignment among policymakers, researchers, industry, and farmers. Brazil’s experience reveals both the scale of the nematode threat and the systemic barriers to managing it effectively. Unlocking the full potential of research and innovation will require targeted investment, streamlined policy frameworks, and a stronger culture of collaboration.

## Feeding habits of Root-Knot Nematode through heavy isotope labeling

DiGennaro, Peter^1^, K. Hughes^1^, A. Schweiner^1^ and S. Mishra^2^

^1^University of Wisconsin-Madison, Dept. of Plant Pathology, Madison, WI 53706

^2^University of Florida, Dept. of Entomology and Nematology, Gainesville, FL 32607

### Abstract

Understanding feeding behaviors of plant parasitic nematodes is crucial to determining potential impacts on crop yield and quality and provides avenues for novel control methods. Here, we demonstrate the potential of proteomic techniques to determine the feeding rate of Root-Knot Nematode (RKN) over different developmental stages and under various abiotic stressors. Using stable isotope 15N, we tracked nitrogen uptake in plant roots and shoots upon nematode infection and compared nematode to plant nitrogen relative abundances of 15N within nematode galls. We went further to investigate the impact of higher atmospheric carbon dioxide between two near isogenic tomato lines upon nematode infection. Increasing levels of atmospheric CO_2_ stimulates growth resulting in more biomass in some plants; however, this can also lower nutrition levels as the ratio of carbon to nitrogen content decreases. Lower nutrient levels can mean more plant mass must be consumed to meet nutrient requirements, increasing parasite impact on crop quality and yield. Understanding how abiotic stressors, like increased CO_2_, and biotic stressors, like RKN, interact is vital to preparing management strategies to mitigate potential decreases in yield and quality under projected climate changes. This approach is a valuable tool to unveil metabolic pathways associated with nematode feeding. Identified pathways are potential targets for control and can inform protein level interactions between RKN and its host.

## Leveraging entomopathogenic nematode genomes to identify and study proteins involved in parasitism

Dillman, Adler D^1^, A. Baniya^1^, P. Azizpor^1^, K. Anesko^1^, A. K. Lima^1^ and E. M. Schwarz^2^

^1^Department of Nematology, University of California, Riverside, 900 University Ave., Riverside, CA 92521

^2^Department of Molecular Biology and Genetics, Cornell University, Ithaca, NY 14853

### Abstract

Entomopathogenic nematodes (EPNs) are increasingly valuable models of comparative nematode biology. Several entomopathogenic nematode (EPN) genomes have been recently sequenced, and these sequences provide a foundation for functional studies of their secreted products and effectors. We discuss one strategy for utilizing these genomes to identify proteins involved in parasitism. We then describe functional studies of *S. carpocapsae* excretory/secretory (ES) products that have been identified and how some of these proteins affect insect and mammalian biology. These proteins have potential value in the biological control of insect pests. Our data suggest that EPNs are a powerful model for genome evolution and understanding parasitic nematode ES.

## Nematode collection in the UC riverside department of Nematology

Dillman, Adler D., E. Dillman, M. Mundo-Ocampo and S. C. Groen

Department of Nematology, University of California, Riverside, 900 University Ave., Riverside, CA 92521

### Abstract

Nematodes are found on all continents of Earth and inhabit a variety of environments, including agricultural fields, forests, deserts, swamps, and aquatic habitats that range from the tropics to the poles. Nematode collections play a crucial role in preserving nematode diversity and making them accessible for studies across various scientific disciplines. UCR’s nematode collection, housed in the Department of Nematology, was established in 1953 by S.A. Sher, focusing on surveys collected in California and the western United States. Since then, additional surveys from around the world have expanded it to include more than 35,000 slides and approximately 25,000 wet samples preserved in fixative solutions. The collection includes type and voucher specimens. This collection is currently being digitized and made available online through Google Sheets (https://docs.google.com/spreadsheets/d/1TdOAib1RO9sQlxxXSpEXEmqaUAhALlvJPXbZHFr-Koo/edit?usp=sharing). Through-focus videos of some specimens have been uploaded to the YouTube channel UCR Nematology, which currently has over 70 videos. We would like to share this information to enhance access and to improve visibility and the utility of this collection and the resource it represents. It is being upgraded to include light and electron microscopy images as well as other information to make it more valuable as a teaching resource.

## Genomic characterization of inbred lines of soybean cyst nematode

Docherty, Lauren^1^, V. Ramasubramanian^1^, C. Hirsch^2^, A. Lorenz^1^ and S. Chen^2,3^

^1^University of Minnesota, Department of Agronomy and Plant Genetics, St. Paul, MN 55108

^2^University of Minnesota, Department of Plant Pathology, St. Paul, MN 55108

^3^University of Minnesota, Southern Research and Outreach Center, Waseca, MN 56093

### Abstract

Soybean cyst nematode (SCN, *Heterodera glycines*) is a major pathogen of soybean in North America, causing an estimated $1.2 billion in annual yield loss. SCN is primarily controlled by growing resistant varieties, but field populations have demonstrated an ability to overcome resistance after years of growing the same source of resistance. Due to difficulties in extracting sufficient high quality DNA, little is known about the genetics of SCN. Understanding how SCN evolves and which genes are responsible for virulence could improve the efficacy of plant resistance. To begin to tackle this problem, 178 inbred lines of SCN were developed and phenotypically and genotypically characterized. These lines were developed from field populations in Minnesota and inbred through single cyst descent for at least 10 generations. The genome of each line was sequenced to an average depth of 40x and aligned to all nine available reference genomes. TN7 was chosen as the reference genome to be used for further analysis, and variants in the inbred lines were identified. Whole genome sequencing data shows that on average, each inbred line is approximately 20% heterozygous, which is the expected proportion given 10 generations of full-sibling mating. After quality filtering, 1.2 million single nucleotide polymorphisms and short insertions or deletions were identified, suggesting significant diversity. However, little population structure exists among the lines. Principal component analysis, admixture analysis, and a phylogenetic tree show small groups of 14 lines and 4 lines separating from the lines. The remaining 160 lines remain clustered together. To better understand inheritance and population structure in SCN populations, genomewide, per chromosome, and sliding window linkage disequilibrium decay rates were calculated. Finally, a genome-wide association study was performed to identify virulence loci. Each inbred line was phenotyped for its virulence on six resistant soybean lines following a standard HG Type test protocol. Seventeen significant variants were identified. Sizes of linkage blocks around the significant variants were estimated and searched for candidate genes. These results are now being used to begin developing a molecular test to characterize virulence in field populations of SCN.

## Context matters: soil ecosystem responses to regenerative and conservation agriculture

Du Preez, Gerhard

Unit for Environmental Sciences and Management, North-West University, Potchefstroom, South Africa

### Abstract

Conservation and regenerative agriculture are widely promoted for enhancing soil health, yet their effectiveness varies markedly across environmental conditions and management regimes. Despite the central role of soil organisms in ecosystem functioning, biological toolsets such as nematode-based indices remain underutilized in soil health assessments. This study draws on data from multiple on-farm investigations in South Africa to explore how nematode-based indices and other biological indicators can reflect changes in soil ecosystems under real-world agricultural conditions, with a particular focus on the influence of environmental and management contexts. Three independent studies were conducted across different climatic regions and edaphic conditions. The first was a longitudinal trial over three summer seasons comparing conservation, conventional, and undisturbed grassland systems. Results showed a temporal shift in the primary soil health drivers, from mainly abiotic properties (e.g., phosphorus availability and soil structure) to also include biotic indicators such as microbial biomass and nematode-based indices (Maturity, Structure, and Channel indices), underscoring the dynamic trajectory of soil health recovery. Notably, crop rotation involving cover crops enhanced food web maturity and promoted fungal-dominated decomposition pathways. The second study compared regenerative, conventional, and pasture systems on two farms in the Eastern Free State. At one site, management practices (e.g., no-tillage, organic cover, and cover crop integration) drove improvements in biological indicators, including higher Maturity Index values. At the second site, however, soil texture and carbon storage capacity were dominant factors, likely overriding the effects of management practices on soil biological responses. The third study, encompassing six ecotopes across three provinces, assessed soil ecosystem status using nematode-based indices, nutrient availability, and carbon pools. Findings revealed that environmental context, particularly soil physico-chemical properties, significantly shaped biological outcomes, often more so than the management system alone. Together, these studies demonstrate that while conservation and regenerative practices can improve soil ecosystem functioning, the magnitude and trajectory of these improvements are highly context-dependent. Nematode-based indices provided sensitive, functionally relevant metrics of soil ecological status and should be integrated alongside physical, chemical, and other biological measures in comprehensive soil health frameworks. Moreover, the findings highlight the importance of tailoring soil health interventions to local conditions and support the broader adoption of bioindication tools, particularly nematode-based indices, for monitoring and managing agroecosystem resilience across variable landscapes.

## What are the barriers to the development, registration, and adoption of biological control products?

Ehlers, Ralf-Udo

Biocontrol Biotech Consult, Duesternbrook 1, 24211 Rastorf, Germany

### Abstract

Despite political calls to reduce the use of synthetic plant protection products (PPPs), their use is steadily increasing. One reason for this is the development of resistance, which leads to the use of higher doses at shorter intervals. At the same time, the external costs associated with the use of chemical PPPs (medical treatment, remediation of environmental contamination and treatment of contaminated groundwater) are increasing. As a result, we are facing a growing ban on synthetic PPPs, less innovative products are entering the market and, due to high development costs, a negative return on investment precludes the introduction of more selective PPPs. Farmers’ toolboxes are getting smaller. Biocontrol can fill gaps, but the continuous use of pesticides, which do not control the pest but reduce antagonist populations, is a major barrier to the adaptation of biological PPPs. The golden age of chemical pesticides is over, and in the near future we will rely more and more on biocontrol, but even more on conservation biocontrol, which has a huge antagonistic potential if not limited by synthetic PPPs. Biocontrol agents (BCAs) are safe, sustainable, produce no external costs and preserve natural antagonistic potential. But can the industry provide enough? Modern biotechnology has been able to bring production technology to an industrial scale, allowing for economies of scale. In addition, modern application technology has been able to better target the BCAs, reducing application density and further lowering costs. Genetic marker-based selection and breeding programs of *Heterorhaditidis* spp. are providing improved strains, allowing further reductions in application density. Entomopathogenic nematodes are generally exempt from the registration requirements that apply to microbial agents, but the introduction of the Nagoya Protocol and the exclusion of exotic species have greatly increased the bureaucratic hurdles, with a particularly strong negative impact on classical biocontrol. Decisions on the release of exotics must be based on a comprehensive analysis of the relevant risks and benefits and not be influenced by xenophobia. Biocontrol agents, including microbial PPPs, have a long history of safe use. Therefore, excessive regulatory requirements cannot be justified. For rapid market access of MBCAs, new regulatory regimes need to be developed that are adapted to their real risks and include microbes for use as biostimulants, biocides, feed additives and fertilizers. GRAS or QPS approaches are possible. Authorization should be granted without time limit and unlimited label (application in all crops). Fast track and provisional approval must be implemented for low-risk BCAs. Proportional fees must stop subsidizing the registration of chemical PPPs. Biocontrol can continue to support the transition of agricultural practices if registration requirements are adapted and exit strategies from chemical pesticides are implemented.

## Cost effective control of corn rootworm larvae (*Diabrotica v. Virgifera*) with *Heterorhabditis Bacteriophora*

Ehlers, Ralf-Udo

Biocontrol Biotech Consult, Duesternbrook 1, 24211 Rastorf, Germany

### Abstract

The invasive pest western corn rootworm (*Diabrotica virgifera virgifera*) is a major pest of corn. Since the ban on neonicotinoid seed treatments, most European growers have relied on less effective granular soil insecticides. A sustainable, non-toxic alternative is a genetically improved entomopathogenic nematode hybrid strain of *Heterorhabditis bacteriophora*. This nematode has been successfully tested for a decade in plot and large-scale field trials in Austria and Hungary. Conventional application technology of nematodes applies 2.5×10^9^ ha^−1^ with minimum 5000 l water ha^−1^. This is technically and economically not feasible. As a first step to overcome the limitation of too high water application volumes, nematodes were applied with the seed in the furrow in March/April with an easily adaptable application technology. The control was comparable to that achieved by spraying at L2 pest occurrence in June. As the product cost was still higher than granular pyrethroid application, we tried to reduce the application density. When applied at seeding, nematodes must persist for 4–6 weeks until the insects hatch from their eggs. To prolong nematode soil persistence and increase virulence against rootworm larvae, a collection of lines obtained from wild type strains from all over the world was phenotyped and genotyped and genetic SNP markers were identified. Hybridization of inbred lines with improved traits resulted in a strain combining both traits. With this strain it is possible to reduce the application rate to 1 × 10^9^ ha^−1^ with 200 ltr ha^−1^ without loss of control. Using this precise liquid application method on the seeds of a genetically enhanced strain, the use of *H. bacteriophora* achieved an average reduction in pest population of 69%, exceeding the results achieved with chemical standards. Reduced application density, combined with low-cost industrial-scale liquid culture production, makes this hybrid the first macrobial product in the biocontrol market based on a strain derived from a molecular marker-assisted breeding program. This commercial development has resulted in a biocontrol application offered at a cost competitive with chemical insecticides.

## Nematodes can move without a water film

Ehlers, Ralf-Udo

Biocontrol Biotech Consult, Duesternbrook 1, 24211 Rastorf, Germany

### Abstract

All textbooks tell us that nematodes need a film of water to move in the soil. This presentation will show that this is a false conclusion from experiments conducted in the 1960s. The movement of nematodes in the soil depends on their biology, mobility, tolerance to environmental factors (soil moisture, temperature), edaphic parameters and soil water dynamics. The free-living stage of EPNs, for example, is the 3rd instar, the so-called dauer larva (DL). Due to its diameter of 25–43 µm (depending on the species), it can only move through coarse soil pores (defined as > 10 µm diameter). Considering that the nematode can only move when these pores are filled with water, movement would be impossible once these pores are dry. These pores are dry at a water potential (pF) < 2.5 (corresponding to −300 hPa). If DJs could only move on a water film, EPN infestation or damage by PPN would be impossible when the coarse pores are dry. However, results from field trials show that EPN control is achieved at lower values and damage by PPN as well. The presentation will explain why the assumptions based on previous experiments are wrong, based on a misinterpretation of observations of nematode movement in soil particle monolayers. Up to the permanent wilting point of plants (pF = 4.2, corresponding to −10^4.2^ hPa), the relative humidity in the coarse pores is above 98%, enough for nematodes to move. This conclusion also applies to nematodes such as PPN. Otherwise, we would not record nematode damage when coarse pores are dry at a pF between 2.5 to 4.2.

## Effect of water carrier volume on fluopyram efficacy for nematode management in row crops

Faske, Travis, M. Emerson and B. Baker

Department of Entomology and Plant Pathology, Lonoke Extension Center, University of Arkansas System, Division of Agriculture, Lonoke, AR 72086

### Abstract

Fluopyram is marketed as a liquid, soil-applied (i.e., in-furrow) nematicide to manage plant-parasitic nematodes in corn, cotton, and soybean. Currently, there is no information on whether using a higher water carrier volume with fluopyram improves the management of southern root-knot nematode, *Meloidogyne incognita*, and other plant-parasitic nematodes, or enhances yield protection in these three row crops. In the study, five susceptible corn hybrids, two susceptible and one resistant cotton cultivar, and six susceptible soybean cultivars were used in three field experiments. One rate of fluopyram was used per crop, and experiments were conducted twice (2023 and 2024). Fluopyram was applied at a standard carrier volume (5.0 to 6.5 gal/ac or 47 to 61 L/ha) and a higher carrier volume (60% more). In corn, the suppression of mid-season nematode densities and protection of grain yield by fluopyram were similar among carrier volumes and the nontreated control. In cotton, the percentage of root system galled at mid-season and the protection of lint yield by fluopyram were similar among carrier volumes and the nontreated control. In soybean, a lower (*P* = 0.10) percentage of root system galled (upper 10-cm) by fluopyram was observed for both carrier volumes compared to the nontreated control. Further, a greater (*P* = 0.10) grain yield was observed for the higher carrier volume than the standard volume and the nontreated control. Increasing the water carrier volume may be beneficial for some but not all row crops to suppress nematode infection and protect grain or lint yield.

## Host status and damage response of two cacao cultivars to Plant-Parasitic Nematodes in Hawai‘i

Faaulu, Arieta and B. Sipes

University of Hawai‘i at Mānoa, Dept. of Plant and Environmental Protection Sciences, 3190 Maile Way, Honolulu, Hawai‘i, 96822

### Abstract

Plant-parasitic nematodes could threaten the viability of Hawai‘i’s emerging cacao industry by compromising root health, reducing plant growth, and limiting bean yield. However, the host status and damage potential of widespread plant-parasitic nematodes on locally grown cacao cultivars is undocumented. This study evaluated the susceptibility and damage response of *Theobroma cacao* cultivars ICS-95, a red pod, and HSCT-4, a yellow pod, to *Meloidogyne incognita, M. javanica,* and *Rotylenchulus reniformis*. Seeds were germinated, seedlings transplanted, inoculated with 2000 nematode eggs/plant, and grown in the greenhouse. Three months after inoculation, shoots were cut at the soil line and biomass measured. Roots were gently washed in water to remove soil and then shaken in a 5% NaOCl solution to collect eggs which were then counted. A nematode reproduction factor (Rf = final number of eggs/inoculated number of eggs) was calculated. A factorial ANOVA tested the effects of cultivar, nematode species, and their interaction on plant growth and nematode reproduction. A Tukey’s HSD for post-hoc comparisons was conducted when appropriate. Both cultivars supported reproduction of all nematode species (Rfs > 1), confirming susceptibility. *M. javanica* reproduced the most, Rf = 8.69, but *R. reniformis* caused the greatest biomass reduction, 24% less than the uninoculated control plants. ICS-95 consistently suffered greater biomass loss across nematode species, indicating higher vulnerability compared to HSCT-4. Notably, nematode-infected plants often maintained root and shoot biomass similar to controls despite high nematode reproduction, suggesting potential tolerance traits worth further investigation. HSCT-4’s potential as a rootstock has been noted in the literature. These findings provide critical baseline data for cacao growers in Hawai‘i and other tropical regions, guiding cultivar selection and nematode management decisions. Understanding cultivar-specific responses to nematode pressure can improve root health, sustain yield, and enhance the long-term viability of cacao production in Hawai‘i’s unique agroecosystems.

## Novel nematode associate of the Mountain Pine Beetle

Furlonger, David Douglas^1,2^, K. Bleiker^2^, Q. Yu^3^, J. B. Tanney^2^ and S. J. Perlman^1^

^1^University of Victoria, Department of Forest Biology, Victoria, BC, Canada

^2^Pacific Forestry Centre, Canadian Forest Service, Natural Resources Canada, Victoria, BC, Canada

^3^Agriculture and Agri-Food Canada, Ottawa Research and Development Centre, Ottawa ON, Canada

### Abstract

Mountain Pine Beetles (MPB) (*Dendroctonus ponderosae*) are important forest pests, with a recent MPB outbreak killing half of mature lodgepole pine trees across the province of British Columbia, Canada. Many nematodes are known to be associated with the MPB, including nine species that are found on the beetle’s external surface, and four that are endoparasites. In this presentation I will discuss a new species of *Bursaphelenchus* that we found associated with mountain pine beetle populations in western Canada. This nematode is distinct in its external position underneath the jugal fold of the MPB hind wing where it assumes a dauer state in small clusters without any obvious structures, such as nematangia. Frequency of association and ecological factors such as diet, dispersal and impacts will be discussed, as well as future research directions.

## Reaching 126 million small family farms in India with sustainable integrated nematode management options, current research status and future needs

Gaur, Hari S.^1^ and R. A. Sikora^2^

^1^School of Agriculture, Galgotias University, Greater Noida-203201, India

^2^INRES, University of Bonn, Bonn-53127, Germany

### Abstract

The nematode fauna of India includes vast diversity of nematodes in 15 broad agroclimatic zones and 72 sub-zones of varied temperatures, water, soil types and many crops being cultivated over many centuries. Multivoltine phytoparasitic nematodes cause more damage in the subtropics. Major focus now is on the root-knot, cyst, burrowing, citrus and white-tip nematodes. The root-lesion, reniform, rice-root and several ectoparasitic nematodes are also prevalent. At high population densities, these nematodes too cause economic losses. Increasing menace of root-knot nematodes alone and in complexes damaging guava, pomegranate, grapevines, vegetables and rice, etc. in open fields and in polyhouses, has attracted the attention of the growers and government. Effects of climate change, increasing temperature and decreasing water and soil-organic matter content further accentuate the biotic stress on plants due to nematodes. Small farmers have few specific or spectacularly effective feasible options. Some approaches adopted are: crop rotation, summer ploughing, soil solarisation, adjusting planting dates or seasons, and removal of infected roots and debris. Hot water treatments are recommended for tubers, rhizomes and paddy seed. Organic amendment with compost, green manures, deoiled seed cakes, biofumigation etc. are helpful. However, their availability in sufficient quantities, and their increasing price are constraints. Several products have been developed with bacteria like *Pseudomonas fluorescens* and *Bacillus subtilis*, and fungi such as *Purpureocillium lilacinum, Trichoderma harzianum, T. viride, Pochonia chlamydosporium,* etc. Techniques have been developed for their on-farm multiplication and in cottage industry. Resistant varieties are available in only a few crops. A few nematicides like Fluopyram, Fensulfothion, Metham sodium, etc., are now recommended but used by very few due to their high costs. Prevention of entry and spread, monitoring and integrated approach involving long-term advance planning are advised but difficult to implement effectively due to the prevailing multi-season, multiple and mixed cropping practised by over 126,000,000 marginal farmers holding <2 ha. Cyst nematodes are relatively easier managed using alternative non-host crops. Disinfection protocols have been developed for seed potato tubers. Most available nematode management options fit in well in the sustainable, organic and conservation agriculture systems. About 20 nematology centres in ICAR Institutes and State Agricultural Universities conduct coordinated research, teaching and extension activities in the different ecologies. Several sources of resistance have been identified but rarely used in breeding. Research on nematode resistance using marker-assisted breeding and gene editing by CRSPR-Cas-9 etc. have been taken up at a couple of centres, research funds getting scarce. 731 KVKs help in extension, but have very few nematologists. The literary and technical illiteracy of many older small farmers, numerous languages and dialects, their poor economic conditions and remote locations make nematology extension a huge challenge. Development of good text, photo and video content, novel multi-lingual translation systems, satellites, Apps and AI applications will help reach the small, marginal, as well as the large farmers in remote rural areas better.

## *Nematodes escaped!* Responding to invasive Plant-Parasitic Nematodes

Gorny, Adrienne

Department of Entomology and Plant Pathology, North Carolina State University, Raleigh, NC 27695

### Abstract

*Life, uh, finds a way.* This ominous line from the 1993 blockbuster movie *Jurassic Park* foreshadowed the escape of ferocious dinosaurs, brought to life after eons of extinction. Although magnitudes smaller (but equally as destructive), some plant-parasitic nematodes have also escaped, becoming invasive species. Meeting the challenge of invasive phytonematodes requires clear management goals, a multi-faceted response, coordinated efforts among individuals and organizations, and practical, but impactful policies. An example of one such challenge is *Meloidogyne enterolobii*, an invasive and virulent root-knot species in North Carolina and the Southeastern US. This species has been particularly impactful to the North Carolina sweetpotato industry, where cosmetic damage due to root-knot nematode galling renders infected sweetpotato roots unmarketable. After initially confirming its presence in North Carolina in 2011, an early goal was to keep the nematode from spreading to neighboring states through sweetpotato seed roots and planting material, which was operationalized through an internal quarantine. Initial efforts coordinated between the NC Department of Agriculture, NC Cooperative Extension agents, university researchers, and commodity board leaders focused on stakeholder awareness, educating producers in the state about *M. enterolobii*, the symptoms to look for in sweetpotato and other crops, and best practices for limiting spread on individual farms. This was carried out through multimedia programs, including in-person training, Extension presentations, videos, websites, and printed materials. Inspection services and diagnostic labs providing molecular speciation have remained critical to identifying *M. enterolobii* amongst other endemic root-knot nematode species. Scientists at North Carolina State University along with collaborators throughout the southeast have responded to *M. enterolobii* with projects in basic and applied research, thus supporting short-, medium- and long-term management, including chemical nematicides, cultural tactics, and host resistance. Listening to input from stakeholders and demonstrating the impact of certain management tactics has promoted adoption and implementation of the most effective research outcomes. *Meloidogyne enterolobii* and other invasive plant-parasitic nematodes still pose a formidable challenge to agriculture and ecosystems. Yet nematologists can flip the script. We can *find a way* to tame invasive plant-parasitic nematodes, support robust biosecurity measures, and build resiliency into our agricultural systems. No electric fence required.

## Are pin nematodes involved in the emerging chickpea health issue in Saskatchewan?

Gouvea Pereira, Fernanda^1^, M. Tenuta^1^, M. Hubbard^2^ and S. Anderson^3^

^1^University of Manitoba, Dept. of Soil Science, Winnipeg, Manitoba, Canada

^2^Agriculture and Agri-Food Canada, Swift Current, Saskatchewan, Canada

^3^Saskatchewan Pulse Growers, Saskatoon, Saskatchewan, Canada

### Abstract

Chickpea crops in southern Saskatchewan have been facing health problems characterized by symptoms such as chlorosis, wilting, and plant die-off. First noticed in 2019, this issue has affected a broad area, including the main chickpea-growing region in the province. Crop specialists conducted field soil surveys in symptomatic and asymptomatic locations; the University of Manitoba analyzed the resulting 143 soil samples for the presence of plant parasitic nematodes. The pin nematode (*Paratylenchus spp.*) was recovered at high densities and fairly frequently from samples. To investigate if chickpea is a host, we conducted a growth chamber study utilizing soil samples with high *Paratylenchus* density collected from the 2022 survey. Three treatment groups were used: soil with *Paratylenchus* and chickpea plants present (CDC-Corrine, 17 replicates); soil with *Paratylenchus* without plants (4 replicates); and chickpea in *Paratylenchus-*free soil (3 replicates). Soil had an initial *Paratylenchus* population of 502 per 100g of dry soil. After 16 weeks, nematodes from soil and roots were isolated using the sieving-sugar flotation method. *Paratylenchus* and other prominent nematodes were identified to genus by morphological features and to species by sequencing of the partial 18S, 28S (D2-D3), and ITS (ITS 1 & ITS2) regions of the rDNA gene. Sequencing showed the species of *Paratylenchus* to be *P. projectus*. At the end of the experiment, chickpea soil and roots had a mean of 5,518 *Paratylenchus* per 100g^−1^ dry soil (±1180 s.e.), with a reproduction factor of 10.9 (±2.35 s.e.), highlighting chickpeas as an excellent host. Without chickpea, pin nematode population declined by 56%. However, the growth chamber-grown plants did not show disease symptoms. This study confirms that *Paratylenchus projectus*, recovered from Saskatchewan, is a parasite of the tested chickpea variety. Further growth chamber studies are underway to screen the most popular chickpea varieties as potential hosts for *Paratylenchus*. Future field studies are being planned to investigate the effects of *Paratylenchus* on chickpea health, if other crops are hosts, and to understand its overall impact on the chickpea health issue in Saskatchewan.

## NEM-EMERGE: A collaborative research and innovation project to counter the emergence and proliferation of invasive and virulent nematodes in Europe and beyond

Goverse, Aska

Wageningen University & Research, Laboratory of Nematology, Droevendaalsesteeg 1, 6708PB, Wageningen, the Netherlands

### Abstract

Soil-borne plant-parasitic nematodes are a biosecurity risk for global food production with an estimated annual loss of €110 billion worldwide. Root-knot nematodes (RKN) and potato cyst nematodes (PCN) rank 1 and 2 in the Top 10 of high-impact plant-parasitic nematodes with RKN alone accounting for ~5% of global crop losses. RKN and PCN are A2 quarantine pests or emerging species listed on the EPPO Alert List. The two PCN species are also included in EU Commission implementing regulation 2021/2285. Recent reports document the emergence of new RKN and PCN problems in tomato and potato cropping across Europe and beyond due to two independent drivers: global warming and genetic selection. For decades, non-specific, environmentally harmful agrochemicals have been applied to manage RKN and PCN. The increasing awareness about their negative impact prompted the phasing out of most nematicides. Consequently, there is an urgent need for novel, durable control strategies that enable adequate responses by stakeholders to prevent crop losses in the EU and beyond. NEM-EMERGE aims to provide a spectrum of sustainable, science-based solutions for both the conventional and organic farming sector based on the principles of IPM, including (1) optimized crop rotations schemes including cover crops, (2) tailored host plant resistances, and (3) optimal use of the native antagonistic potential of soils. Moreover, monitoring and risk assessment tools will be generated to support Plant Health Authorities in decision and policy making. To ensure the adoption and implementation of NEM-EMERGE tools in the sector, a bottom-up co-creation process and multi-actor approach is used based on stakeholder demands. This makes NEM-EMERGE one of the key drivers for the transition to sustainable farming in line with the Horizon EUROPE work program and the Farm to Fork Strategy, thereby contributing to the challenging targets set by the Green Deal of the EU Commission.

## Comparative efficacy of SDHI nematicides against *Hoplolaimus galeatus* in golf turf

Guri, Erica and W. T. Crow

Entomology and Nematology Dept., University of Florida, Gainesville, FL 32611

### Abstract

The golf industry significantly contributes to the U.S. economy, generating $101.1 billion in 2022 and influencing other sectors with an additional $120 billion in revenue. However, lance nematode (*Hoplolaimus galeatus*) poses a substantial threat to golf turf, necessitating effective management strategies. This study aimed to evaluate the efficacy of fluopyram (Indemnify®, Envu, Cary, NC) and a new active ingredient, cyclobutrifluram (Tymirium Technology™, Syngenta Crop Protection, Greensboro, NC), in suppressing lance nematodes on bermudagrass. Nematode populations were collected and multiplied on bermudagrass in a greenhouse. Two trials, a bioassay and a greenhouse experiment, were conducted. The bioassay involved exposing mixed life stages of lance nematodes to fluopyram and cyclobutrifluram at concentrations of 0, 10, and 50 ppm, with observations at 24 and 72 hours. An NaOH solution was used to stimulate nematode movement, and percent mortality was computed. In a greenhouse experiment, mixed life stages of lance nematodes were inoculated onto bermudagrass in pipe pots and received drench treatments of fluopyram (500g/ha) and cyclobutrifluram (500g/ha, 250g/ha and 125g/ha) at 4-week intervals for four applications. Results from the bioassay indicated that cyclobutrifluram resulted in higher nematode mortality compared to fluopyram. The greenhouse experiment demonstrated that cyclobutrifluram suppressed nematode density in 100 cm^3^ soil, whereas fluopyram did not. These findings suggest that cyclobutrifluram exhibits strong efficacy against *H. galeatus* and could serve as a promising nematicidal option for golf turf management.

## Assessment of ATP levels in Plant-Parasitic Nematodes on Florida golf turf using a bioluminescence ATP assay

Guri, Erica^1^, W. T. Crow^1^, T. E. Burgess^1^ and S. Mishra^2^

^1^Entomology and Nematology Dept., University of Florida, Gainesville, FL 32611

### Abstract

Plant-parasitic nematodes such as *Meloidogyne graminis* and *Belonolaimus longicaudatus* are major pests on Florida golf courses, causing significant damage through their feeding activities. With limited management options available, golf course superintendents have long relied on nematicides like fluopyram, which has delivered effective control in the past. However, a noticeable decline in fluopyram’s efficacy has raised concerns about the possible development of resistance in some nematode populations. Assessing nematode viability accurately is crucial for evaluating nematicide performance and understanding resistance mechanisms. While mortality bioassays and greenhouse experiments are commonly used, they are often time-consuming and subject to interpretation. ATP, a well-established marker for cellular viability, has yet to be applied in assessing viability in plant-parasitic nematodes. In this study, we introduced a bioluminescence-based ATP assay to measure ATP levels in some populations of *Meloidogyne graminis* and *Belonolaimus longicaudatus* with long history of fluopyram exposure as a direct indicator of viability. This method uses the luciferin-luciferase reaction, where ATP from living nematodes produces light detected by a luminometer. We optimized assay conditions for *M. graminis* and *B. longicaudatus*, focusing on nematode concentration, reagent volumes, and incubation times to ensure reliable and reproducible results. After exposing nematodes to sublethal doses of fluopyram, ATP levels were measured using the ATP Bioluminescence Assay Kit CLS II (Roche, Mannheim, Germany). The bioluminescence ATP assay offers a rapid, sensitive, and objective method for assessing nematode viability. Its application in resistance detection and nematicide efficacy testing presents a valuable tool for improving sustainable nematode management practices on golf turf.

## Toxic alliance: harnessing bacterial symbionts of entomopathogenic nematodes for Plant-Parasitic Nematode biocontrol

Hajihassani, Abolfazl, J. Larkin, R. Kassam and D. Gitonga

Department of Entomology and Nematology, Fort Lauderdale Research and Education Center, University of Florida, Davie, FL, 33314

### Abstract

Entomopathogenic nematodes (EPNs), primarily from the genera *Heterorhabditis* and *Steinernema*, form symbiotic associations with the bacteria *Photorhabdus* and *Xenorhabdus*, respectively. While their isolated symbiotic bacteria have demonstrated high efficacy against plant-parasitic nematodes (PPNs) by inhibiting penetration, development, or reproduction, using EPN alone has yielded inconsistent results in PPN control. Specific species and strains of EPN symbiotic bacteria produce metabolites and/or toxins that exhibit nematicidal or nematistatic effects on various life stages of PPNs in both laboratory bioassays and plant-based settings. However, their efficacy may be reduced under field conditions. Our bioassays showed that crude extracts of *X. griffinae* strain 1050 and *X. poinarii* strain 733 induced high mortality (>95%) in second-stage juveniles of grass root-knot nematodes (*Meloidogyne graminis*) at varying concentrations and exposure times. When exposed to the same bacterial strains, sting nematode (*Belonolaimus longicaudatus*) mortality rates decreased with reduced concentrations. This indicates that different PPN species may exhibit varying responses to EPN symbiotic bacteria, likely due to their body size or structural variations. Further pot experiments on the efficacy of EPN symbiotic bacteria demonstrated that broth culture of *X. bovienii* and *X. szentirmaii* reduced root gall severity and egg production of southern root-knot nematode *M. incognita* while enhancing growth in cabbage. The efficacy of these bacteria exceeded that of commercial products containing *Paecilomyces lilacinus* strain 251 and *Burkholderia rinojensis* strain A396. In field studies with zucchini grown on plastic beds, we found that drip application of *X. bovienii* culture broth failed to reduce root-knot nematode populations and root gall severity compared to the untreated control. These findings highlight the potential of EPN bacterial metabolites for managing PPNs. However, further research is needed to refine application protocols and assess the control efficacy of promising bacterial strains in various cropping systems.

## Overwinter survival of soybean cyst nematode (*Heterodera glycines*) in pennycress and soil under cold conditions

Heydari, Fariba^1^, C. Hoerning^2^, J. X. Zhang^2^, J. A. Anderson^2^, M. Hunter^2^, D. Wyse^2^ and S. Chen^1^

^1^Unversity of Minnesota, Southern Research and Outreach Center, Waseca, MN 56093

^2^University of Minnesota, Department of Agronomy and Plant Genetics, St. Paul, MN 55108

### Abstract

The soybean cyst nematode (SCN, *Heterodera glycines*) is a major pest of soybean (*Glycine max*) production in Minnesota and other regions worldwide. Understanding its biology, including its overwinter survival, is critical for developing effective management strategies. While SCN eggs in cysts and soil are known to tolerate cold conditions, the specific developmental stages capable of surviving winter in North America remain unclear. Pennycress (*Thlaspi arvense*), a winter annual weed currently being domesticated as a potential winter oilseed crop, is a moderate host of SCN, providing an opportunity to study SCN overwinter survival within host roots. In this study, pennycress plants grown in cone-tainers were inoculated with SCN second-stage juveniles (J2) and maintained in a growth room for 2, 5, 9, or 14 days to establish varying developmental stages in roots. Infected plants in cone-tainers were then moved to field for overwintering. Additionally, J2 overwinter survival in soil in cone-tainers maintained in field was assessed. SCN viability was evaluated based on cyst formation and developmental stages in roots after extended pennycress growth or subsequent soybean cultivation in soil containing overwintered J2. Results indicated that SCN J2 could survive in soil during winter field conditions. However, later developmental stages (J2-J4) in pennycress roots did not survive. The pennycress plants maintained in the growth room for 14 days prior to transferring to the field failed to survive after moving back to the growth room, precluding assessment of post-J4 SCN survival in the pennycress roots. These findings suggest that winter pennycress may act as a trap crop by disrupting the SCN life cycle, offering a potential tool for integrated nematode management.

## Using DSSAT to create predictive models of yield impact of the pale cyst nematode, *Globodera pallida* on susceptible and resistant potato varieties

Hickman, Paige and L. M. Dandurand

University of Idaho, Dept. of Entomology, Plant Pathology, and Nematology, Moscow, ID 83844

### Abstract

*Globodera pallida*, the pale cyst nematode is a regulated quarantine pest of potato in Idaho. As such, it is important to understand and predict potential impact of *G. pallida* in Idaho for stakeholders. To determine the effect of *G. pallida* on potato, resistant and susceptible varieties were infested at initial population densities 0, 10, 20, 40, and 80 eggs/g soil in the greenhouse. Due to the quarantine status of *G. pallida* in Idaho, these experiments could not be conducted in the field. Instead, this study incorporated the greenhouse data into the Decision Support System for Agrotechnology Transfer (DSSAT) models to simulate the effect of *G. pallida* on potato under Idaho field conditions. DSSAT is software that can be used to create predictive models for many agricultural crops in specific regions through the integration of weather data, soil data, crop management parameters, and experimental data on crop growth. Within DSSAT, The Simulation of Underground Bulking Storage Organs (SUBSTOR) is a validated potato crop model. Greenhouse data on potato yield and biomass at varying population densities of *G. pallida* was used to calculate yield coefficients. These yield coefficients were integrated into SUBSTOR-DSSAT models of potato yield to predict the impact of *G. pallida* infestations in Idaho field conditions. Models were created for resistant potato ‘Innovator’ and susceptible potatoes ‘Désirée’ and ‘Russet Burbank’. ‘Désirée’ was highly susceptible with prolific *G. pallida* reproduction which resulted in significant yield losses in the greenhouse. ‘Innovator’, a highly resistance European variety, had limited *G. pallida* reproduction but was found to be intolerant and thus still experienced significant yield loss. The SUBSTOR-DSSAT models for ‘Innovator’ and ‘Désirée’ predicted 50% yield loss at an initial population density of 80 eggs/g soil. ‘Russet Burbank’ was also highly susceptible and allowed for greater *G. pallida* reproduction than ‘Désirée’. Greenhouse studies suggested that ‘Russet Burbank’ was tolerant of *G. pallida* as there was not significant reduction in overall yield at any initial population density. However, SUBSTOR-DSSAT models predicted about 10 to 20% yield loss of ‘Russet Burbank’ at high *G. pallida* infestations. Additionally, there was some evidence that ‘Russet Burbank’ yield quality was impacted at high *G. pallida* infestation levels through reduced tuber size and increased numbers of tubers. SUBSTOR-DSSAT is limited in that it cannot create models of average tuber size. Ultimately, the models created by this study help illustrate the effects of *G. pallida* on potato in Idaho and the importance of controlling this pest. This study also highlights another application of DSSAT as a useful tool for modeling pest impact on a major crop.

## The relationship between nematode indicators and soil carbon pools across managed and unmanaged landscapes in California

Hodson, Kathy^1^, K. Marshall^2^, A. C. M, Gaudin^2^, T. Bowles^3^ and K. Jarvis-Shean^3^

^1^University of California Davis, Dept. of Entomology and Nematology, Davis, CA 95616

^2^University of California Davis, Dept. of Plant Sciences, Davis, CA 95616, University of California Berkeley, Dept. of Environmental Science, Policy and Management, Berkeley, CA 94720

^3^University of California Cooperative Extension, Woodland, CA 95695

### Abstract

What can nematode indicators tell us about soil carbon? Improving soil organic carbon as part of a soil health management strategy can increase food available for microbes such as bacteria and fungi. These microorganisms stabilize organic material by binding it together with their exudates and necromass into minerally associated organic matter, which is thought to be more persistent and stable. Bacterial and fungal-feeding nematodes may also respond indirectly to carbon pools or increase microbial stabilization by increasing the rates of microbial turnover through grazing on their microbial prey. The interactions between carbon pools can be complex, however; and there is still much debate about the best way to manage soil carbon stocks as well as the potential roles of soil fauna. In California, across a range of surveys and experiments, nematode indicators related to carbon pools in both managed and unmanaged perennial landscapes. Bacterial-feeding nematodes and their associated indicators (such as the Channel index, Enrichment index and Bacterial metabolic footprint) were most likely to relate to labile forms of soil organic matter. For example, in a riparian woodland, the Channel index decreased, indicating greater relative abundance of bacterial feeders involved in resource processing, while microbial biomass carbon and permanganate oxidizable carbon increased. While these effects were likely due to litter chemistry, similar relationships were found in experiments from multiple agricultural systems such as orchards and vineyards. In a recent survey of almond orchards incorporating different management practices, orchards that “stacked” multiple regenerative agricultural practices and integrated animal grazing into their systems had higher nematode Bacterial and Fungal metabolic footprints than those that maintained bare soil, and only minimally applied soil health principles. These orchards also had higher Nematode predator footprints as well as larger pools of soil organic carbon, microbial biomass carbon and permanganate oxidizable carbon. Alone and taken together, these studies suggest that soil inputs, either through litter deposition or management, can increase soil organic matter pools, which provide food for microbes and bacterial and fungal feeding nematodes, with effects eventually cascading up to predatory nematodes.

## Impact of winter oilseed crops pennycress and camelina on soybean cyst nematode population in Minnesota soybean-corn field rotations

Hoerning, Cody^1,2^, S. Chen^2^, S. Wells^1^ and D. Wyse^1^

^1^University of Minnesota, Department of Agronomy and Plant Genetics, St. Paul, MN 55108

^2^University of Minnesota, Southern Research and Outreach Center, Waseca, MN 56093

### Abstract

Pennycress (*Thlaspi arvense*) and camelina (*Camelina sativa*) are winter oilseed crops that can be implemented in cropping systems of the U.S. Midwest. Incorporating winter oilseed crops into the cropping system offers ecosystem and productivity benefits when the ground is otherwise fallow. However, adding a new crop into an established cropping system may increase pest or pathogen risk. Pennycress and camelina have been identified as a host and poor/non-host, respectively, of the soybean cyst nematode (SCN, *Heterodera glycines*), a devastating soybean pathogen. The objective of this experiment was to investigate whether adding winter pennycress or camelina to a soybean-corn rotation affected SCN population density. The experiment was a two-level factorial with a split-plot design which included SCN susceptible and resistant soybean cultivars as main plots and oilseed crops (pennycress, camelina, and fallow) as subplots conducted at three field sites in Minnesota. Throughout the study, the SCN-susceptible soybean cultivar treatment significantly increased SCN population density when compared to the SCN-resistant soybean cultivar treatment. There was no measurable effect on SCN population density when camelina or pennycress was included as a winter oilseed crop. The results indicate that camelina or pennycress can potentially be grown as winter oilseed cover crops in the soybean-corn rotations without significant risk to soybean production concerning SCN in Minnesota.

## Transcriptomic evaluation and R-gene profiling of resistance breakdown by *Meloidogyne chitwoodi* pathotype roza in wild potato *Solanum bulbocastanum*

Hu, Shengwei^1^, S. Bali^2^, R. Quick^3^, L. Cimrhakl^3^ and V. Sathuvalli^1^

^1^Oregon State University, Hermiston Agricultural R&E Center, Hermiston, OR 97838

^2^Simplot Plant Sciences, Boise, ID 83707

^3^USDA-ARS, Prosser, WA 99350

### Abstract

*Meloidogyne chitwoodi* (aka Columbia root-knot nematode or CRKN) is one of the most devastating pests of potato in the Pacific Northwest (PNW) that severely affects the overall plant health and tuber quality. Resistance to CRKN was discovered in the wild diploid potato species *Solanum bulbocastanum*, specifically in the accession known as SB22. This resistance trait was successfully incorporated into tetraploid potato clones, offering a promising avenue for managing CRKN infestations. However, in the Columbia Basin area, Roza pathotype of the CRKN can overcome the resistance derived from SB22. In this study, we inoculated SB22 with CRKN Race 1, to which SB22 is resistant, and the Roza pathotype, which can overcome this resistance. By comparing the transcriptomes of roots with different treatments, we observed distinct gene expression patterns for compatible and incompatible host-pathogen interactions. Our findings revealed that the Roza pathotype can suppress SB22’s immune response during the later stages of nematode infection. Furthermore, we identified the crucial role of plant hormones, such as salicylic acid-mediated signaling pathways, in the immune response of SB22 to CRKN. Through molecular mapping, we found several candidate resistance (R) genes that may contribute to CRKN resistance in SB22, and we analyzed their expression patterns. This research advances our understanding of the molecular mechanisms underlying potato host resistance to CRKN and provides valuable insights for enhancing resistance breeding in potatoes.

## Introgression of new source of resistance to Columbia root knot nematode in potato

Hu, Shengwei^1^, K. Swisher Grimm^2^, R. Quick^2^, L. Cimrhakl^2^ and V. Sathuvalli^1^

^1^Oregon State University, Hermiston Agricultural R&E Center, Hermiston, OR 97838

^2^USDA-ARS, Prosser, WA 99350

### Abstract

When present in potato fields, the Columbia root-knot nematode (CRKN, *Meloidogyne chitwoodi*) can cause serious damage to tubers, decreasing their value in both the fresh market and processing industries. Resistance to CRKN was first identified from the wild diploid potato species, *Solanum bulbocastanum* accession SB22, and was successfully introgressed into tetraploid potato breeding material. Unfortunately, a pathotype of CRKN known as *Roza*, was found in the Columbia Basin of Washington State which can break the resistance from SB22. In order to expand the genetic basis of CRKN resistance, previous screenings from our group identified clones from *Solanum hougasii* (6x) (PI283107hou-5mc, PI239423hou-8mc, and PI1239424hou-2mc) with significantly high levels of resistance against this pathotype. The resistant clones underwent pollination with 4x russet bulk pollen (Labelle Russet, Premier Russet, Dakota Trial Blazer, and Rainier Russet varieties). Subsequent hybrid seeds were germinated via tissue culture and subjected to ploidy analyses using plant cell flow cytometry to ascertain chromosome sets. Among the 13 pentaploid F1 progenies examined, two exhibited resistances to CRKN Roza pathotype. In BC1 progenies, four clones were identified as highly outcrossing and resistant to the Roza pathotype, but these also maintained pentaploidy. We subsequently generated a significant number of BC2 progeny to analyze their ploidy levels and resistance characteristics. From this group, we identified three clones that exhibited low ploidy levels and, despite being aneuploid, were nearly tetraploid. These clones also demonstrated resistance to the Roza pathotype and represent promising new sources of resistance to CRKN for potato breeding programs.

## Diversity and host attraction of *Pellioditis* spp. In Japan and pathogenicity of their symbiotic bacteria in slugs

Ichiishi, Kanata^1^, A. Sato^1^, N. Kanzaki^2^ and R. Shinya^1^

^1^Meiji University, School of Agriculture, Kanagawa, Japan

^2^Forestry and Forest Products Research Institute, Kansai Research Center, Kyoto, Japan

### Abstract

Symbiotic bacteria play crucial roles in host immunity, physiology, and metabolism, and they can also facilitate host adaptation by acting as biological weapons. *Pellioditis* spp., terrestrial gastropod parasites, harbor bacteria on or within their bodies that exhibit lethal effects on slugs. Previous studies have reported diverse bacterial associations in wild-isolated nematode species from European countries and the United States. However, the diversity of *Pellioditis* spp. and their associated bacteria in Asia remains largely unexplored. In this study, we examined the diversity of *Pellioditis* spp. and their bacterial symbionts from slugs and snails collected in Japan. We successfully isolated eight strains of five undescribed *Pellioditis* spp. and identified 11 bacterial strains of nine species. The pathogenicity of these bacterial isolates was assessed against two slug species, revealing species-specific variation in virulence. These findings suggest that *Pellioditis* spp. do not necessarily harbor bacteria with slug-killing capabilities, highlighting the complexity of their symbiotic relationships. Furthermore, we are currently conducting chemotaxis assays using slug and snail mucus, as well as volatile compounds, to elucidate the parasitic behavior of this nematode. This study provides new insights into the diversity and host interactions of *Pellioditis* spp. in Japan, contributing to a deeper understanding of their potential as biocontrol agents for pest gastropods.

## Rooting for corn: reproduction of root-knot nematode across multiple crops in Delaware

Irwin, Lauren and A. Betts

University of Delaware, Dept. of Plant and Soil Sciences, Georgetown, DE 19947

### Abstract

Southern root-knot nematode (RKN, *Meloidogyne incognita*) is a detrimental pest in Delaware agronomic and vegetable crop production systems. Reproduction of RKN is favored by soils with high sand content and crop rotation patterns that are all host crops. The primary agronomic rotation in the region is corn (*Zea mays*) and soybean (*Glycine max*). Corn is used as a rotation partner for other plant parasitic nematodes, like soybean cyst nematode (SCN, *Heterodera glycines*) and nematode sampling efforts are typically concentrated during the soybean rotation year. In 2023, grid sampling was established at two field sites planted with corn as part of a multi-year project connecting soil apparent electrical conductivity and nematode populations. The fields were divided into 1 acre (field 1) or 5 acre (field 2) grids and soil samples were collected at the beginning and end of season. Samples were processed to quantify the population of second stage juvenile (J2) RKN in 500 cm^3^ of soil. During these collections, RKN population increases as high as 35,000% were observed from individual grid quadrats from the beginning to the end of the season. Collectively, the selected sites saw an average RKN increase of 8,300% and 600% for fields 1 and 2, respectively. To further analyze RKN reproduction across host crops, a mini plot trial was established in 2024 to compare susceptible and resistant lima bean varieties, susceptible soybean, and corn. Crops were grown in three-meter-long row plots that were inoculated with 125 g of RKN-infected tomato roots at planting with four replicates for each host crop for a total of 16 plots. At crop maturity, soil and plant samples were collected for RKN quantification and root gall ratings using a 0–10 scale, with 0 having no galls present and 10 a completely galled, dead plant. Following ratings, eggs were extracted from 100 g of roots for each plot. The susceptible lima beans had the highest average soil sample populations (*P* = 0.01), gall ratings (*P* < 0.0001) and egg counts (*P* = 0.0005). Resistant lima bean, soybean, and corn had similar responses across evaluated metrics. While corn had no visible galls to rate, egg counts indicated reproduction. Due to the lack of notable above ground symptoms, the rate of RKN reproduction in Mid-Atlantic corn varieties has been underestimated and should be considered when designing crop rotation patterns.

## Cover crop practices to enhance nematode management, nitrogen utilization and water quality in Florida

Jacobs, Dustin^1^, J. Desaeger^1^, M. Lusk^2^, and Z. Grabau^3^

^1^University of Florida University, Dept. of Entomology and Nematology, Gulf Coast Research and Education Center, Wimauma, FL 33598

^2^University of Florida University, Dept. of Soil, Water and Ecosystem Sciences, Gulf Coast Research and Education Center, Wimauma, FL 33598

^3^University of Florida, Dept. of Entomology and Nematology, Gainesville, FL 32611

### Abstract

Cover crops in agricultural systems pose many potential benefits including erosion reduction, nutrient cycling, weed suppression, and nematode suppression. These benefits have the potential to reduce chemical inputs and synthetic fertilizer use in the cash crop season, as well as improve soil and water quality by reducing nitrogen leaching. Further research is needed to determine the effect of cover crops on these factors, especially in sandy soils found in Florida. The objective of this study is to observe the ability of cover crops to improve nitrogen utilization, nematode suppression, and support water quality in Florida. two experiments were conducted one in 2023 and another in 2024 to compare the effect of 6 cover crop treatments on these factors. Cover treatments crops were are sunn hemp (Crotalaria juncea), sorghum sudangrass (Sorghum bicolor x S.bicolor var. sudanense), cowpea (Vigna unguiculata), a 1:1 sunn hemp and cowpea mix, a 1:1 sunn hemp and sorghum sudangrass mix and a weedy fallow serving as the control. Cover crops were grown for approximately 80 days and then incorporated into the soil. Shortly after cover crop incorporation, vegetable beds were formed, and 4-week-old tomatoes (Solanum lycopersicum) were transplanted into them, where they grew for approximately 14 weeks. The predominant plant-parasitic nematode species during the cover crop growing season for both years were ring (Mesocriconema spp.) and lesion (Pratylenchus spp.) nematodes. In year one, lesion nematode populations in the soil were significantly higher in sunn hemp treatments compared to the control at 3 weeks after cover crop termination. Also, weed fallow supported significantly higher ring nematode populations at 3 weeks after cover crop termination; however, ring nematodes are not considered significantly damaging to tomato crops. The second year of this experiment showed similar results, with lesion and ring nematodes remaining predominant, and sunn hemp treatments again supporting higher lesion nematode populations compared to the control. No damage from either nematode species was observed on tomato plants in either year. First-year data showed higher total concentrations of extractable nitrate and ammonia in treatments containing sunn hemp at 3 weeks after cover crop termination (just before bed formation). Root-knot nematode infection was low in the first year, and no significant differences were observed in gall index or nematode population density. However, in the second year, root-knot nematode infection was more severe, and some differences were observed. Sorghum-sudangrass significantly reduced nematode populations in the soil and resulted in a significantly lower gall index compared to the control in end-of-season measurements. Yield was numerically increased by all cover crop treatments in both years, with notably the highest yield produced by plots treated with sorghum-sudangrass in year two. Soil nitrogen data is still being analyzed for the second year of this experiment.

## Shelterbelt planting and sheet mulching for nematode, soil health and weed management in a taro agroecosystem

Johnson, Angelina, Wang K.-H and Paudel R

University of Hawaii at Manoa, Dept. of Plant Environmental Protection Sciences, Honolulu, HI 96822

### Abstract

Resilient agriculture would be more eco-friendly by sourcing on-farm resources instead of relying on external inputs. Shelter belts are a possible source of on-farm inputs, and sheet mulching uses natural products to suppress weeds, conserve soil moisture, add organic matter, and improve soil health. This project studies the effect of shelterbelts and sheet mulching in a 2×2 factorial study (shelterbelt × sheet mulch) on a dryland taro farming system in Waianae, Hawaii. Two fast-growing leguminous trees, pigeon pea (*Cajanus cajan*) and gliricidia (*Gliricidia sepium*), were studied as shelterbelts. Shelterbelt plots (SB+) were planted with gliricidia along the border of the plot and pigeon pea every three rows within the plot and compared to plots without shelterbelts (SB−). Sheet mulch plots (SM) were covered with layers of mushroom compost waste, recycled cardboard, pigeon pea clips, and wood chips (bottom to top) and compared to plots with an air-impermeable synthetic mulch (NM). Taro (*Colocasia esculenta*) suckers were planted in December 2024. Four months after taro planting, showed no significant difference in volumetric water content (VWC), soil microbial respiration (CO_2_), or water infiltration among treatments. However, SM resulted in taller taro plants (*P* ≤ 0.05) and required significantly less weeding time than NM (*P* ≤ 0.05) as weeds were growing out from the planting hole on the synthetic mulch. Unexpectedly, SB+ had a higher weeding time compared to SB− (*P* ≤ 0.05), most probably because weeds were encroaching from the unmulched shelterbelt planted rows. Taro leaf number per plant was higher in SB− than in SB+ (*P* ≤ 0.05) likely due to SB+ having higher weed pressure which reduced taro growth. While this on-farm research is in its early stage of evaluation, it builds off of a preliminary field trial conducted at the Magoon Research Station (Honolulu, Hawaii) that compared soil under sheet mulch (SM) and woven polyester weed mat (WM) to bare ground (BG), each with 3 replicated strips of 1×5 m^2^ area. Two months after mulch installment, SM reduced weed abundance at par to WM, and better than BG (*P* ≤ 0.05). WM significantly reduced soil microbial respiration, while SM maintained soil respiration similar to BG (*P* ≤ 0.05). SM also increased the nematode enrichment index (indicating soil was dominated by bacterial decomposition and nutrient enrichment) and nematode richness. This preliminary trial suggested that SM improved soil health within a short period, and that weed mat could impose negative effects on soil health. The results from the Waianae field research also suggest that SM can enhance soil health and reduce weeding time. However, the benefits of SM and SB in improving other soil properties, such as infiltration and soil microbial respiration, might require a longer term to determine.

## A novel species of free-living marine nematode from the great Salt Lake described in morphological and molecular detail

Jung, Julie^1,2^, T. R. Murray^1^, M. C. Marcue^1^, T. Powers^3^, S. Farrer^4^, A. Borgmeier^4^, B. J. Adams^4,5^, G. Fonseca^6^ and M. S. Werner^1^

^1^University of Utah, School of Biological Sciences, Salt Lake City, UT, 84112, USA

^2^Weber State University, Department of Zoology, Ogden, UT, 84403, USA

^3^University of Nebraska-Lincoln, Department of Plant Pathology, Lincoln, NE, 68583-0722, USA

^4^Brigham Young University, Department of Biology, Provo, UT, 84602, USA

^5^Monte L. Bean Museum, Brigham Young University, Provo, UT, 84602, USA

^6^Federal University of São Paulo, Marine Science Institute, Santos, São Paulo, 04021-001, Brazil

### Abstract

Herein we present a novel species of nematode, *Diplolaimelloides woaabi* sp. nov. (family Monhysteridae, order Monhysterida), discovered in microbialites of the Great Salt Lake, Utah, USA. This species is notable for its adaptation to hyper saline microbialite habitats, positioning it as both an extremophile and a potential bioindicator of ecological change in the Great Salt Lake. We used scanning electron microscopy and differential interference contrast microscopy to characterize *D. woaabi* by the combination of the following: ocelli present; a relatively small body size (<1.5 mm); short anterior sensory setae; cryptospiral amphidial fovea located shortly posterior to buccal cavity base; a funnel-shaped anterior buccal cavity and reduced secondary cavity; fused lips; long double spicules and conspicuous male bursa displaying four pairs of post-cloacal papilla arranged in a (2+2) pattern, a single mid-ventral pre-cloacal papillae, two additional pairs of papilla posterior to the bursa, and an additional offset mid-tail papillae pair. Next, we assembled our draft genome of *D. woaabi* using Multiple Displacement Amplification and a combination of Nextera short read sequencing and Oxford Nanopore long reads. Finally, we assembled a draft transcriptome using Trinity. Although the amount of DNA in single nematodes has posed a historical challenge for sequencing approaches, newer methods are emerging that lower the effective input requirements for library preparation. Implementing these methods with wild-isolates of *Diplolaimelloides* has great potential to update its systematics, and serve as a model system for the evolution of animals to extreme environments.

## Development of recombinase polymerase amplification assay for the rapid detection of beech leaf nematode

Kantor, Mihail^1^ and S. A. Subbotin^2^

^1^Plant Pathology & Environmental Microbiology Department, The Pennsylvania State University, University Park, PA, 16802, USA

^2^Plant Pest Diagnostic Center, California Department of Food and Agriculture, 3294 Meadowview Road, Sacramento, CA, 95832, USA

### Abstract

Beech leaf nematode, *Litylenchus crenatae mccannii* (Lcm), is the causal agent of beech leaf disease (BLD), a condition characterized by interveinal dark-green banding, leaf thickening, bud abortion, and potentially tree mortality. Current identification methods for Lcm rely on morphometric analysis or molecular techniques such as conventional PCR and real-time PCR. To support timely management of BLD, there is a need for a rapid, field-deployable detection method. Recombinase polymerase amplification (RPA) is a new isothermal *in vitro* nucleic acid amplification technique that has been adopted for rapid and reliable diagnostics of nematodes. In this study, the real-time RPA assay and RPA assay combined with lateral flow dipsticks have been developed targeting the ITS rRNA gene of Lcm. The assays demonstrated high specificity, sensitivity, enabling direct detection from crude nematode extracts without requiring DNA extraction. Assay specificity was validated against a range of non-target nematode species. The RPA assays present a practical diagnostic tool for plant clinics and forestry professionals, enabling rapid, on-site detection of Lcm in infested beech trees to support timely disease management decisions.

## Promising indigenous bacterial isolates for biological control of guava root-knot nematode, *Meloidogyne enterolobii*

Kassam, Rami and A. Hajihassani

Department of Entomology and Nematology, Fort Lauderdale Research and Education Center, Institute of Food and Agricultural Sciences, University of Florida, Davie, FL 33314

### Abstract

Guava (*Psidium guajava*) is threatened by an aggressive species of root-knot nematode (*Meloidogyne enterolobii*) in South Florida, necessitating efficient management strategies. This study explored the local bacterial diversity as a potential alternative biological control against *M. enterolobii*, providing an environmentally friendly solution. Twenty-five *M. enterolobii-*infected root and rhizospheric soil samples were collected from four guava fields in Homestead, FL, and used for isolation of antagonistic bacteria using nematode egg masses and juveniles (J2s) as baits. Molecular diagnostics were employed to identify the isolated bacteria by sequencing the 16S rRNA gene. Three bioassays were conducted to evaluate the nematicidal activity of bacterial secondary metabolites (SMs), filtrates and cultures after a 24-hour exposure period. The most effective isolates were then assessed for their ability to inhibit *M. enterolobii* penetration into the roots of susceptible tomato, used as a model crop. We identified 39 bacterial strains belonging to different genera and species. The secondary metabolites of *Pantoea agglomerans*, *Delftia tsuruhatensis*, *Stenotrophomonas* sp., *Serratia* sp. and *Pseudomonas putida* caused more than 90% mortality rates against J2s, significantly higher than the untreated control (2.4%). Filtrates of *Enterobacter pseudoroggenkampii*, *D. tsuruhatensis*, *P. agglomerans*, *Achromobacter xylosoxidans*, *Bacillus* sp. and *Staphylococcus gallinarum* induced mortality rates above 80% against J2s compared to the control (3.02%). Additionally, *Stenotrophomonas* sp., *Bacillus* sp., *A. xylosoxidans* and *Stenotrophomonas maltophilia* cultures in water agar plates revealed approximately 40% mortality rates against J2s compared to the control (1.2%). We also found that *D. tsuruhatensis, Stenotrophomonas* sp., *Serratia* sp., *P. agglomerans* and *Bacillus* sp. significantly reduced nematode J2 penetration into roots by 96–98%. These results highlight the potential of the most efficacious bacterial isolates as biological control agents against *M. enterolobii*. Further in-depth studies are necessary to elucidate the impacts of these bacteria on nematode development and reproduction in guava and other host crops under both controlled and field settings.

## Characteristics of the free-living nematode community associated with the suppression of Root-Knot Nematodes in four farmlands in Japan

Kato Risako^1^, V. S. Nguyen^2^, and K. Toyota^1^

^1^Graduate School of Bio–Applications and Systems Engineering, Tokyo University of Agriculture and Technology, Tokyo, Japan

^2^College of Soil Science, Can Tho University, Can Tho Vietnam

### Abstract

Biological control is expected as an alternative method of chemical control of root-knot nematodes (RKN), but it is unstable because of influence by the surrounding environment. Since free-living nematode (FLN) communities are used as indicators of soil condition, we hypothesized that the community might be an indicator for the soil environment in which biological control works well. We conducted a pot experiment, aiming to find characteristics of FLN communities associated with high RKN suppressive potential. Soils were collected from four farmlands around Tokyo in Japan. The sampling sites were selected from an organic farming (S farm), a conventional farming without fumigation (Y farm), and two conventional farming fields with fumigation (I farm and M farm) to compare the effects of farming management on RKN suppression. FLN communities were identified morphologically, then a pot experiment was conducted using nonautoclaved and autoclaved soils to evaluate biological suppression of each field soil. Green pepper plants were grown in the soil as a model plant, and RKN J2s were inoculated. After 6 weeks, we counted the number of egg masses and performed a correlation analysis between RKN suppression and FLN community characteristics. Most of the indices (Structure index, Shannon index, and Richness) were the highest in the organic field. In all soils, the number of egg masses tended to be lower in non-autoclaved soils, and significant differences were seen in Y farm and M farm. Y farm had the lowest ratio of the number of egg masses in non-autoclaved to autoclaved soil, indicating that the field had the highest suppression. The abundance of fungivores and bacterivores had a significant positive correlation with the suppression (*R*^2^ = 0.96 and 0.95, respectively, *P* < 0.05), but the abundance of predators or omnivores did not. Furthermore, the relative abundance of omnivores was positively correlated with the number of egg masses (*R*^2^ = 0.89, *P* = 0.057). The results suggested that microbial-feeding nematodes played a more important role in suppressing RKN than predators or omnivores. Many papers have reported the relationship between the population of predators or omnivores, but the contribution of microbial feeders has rarely been considered. However, this study suggested that microbial feeders might suppress nematodes.

## Characterization of resistance mechanisms in soybean accession PI438489b against *Meloidogyne incognita*

Kaur, Anmolpreet^1^, S. Chhapekar^2^, H. Nguyen^2^, T. Faske^1^, C. Vieira^1^ and J. Kud^1^

^1^Department of Entomology and Plant Pathology, University of Arkansas, Fayetteville, AR, 72701, USA

^2^Division of Plant Sciences and Center for Soybean Biotechnology, University of Missouri-Columbia, Columbia, MO, 65211, USA

### Abstract

Southern root-knot nematode (SRKN, *Meloidogyne incognita*) is a destructive plant-parasitic nematode that causes substantial yield losses in soybean production, particularly in the southern United States. In Arkansas alone, SRKN inflicted an estimated $100 million in annual losses for soybean growers in 2023, reflecting a concerning upward trend over the past decade. Previous studies identified a quantitative trait locus, *Resistance to Meloidogyne incognita 1* (*Rmi1*), on chromosome 10 as a source of resistance to SRKN in few soybean lines. However, unlike resistance in other crops such as tomato, pepper, or cotton—where classical resistance (R) genes are typically involved—the *Rmi1* region lacks a conventional R gene, and the underlying defense mechanism remains unclear. To address this knowledge gap, we evaluated SRKN penetration and infection progression in four soybean genotypes: the susceptible cultivar Magellan, the resistant line PI 438489B, and two nearly isogenic lines (NILs) derived from a Magellan × PI 438489B cross. Each plant was inoculated with 1,000 second-stage juveniles (J2s) from a lab-maintained SRKN population. Nematode quantification and developmental stages were evaluated at 7, 14, 21, and 28 days post-inoculation (dpi) using Acid Fuchsin staining method. Our results showed significantly fewer nematodes in the roots of PI 438489B compared to Magellan, along with delayed nematode development and a markedly reduced number of nematodes reaching the female stage by 28 dpi. Gall scoring at 28 dpi and egg count at 35 dpi confirmed that PI 438489B not only reduces gall formation but also suppresses SRKN reproduction. The NILs exhibited intermediate phenotypes. In addition, we conducted comparative RNA-seq analysis between susceptible Magellan and resistant PI 438489B at 0, 2, 7, and 14 dpi to identify molecular pathways involved in the defense response to shed light on potential mechanisms of resistance. To further assess the durability of resistance, we evaluated galling and nematode reproduction of four SRKN isolates, collected from diverse geographic regions and cropping histories across Arkansas, on both Magellan and PI 438489B plants. The results revealed variation in isolate virulence, suggesting that *Rmi1*-mediated resistance could be vulnerable to resistance-breaking SRKN populations in the future. These findings provide important insights into the mechanisms of SRKN resistance in soybean and highlight the complexity of plant-nematode interactions. Characterizing novel sources of resistance is crucial for developing durable resistance and advancing sustainable nematode management strategies in soybean production.

## Turning invasive weeds and industrial waste from Japan into vermicompost tea for sweetpotato nematode management

Kawasumi, Ruka^1^, R. Paudel^2^, K.-H. Wang^2^ and M. Tojo^1^

^1^Osaka Metropolitan University, Sakai, Osaka 5998531

^2^University of Hawaii at Manoa, Honolulu, HI 96822

### Abstract

As part of the efforts to reduce waste in Japan, this project investigates in vermicomposting using invasive bamboo (*Phyllostachys edulis* (Carrière) J. Houz.) and rapeseed (*Brassica napus* L.) oil cake, a byproduct of edible oil production, as feedstock. The vermicompost tea generated has been shown to suppress damping-off disease in perilla (*Perilla ocymoides* L.) and cucumber (*Cucumis sativus* L.). The objective of this study was to evaluate the suppressive effects of vermicompost tea (VCT) on root-knot (*Meloidogyne incognita*) and reniform (*Rotylenchulus reniformis*) nematodes. Two sweetpotato (*Ipomoea batatas* (L.) Lam) pot trials were conducted, one in 1.4-L greenhouse pots, and one in 38-L field pots. Both trials used soil naturally infested with *M. incognita* and *R. reniformis*, planted with ‘Okinawan’ sweetpotato. Plants were drenched with 1) VCT derived from vermicompost using invasive bamboo and rapeseed oil cake as feedstock (VTB) fed only once during the production process, 2) VCT derived from vermicompost using vegetable scraps as feedstock (VTV) fed weekly, or 3) water as a control. Greenhouse pots were drenched weekly for 12 weeks, whereas field pots were drenched every 2 weeks for 18 weeks. At the termination of the greenhouse trial, VTB numerically reduced infection rates of plant-parasitic nematodes based on acid Fuchsin root staining, enriched soil nutrients as indicated by higher Enrichment Index (EI, n=5, *P*≤0.05), and enhanced soil food web structure as reflected in higher Structure Index (SI) compared to control (*P*≤0.05). Nematode metabolic footprint in VTB treated pots resulted in a relatively square rhombus, approaching a structured and enriched soil food web, whereas that in the control was a narrow rhombus resting in a nutrient depleted and disturbed soil food web trajectory. These findings were supported by numerically higher soil microbial respiration and ammonia-nitrogen concentrations in VTB than the control. Performance of VTB in the field-pot experiment was less encouraging, with no clear suppression on the plant-parasitic nematodes, though VTB increased EI numerically. On the other hand, VTV increased SI (n=6, *P*≤0.05), generated 1.5-fold higher ammonia-N and resulted in 2-fold increase in sweetpotato tuberous root weight compared to the control. Nematode metabolic footprint revealed generally a poorer soil health condition in the field-pot experiment than the greenhouse-pot experiment. Under poorer soil health, VTV prepared from weekly fed feedstock was able to maintain a relatively square rhombus metabolic footprint and allow the soil food web to reach a structured and enriched condition. While better performance of VTB in the greenhouse compared to the field experiment could be attributed to weekly rather than biweekly drenching, periodically restocking the vermicompost feedstock with fresher materials might be the main reason VTV outperformed VTB on the hind side. It is encouraging to see drenching vermicompost tea using waste as feedstock could improve soil health and suppress plant-parasitic nematodes in a short period of time.

## Sustainable pest management for small-scale organic sweetpotato production in Hawai‘i

Kekoa Larger, R. Paudel and K.-H. Wang

Department of Plant and Environmental Protection Sciences, University of Hawaii at Manoa, Honolulu, HI 96822

### Abstract

Sweetpotato (*Ipomoea batatas*) is a key vegetable crop in Hawai‘i, with a farm gate value of $2.2 million in 2022 and 2.5 million pounds produced, making it the most produced vegetable by weight in Hawai‘i. Unfortunately, this level of production was far less than the $7.3 million farm gate value (16.7 million pounds) reported in 2011. The main culprits are the weevils *Cylas formicarius* (sweetpotato weevil), *Euscepes postfasciatus* (West Indian sweetpotato weevil), and *Blosyrus asellus* (rough sweetpotato weevil) and plant-parasitic nematodes (root-knot nematode, *Meloidogyne* spp., and reniform nematode, *Rotylenchulus reniformis*). The management challenges for these pests are significant for organic farmers. Thus, the objectives of this study were to evaluate the efficacy of several biopesticides and locally isolated biological control agents against weevils and nematodes in an organic cropping system in Hawai‘i. A field trial was conducted to examine 6 treatments on two Okinawan sweetpotato varieties from December 2023 to May 2024 at Poamoho Experiment Station, University of Hawaii. These treatments included: Mycotrol^®^ ESO (a.i. *Beauvaria bassiana*, Certis L.L.C., Columbia, MD) as basal foliar spray, MeloCon^®^ LC (a.i. *Purpureocillium lilacinum*, Certis L.L.C.) as soil drench, Molt-X^®^ (a.i. Azadirachtin, Bioworks, Inc., Victor, NY) as soil drench, a locally isolated *Metarhizium anisopliae* ‘Ko-002’ incorporated into compost as a soil amendment at planting, a combination of all the above treatments (Combo), and an untreated control. The two Okinawan varieties were sourced from 1) virus-free tissue-cultured propagated plants (TC), and 2) non-tissue cultured source (TB). Each treatment was replicated three times in 4.5-m^2^ plots. Field plots were treated monthly beginning at 1 month after planting. The Combo entailed monthly basal sprays of Mycotrol^®^ and alternate drenching of MeloCon^®^ or Molt-X^®^ in rotation. Soil samples were collected at planting, mid-term, and at harvest (6 months after planting). Sweetpotato tuberous roots were harvested, graded and weighed. Unfortunately, due to the high pressure of weevils, all biopesticides failed to control weevil damage with overall marketable yield < 10%. However, the total weight of sweetpotato was 3.4 times higher from TC than TB, indicating planting more vigorous sweetpotato cuttings reduced weevil and nematode yield loss. At harvest, MeloCon^®^ and *Metarhizium* compost suppressed abundance of *R. reniformis* (*P* ≤ 0.05); whereas Molt-X^®^ and Combo reduced abundance of *Meloidogyne* spp though the results were not significant. Overall, the Combo treatment was most promising because it almost eliminated *Meloidogyne* in the soil at harvest, and reduced the number of *R. reniformis* by half of that in the untreated control. Though weevil damage was not reduced in this experiment, the Combo treatment produced the highest total yield compared to zero harvested from the untreated control. This research revealed the viability of *rhizium anisopliae* for plant-parasitic nematode management, and the importance of rotating or combining different bionematicides to manage multiple genera of plant-parasitic nematodes that co-exist in the same field.

## Fine root traits and soil properties are major factors driving soil bacteria, fungi, and nematodes in the rhizosphere of four coniferous tree species

Kitagami, Yudai^1^ and N. Makita^2^

^1^Mie University, Graduate School of Bioresources, Tsu, 514-8507

^2^Shinshu University, Faculty of Science, Matsumoto, 390-8621

### Abstract

Soil organisms include mycorrhizal associations, microorganisms such as bacteria and fungi, and fauna such as nematodes across a wide range of sizes and trophic groups, contributing complex food webs. Among them, soil nematodes are a major group of soil microfauna and the most abundant animals on Earth. They play crucial roles in ecosystem processes, such as improving soil physicochemical properties and participating in carbon and nitrogen cycling by feeding on bacteria and fungi. However, our understanding of root-mediated effects of trees on the composition and diversity of soil biota remains limited. This study aimed to determine how root traits of four coniferous tree species and the soil properties they influence soil biological communities in plantations. We took soils and fine roots at monocultural plantations of *Pinus densiflora* (pine), *Larix kaempferi* (larch), *Chamaecyparis obtusa* (cypress), and *Cryptomeria japonica* (cedar) in Terasawayama Research Station Forest, central Japan. Bacterial and fungal compositions of soils were estimated with metabarcoding using the MiSeq high-throughput sequencing system. In contrast, soil nematodes were morphologically identified at the genus/family level and examined for both community structure and trophic composition. To clarify the effects of root morphological traits on each biological community, the total root length, surface area, volume, and mean diameter of each sample were analyzed using WinRHIZO Pro 2013. Our results showed that the number of fungal ASVs based on amplicon sequencing variances (ASVs) was significantly higher at the cypress (150±10 ASVs) than the pine (68±3 ASVs). The alpha diversity of nematode taxa was significantly higher at the cedar (25±1 taxa) than the pine (14±1 taxa). The number of fungal ASVs and nematode taxon richness were significantly correlated with root traits, such as specific root length or root C/N ratio, and soil properties, such as soil pH or soil C/N ratio. Bacterial, fungal, and nematode community compositions were significantly different between the four tree species. Moreover, soil properties (e.g., soil pH, C/N, and water contents) significantly influenced the community structure of all three soil biota. In the functional guilds of fungal taxa, ectomycorrhizal fungi predominated in the pine and larch plantations, whereas saprotrophic fungi did in the cypress and cedar. In the trophic group of nematodes, the relative abundance of fungivorous nematodes was greater at pine, larch, and cypress plantations, while herbivorous ones were greater in cedar plantations. Our study showed that the fungal and nematode alpha diversity were well explained root traits and soil properties. Moreover, the composition of the soil biological community depends on its physicochemical properties, which are influenced by the root traits associated with different forest types. These findings suggest that tree species shape unique belowground biological communities through their influence on both root systems and the surrounding soil environment.

## Probiotic capsule that boosts potato immunity against Root-Knot Nematodes

Ko, Itsuhiro^1,2^ and C. Gleason^2^

^1^Program of Molecular Plant Sciences, Washington State University, Pullman, WA, 99164

^2^Department of Plant Pathology, Washington State University, Pullman, WA, 99164

### Abstract

The goal of this project is to encapsulate the *Bacillus subtilis* secreting a defense elicitor, StPep1, into capsules that can be amended into soil, providing a means of potato protection against root-knot nematodes. Root-knot nematodes (RKN, *Meloidogyne* spp.) are soil-borne pathogens that infect plant roots worldwide. In the Pacific Northwest region, the RKN *M. chitwoodi* is a major problem in the potato industry, where tuber damage by this RKN can significantly affect the potato market value. Due to environmental, health, and economic concerns, the longstanding RKN control method of using chemical nematicides is not favored by producers and consumers. In addition, there is no genetic resistance to RKNs in commercially available potato cultivars; therefore, novel control strategies are needed. A recent study demonstrated that, under controlled environmental conditions, watering potatoes with a solution of *B. subtilis* secreting the potato peptide elicitor 1 (StPep1) decreased RKN root galling. However, there are challenges in the transportation and handling of liquid bacterial cultures for field application. To transform this idea into an applicable solution for growers, we enhanced the ability of *B. subtilis* to produce StPep1 and then encapsulated the bacteria into alginate capsules, which are a better solution for transport, storage, and application of the bacteria. We hypothesized that following an application into the soil, the capsules would slowly degrade over a prolonged time, releasing StPep1-secreting *B. subtilis* near the potato roots and providing plant protection from RKN. First, the *StPep1* gene was incorporated into a vector that improved protein production in *B. subtilis*, resulting in 2~3 times more StPep1 production/secretion in liquid culture compared to the control. Application of this *B. subtilis* to potato resulted in a 33% reduction in the galls formed by *M. chitwoodi*. Then this *B. subtilis* was encapsulated into calcium-alginate capsules. To assess the viability of this bacteria encapsulation approach for field applications, the capsules were monitored for StPep1 levels, *B. subtilis* growth, and capsule longevity in the soil over a time course. The *B. subtilis* capsules began to degrade in soil after 7 days, slowly releasing the protectant bacteria in the treated area. Our results showed that using the formulated alginate capsules was a successful approach to maintain viable *B. subtilis,* which produced StPep1 for 60 days following soil application and can be stored up to a year on the bench. The efficacy of *B. subtilis* capsules to control RKN infection on potato roots is currently under investigation. Our long-term goal is to provide growers a sustainable alternative for pathogen biocontrol in potatoes and beyond through this capsule treatment.

## Engineering plant resistance, what tools do we have to advance nematode management?

Kud, Joanna^1^, L. Huang^2^, Y. Yuan^2^ and F. Xiao^2^

^1^Department of Entomology and Plant Pathology, University of Arkansas, Fayetteville, AR, 72701, USA

^2^Department of Plant Sciences, University of Idaho, Moscow, ID, 83844, USA

### Abstract

Host genetic resistance is one of the most desirable strategies for managing plant-parasitic nematodes (PPNs), providing an environmentally safe and economically viable solution for growers while supporting sustainable agriculture. However, breeding for resistance faces significant challenges due to the limited pool of resistance sources. As a result, even when multiple resistant varieties are deployed, growers may unintentionally expose nematode populations to the same core resistance genes—creating ideal conditions for resistance erosion. These limitations highlight the urgent need to explore biotechnological alternatives that can accelerate the development of novel resistance strategies. One promising avenue is genome editing, with tools like CRISPR offering precise and minimal genetic modifications to enhance plant disease resistance. However, engineering durable host resistance requires a deeper understanding of the plant immunity to strategically target key defense components. The introduction of canonical resistance (R) genes through breeding has been a favored approach for generating pest resistance, as a single gene can often provide strong protection. Yet molecular studies of plant–nematode interactions reveal that relying on a single R gene frequently results in resistance breakdown. A striking example is the potato cyst nematode *Globodera pallida* effector RHA1B, which functions as an E3 ubiquitin ligase. RHA1B promotes nematode virulence by ubiquitinating and degrading host proteins. Our research has shown that RHA1B specifically targets the membrane-bound pattern recognition receptor NILR1, which detects the nematode pheromone Ascr#18, to trigger immune responses. In addition, RHA1B triggers degradation of intracellular R proteins such as Gpa2, which play a role in effector-triggered immunity. This example demonstrates that nematode success often stems not from the absence of host defenses, but from the ability to bypass or disable them. Importantly, this insight opens opportunities to engineer receptor variants that evade effector recognition, thereby restoring their defensive function, potentially by mimicking the natural allelic diversity found in wild crop relatives to which nematodes are not yet adapted. Alternatively, biotechnological engineering can also target susceptibility factors defined as host genes exploited by nematodes to complete their life cycle. Most economically significant PPNs are sedentary endoparasites that reprogram host developmental and metabolic pathways to form long-term feeding sites. Interestingly, RHA1B also targets RNA metabolism machinery, crucial for host cell cycle regulation involved in feeding site establishment. Disrupting such susceptibility factors is a promising alternative to achieving durable nematode resistance. In addition to targeting host proteins, RHA1B also regulates the stability of other nematode effectors, adding a new layer of complexity to the nematode effectorome. Thus, host-induced gene silencing (HIGS) targeting this powerful nematode metaeffector represent yet another biotechnological approach that can be levraged to engineer nematode resistance. Overall, our case study on RHA1B illustrates how insights into effector virulence mechanisms can guide future targeted genome editing and RNAi strategies to shield crops from PPNs.

## The biological control potential of *Pristionchus pacificus*, *Oscheius myriophilus* and their symbiotic bacteria against fall armyworm

Kuo, Hao-Yu^1,2^, S. Huang^2^, E. Pini^2^, S. Chang^2^, F. Hu^3^, W. Chuang^3^ and J. Yang^1,2^

^1^Department of Nematology, University of California, Riverside, 900 University Ave, Riverside, CA, 92521

^2^Department of Plant Pathology and Microbiology, National Taiwan University, No. 1 Roosevelt Rd., Sec. 4, Da-an Dist., Taipei City, 10617, Taiwan

^3^Department of Agronomy, National Taiwan University, No. 1 Roosevelt Rd., Sec. 4, Da-an Dist., Taipei 10617, Taiwan

### Abstract

The Fall Armyworm (*Spodoptera frugiperda*; FAW) is recognized by the United Nations Food and Agriculture Organization (FAO) as a major global agricultural pest. It invaded Taiwan in 2019, causing significant damage to agricultural crops such as corn, sorghum, and rice. Entomopathogenic nematodes (EPNs) and their symbiotic bacteria are effective and environmentally friendly biocontrol agents commonly used in integrated pest management (IPM) strategies. However, due to restrictions from environmental conditions, climate, and product storage, there are currently no commercially available products derived from local entomopathogenic nematode populations in Taiwan. This study aimed to identify native EPNs and their symbiotic bacteria and assess their potential for biological control of FAW. Through pupae-baiting and white traps, 4 EPNs, molecularly identified as *Pristionchus pacificus* (PP-6) and *Oscheius myriophilus* (OM-16, OM-G1A1 and OM-G1B1), were obtained from local soils. Three concentrations of the EPN (2000, 3000, 4000 IJs/ml) were first examined for efficacy through injection. The FAW 3^rd^ instar larvae mortality rate reached 23% with the PP-6 isolate at 4000IJs/ml, while no significant differences were observed among the concentrations tested. In the greenhouse experiment, OM-16 and OM-G1B1 led to 15% and 21% mortality rate of the 3^rd^ instar larvae at 2000IJs/ml, respectively. PP-6, OM-G1A1 and OM-G1B1 affected the mass of live FAW larvae 72 post-inoculation. The isolation of symbiotic bacteria was conducted using NA, Tergitol-7, and MacConkey agar media. *Serratia marcescens* and *Achromobacter insuavis* were found in *P. pacificus*, while *Cupriavidus* spp., *Pseudomonas* spp., *Variovorax* spp., and *Stenotrophomonas* spp. were associated with *O. myriophilus*. In pathogenicity experiment, strains *C. malaysiensis* G1A1-3 and *V. paradoxus* G1B1-1 caused high larval mortality in FAW (41.11% and 30%, respectively). Furthermore, the bacterial suspension of 6 bacteria strains inhibited FAW pupae eclosion. Overall, our study suggested the newly isolated EPNs and their symbiotic bacteria have a lethal effect on FAW larvae or pupae. These results provide a foundation for the future development of the EPN application in controlling FAW.

## Automated imaging and taxonomy-guided AI for accurate and scalable soil biodiversity diagnosis

Kurfürst, Vojtěch^1^, Z. Matar^1^, M. Kolarink^1^, A. Cervenka^1^, K. Tanahashi^1^, S. Zarei^1^, G. Verheijen^1^, A. Wigman^1^ and R. Janissen^1,2^

^1^Veridi Technologies, The Hague, 2514GJ, The Netherlands

^2^Institute of Bioengineering, Deggendorf Institute of Technology, Oberschneiding, 94636, Germany

### Abstract

Soil biodiversity is crucial for our functional biosphere and 95% of our food relies on healthy soil. Yet over 70% of earth’s soil is degraded, highlighting the urgency to restore soil health, a goal emphasized by the recent European soil monitoring directive. Soil-born nematodes exist at all trophic levels of the soil food web and are a universal bioindicator of soil biodiversity, even in degraded soils. However, this indicator is not widely used and requires nematologist and soil ecology experts as well as significant labor-intensive manual analyses. With the support of EIC and EIT, we developed an automated end-to-end diagnosis tool, comprised of an automated soil sample imaging system and a multi-level, taxonomy-guided AI for nematode species identification. Our technology provides quantitative soil biodiversity parameters, assessing soil health, immunity, fertility, parasites, carbon cycling, pollution, and organic degradation pathway. Validated by research and phytopathogenic laboratories, the tool demonstrated to be more accurate (>90%) and over 20-times faster (<15 min) in end-to-end biodiversity analysis than manual analysis. This technology allows scalable diagnostics with the potential to become a new standard in commercial and research sectors, aiding in the global efforts to restore and manage soil health.

## Seed-delivered shield: cyclobutrifluram’s field performance against key cotton nematodes

Lawaju, Bisho Ram and K. Lawrence

Auburn University, Department of Entomology and Plant Pathology, Auburn, AL 36849

### Abstract

Reniform (*Rotylenchulus reniformis*) and root-knot (*Meloidogyne incognita*) nematodes remain major constraints to cotton production in Alabama and across all the cotton producing states in the United States. Yield losses attributed to reniform, and root-knot nematodes are estimated at $26 and $24 per acre, respectively, translating to annual national losses of $10.7 million and $9.8 million. In severely infested fields, yield reductions of up to 50% have been observed, highlighting the urgent need for effective nematode management strategies. This study evaluated the efficacy of cyclobutrifluram, a new succinate dehydrogenase inhibitor (SDHI) nematicide developed under Tymirium™ technology, for managing these economically significant nematodes in cotton. Over three years, six field trials were conducted—three each in fields infested with root-knot and reniform nematodes. Treatments included: (1) cyclobutrifluram applied as a seed treatment (0.45 mg active ingredient [ai]/seed), (2) a commercial seed treatment standard (BIOST Nematicide 100 at 521 mL/100 kg seeds), (3) in-furrow granular aldicarb (AgLogic 15G at 5.0 lb/A), and (4) an untreated control. All trials followed a randomized complete block design with five replications per treatment. Each block consisted of two 25-foot-long rows, with 36- to 40-inch row spacing. Cotton was planted in early May each year. At approximately 40 days after planting (DAP), four plants per plot were randomly selected for assessment of growth parameters and nematode population densities. Nematode eggs were extracted using 0.75% NaOCl solution and recovered on a 25 µm sieve. At maturity, seed cotton yields were recorded following mechanical harvest. Statistical analysis was conducted using SAS 9.4 (PROC GLIMMIX), and treatment means were compared using the Tukey-Kramer test at a significance level of *P* ≤ 0.05. Results showed that plant stands across all treatments were within the optimal range of 1.6 to 2.9 plants per foot for cotton production. In root-knot nematode trials, no significant differences in plant height, shoot, or root biomass were observed among treatments. However, in reniform nematode-infested fields, seed treatment with cyclobutrifluram significantly improved shoot and root biomass, with average increases of 51% and 46% over untreated control. Cyclobutrifluram also resulted in significant nematode suppression, reducing reniform nematode egg counts per gram of root by 78% (*P* = 0.0012) and root-knot nematode eggs by 52% (*P* = 0.0023). Yield gains reflected these reductions. In reniform nematode trials, cyclobutrifluram increased seed cotton yield by 159 lb/A, with an estimated economic benefit of $45 per acre. Similar yield gains and economic returns were observed in root-knot nematode trials, too. On average, cyclobutrifluram provided an additional return of $45/A across all trials. These findings demonstrate that cyclobutrifluram is an effective seed-applied nematicide for managing both reniform and root-knot nematodes, offering a promising addition to integrated nematode management strategies in cotton production systems.

## *Rotylenchulus reniformis* movement and population growth in cotton

Lawrence, Kathy S^1^, S. R. Moore^2^ and B. R. Lawaju^1^

^1^Auburn University, Auburn, Alabama

^2^Syngenta Crop Protection, LLC, Monroe, Louisiana

### Abstract

The movement and population dynamics of *Rotylenchulus reniformis* were evaluated following initial introduction into a cotton (*Gossypium hirsutum*) field under irrigated and non-irrigated conditions on a Decatur silt loam soil (S-S-C = 23-49-28). Vermiform females and juveniles colonized the adjacent cotton row (100 cm from the point of inoculation) by 90 days after planting (DAP), and the second row (200 cm) by 150 DAP in both irrigation treatments. In the irrigated trial, males reached 150 cm by 60 DAP and 200 cm by 90 DAP; in the non-irrigated trial, they reached only 100 cm by 60 DAP and 200 cm by 90 DAP. The average rate of movement was 1.67 cm/day. Populations were highest in the inoculated row and generally greater within cotton rows than in row middles. By 150 DAP, *R. reniformis* was detected to a depth of 91 cm, with highest densities occurring in the top 15 cm. A complementary study using 7.62 cm diameter × 76 cm deep soil cores examined the effects of water infiltration and root growth on vertical nematode migration. Minimal downward movement occurred under low water infiltration. Rainfall simulations of 2.54 cm^3^, 7.62 cm^3^, and 12.7 cm^3^ enabled nematode migration to depths of 30 cm, 45.6 cm, and beyond 45.6 cm, respectively. Nematode population distribution closely tracked root growth, which reached 76 cm by 60 DAP. Although vermiform stages were detected at 76 cm by 45 DAP, colonization of roots at this depth was not observed until 90 DAP. These findings demonstrate that *R. reniformis* migrates both vertically and horizontally in response to cotton root presence, rapidly establishing persistent populations throughout the soil profile once introduced.

## Characterization of a novel stylet-secreted effector protein in Root-Knot Nematode parasitism

Lee, Rebekah^1^, R. S. Hussey^1^, R. O. Rocha^2^ and M. G. Mitchum^1^

^1^Department of Plant Pathology and Institute of Plant Breeding, Genetics, and Genomics, University of Georgia, Athens, GA, 30602

^2^The Department of Plant Pathology and Ecology, Connecticut Agricultural Experiment Station, New Haven, CT, 06511

### Abstract

Root-knot nematodes (RKN, *Meloidogyne* spp.) are sedentary endoparasites that are considered the most important group of plant-parasitic nematodes in agriculture due to their wide host range and worldwide distribution. Current management strategies include nematicides and resistant cultivars. However, these strategies have disadvantages such as toxicity to the environment and to humans while repeated use of resistant cultivars can lead to virulent nematode populations. Identifying vulnerable points of disruption in the RKN life cycle can aid in the design of novel resistance strategies. All species of RKN are successful in parasitizing host plants by releasing stylet-secreted effector proteins produced in three esophageal gland cells, two sub-ventral and one dorsal, that alter host cell structure and function leading to the formation of giant feeding cells. Our previous study coupling the isolation of the highly active dorsal gland (DG) from adult females of *M. incognita* (*Minc*) with RNA sequencing identified more than a dozen *Minc* genes coding for novel candidate effector proteins shown to be upregulated exclusively in the DG of adult females by *in situ* hybridization. These effector proteins had no known function or homology to proteins in any other organisms, suggesting that these proteins may be playing critical roles in later stages of nematode parasitism. However, there is no direct evidence of these effector proteins being secreted by adult females into host root tissues. To address this gap in knowledge, a recombinant protein was used to produce a polyclonal antibody against one of these novel effectors. Immunolocalization studies on adult female specimens showed the effector protein is produced in the DG and packaged into secretory granules that collected in the ampulla, providing strong evidence for stylet secretion. *In planta* immunolocalization is being used to verify secretion of the effector protein into giant-cells and determine subcellular localization to garner additional functional insights. Identifying and understanding how this effector protein functions in parasitism can provide new targets to exploit for enhancing RKN resistance in crop plants.

## Inter-regional composition of nematode fauna in two pine trees (*Pinus densiflora* and *P. Thunbergii*) killed by the pinewood nematode, *Bursaphelenchus xylophilus* in Korea

Lee Yeongdon^1^, A. O. Mwamula^2^, D. G. Lee^3^, Y. S. Kim^2^, T. W. Han^1^, H. L. Kim^4^ and D. W. Lee^2,3^

^1^Hallasan Research Department, World Heritage Office, Jeju Special Self-Governing Province, Jeju. 63143, Republic of Korea

^2^Research Institute of Invertebrate Vector, Kyungpook National University, Sangju 37224, Republic of Korea

^3^Department of Ecological Science, Kyungpook National University, Sangju, 37224, Republic of Korea

^4^Forest and Green Division, Jeju Special Self-Governing Province, Jeju. 63132, Republic of Korea

### Abstract

The pinewood nematodes (PWN), *Bursaphelenchus xylophilus* was first reported in Korea in 1988. Since then, its damage to pine wood tree stands continues to spread within the country. *Pinus densiflora*, *P. thunbergii* and *P. koraiensis* are the main species of trees that are affected by PWN in natural forests. This study was conducted in natural forests of black pine (*P. thunbergii*) and red pine (*P. densiflora*) trees to determine regional differences in nematode species composition and populations in PWN-infected dead trees. Thirteen and twelve species of mainly fungal feeding insect-associated nematode group were identified in PWN-infected dead black pine and red pine, respectively. Ten of the identified species were commonly recovered in both tree species. *Deladenus posteroporus* and *Mesodorylaimus* sp.1 were found only in red pine, and *Geraldius jejuensis*, *Poikilolaimus oxycercus*, and *Prionchulus* sp. were found only in black pine. The Simpson dominance index and Shannon-Wiener index showed statistically significant differences among regions in red pine trees. The densities of other nematode species and PWNs varied between regions in both tree species, and the densities of other nematode species showed an inverse correlation to PWN populations in PWN-infected dead trees. These groups of nematodes play an essential role in the dynamics of the fauna proliferating in PWN-infected trees.

## Fighting SCN with one voice: lessons from a groundbreaking public-private partnership

Lopez-Nicora, Horacio D

The Ohio State University, Department of Plant Pathology, Columbus, OH 43210

### Abstract

Soybean cyst nematode (SCN), *Heterodera glycines*, continues to rank as the most economically damaging pathogen of soybean in North America, with annual losses exceeding USD 1.5 billion. Despite decades of research, SCN persists and adapts, challenging conventional management approaches. In response, The SCN Coalition – a national public-private partnership – mobilizes researchers, extension professionals, and industry stakeholders to deliver unified, evidence-based recommendations. Since its relaunch in 2018, the Coalition has driven a 35% increase in SCN soil sampling and expanded the adoption of integrated management strategies, including crop rotation and resistant cultivars. These outcomes reflect the impact of coordinated communication and collaborative outreach on grower behavior and diagnostic engagement. The model established by The SCN Coalition has informed parallel efforts in education and applied research. The GrowNextGen Ag Biotech Graduate Academy, supported by the Ohio Soybean Council and private partners, empowers high school science teachers to become ambassadors of agricultural science, equipping them with the tools and knowledge to address soybean production threats, including SCN and other plant-parasitic nematodes. Through immersive, hands-on training in nematode extraction, soil sampling protocols, and inquiry-based instruction, the program enables educators to bring real-world agricultural challenges into the classroom. Over the past four years, more than 100 teachers have participated, extending soybean pathology and nematology literacy to thousands of students, many of whom represent the next generation of scientists. In the field, the eFields program at Ohio State University integrates on-farm research with extension delivery. Collaborative trials with growers and Extension educators generate region-specific data on SCN population dynamics, virulence phenotypes, and management efficacy. These findings feed directly into extension recommendations, enabling adaptive responses and reinforcing the feedback loop between research and practice. Together, these initiatives demonstrate the value of integrated frameworks that connect research, education, and extension. While GrowNextGen and eFields operate independently of The SCN Coalition, all three initiatives share a commitment to stakeholder-driven knowledge transfer. Their combined efforts generate the data, capacity, and engagement necessary to support sustainable nematode management and inform future policy development. The SCN Coalition’s model offers a replicable framework, adaptable across diverse agricultural systems, for building resilient, collaborative responses to persistent and emerging nematode threats. Fighting SCN with one voice remains the central lesson of this groundbreaking public-private partnership.

## Atroforce™: new biological nematicides for the control of plant parasitic nematodes in cotton

Ludwig, Scott, S. Sopher and N. Kumar

UPL NA Inc, Research Triangle Park, NC 27709

### Abstract

AtroForce™ is a biological nematicide containing *Trichoderma atroviride* K5 (NRRL B-50520) being developed by UPL as a seed treatment and in-soil applications for the control of plant parasitic nematodes. This new strain colonizes roots in three days providing excellent rhizospheric growth. Metabolites produced inhibit egg hatch and paralyzes juvenile life stages. In addition to providing a unique mode of action for management of plant parasitic nematodes, root biomass is also increased via below ground bio-stimulation. *Trichoderma atroviride* K5 can germinate at soil temperatures between 4–32°C with the optimum growth around 25°C. AtroForce provides a unique mode of action for management of plant parasitic nematodes.

## Potato Cyst Nematodes (PCNS), *Globodera pallida* and *G. Rostochiensis,* a brief overview of research work in Canada

Madani Mehrdad^1^ and S. De Boer^2^

^1^Former postdoctoral fellow

^2^Former head of science section, CFIA, Charlottetown, PEI, Canada

### Abstract

Potato cyst nematodes (PCNs), *Globodera pallida* and *G. rostochiensis*, are regulated soil-borne pathogens that pose a serious threat to potato production worldwide. In Canada, *G. rostochiensis* was first reported in 1962 in Newfoundland and Labrador, followed by a detection in British Columbia (Vancouver Island) in 1965. *G. pallida* was later identified in Newfoundland and Labrador in 1977. In 2006, *G. rostochiensis* was detected in the province of Quebec in a poor-yielding field of table potato, however, this report had economic consequences as the export of seed potatoes to the United States was temporarily banned. *National call:* to address this issue, the Canadian Food Inspection Agency (CFIA) and Agriculture and Agri-Food Canada (AAFC) convened a nationwide meeting in Ottawa. For the next three years, the CFIA Charlottetown laboratory in Prince Edward Island (PEI) led soil sample analysis, which resulted in the completion of several research projects. *Detection, Diagnostics and Port of entry:* The high-throughput soil washing unit and Fenwick can system installed in Charlottetown lab allowed for efficient screening of thousands of soil samples. Collected cysts were screened out under a stereoscope. Several methods were developed for the precise detection, identification and discrimination of extracted cysts from soil particles and debris. The Internal transcribed spacer (ITS) was used to develop a diagnostic multiplex real-time PCR, and *HSp*90 gene for confirmator y diagnostic test for discrimination of three species of *G. pallida, G. rostochiensis*, and *G. tabacum*. These tests were optimized in the capillary gel electrophoresis system and using melting curve analysis of DNA. *Population Genetics Study:* Mitochondrial cytochrome b and ITS were utilized for the molecular characterization and phylogeny study of the Canadian population and to investigate potential entry pathways of nematodes to the country. The ribosomal intergenic spacer (IGS) was employed to evaluate its possible capability for distinguishing PCN genotypes and the populations. Between 2015-2019, research conducted mainly in Ontario and Quebec identified pathotype Ro1 and demonstrated population diversity through genetic SNP analysis. *Biology, Host study and Management:* Further insight into PCN’s biology was then provided by research conducted mainly in the province of Quebec on *G. rostochiensis* cysts related to eggs viability, life cycle, cultural methods for management, detection of cysts in piler dirt sampling, hatching, survival, RNA sequencing transcriptome analysis, and *in vitro* culturing. *Remarks:* Potatoes are the fifth largest widely grown crop in Canada, with 5.7 million metric tons and a value of 5.1 billion dollars. Potato cyst nematodes are among the most significant potato pests worldwide and pose a potential threat to potato production in Canada. Due to the nature of PCN biology and pathogenicity, infestation can cause high yield-reduction. It is crucial to use the Integrated Pest Management (IPM) approach, which places a strong emphasis on early detection and preventive control methods to stop infections from spreading to new area. This strategy focuses on supporting and educating Canadian potato growers and the industry about the importance of PCNs, as well as promoting field monitoring and soil sampling.

## Noninvasive field scouting and monitoring, early detection approached for plant disease and nematodes

Madani, Mehrdad^1^, Y. Long^2^, P. Westdal^3^ and Ch. Bunio^3^

^1^Formerly agronomist at

^2^Ldon Technologies

^3^TheoryMesh Ltd, Winnipeg, MB. Canada

### Abstract

Integrated Pest Management (IPM) strategy is essential for food security and sustainable agriculture. In this context, aerial data collection via drones offers a promising alternative for field scouting and early detection of diseases particularly in mega farms. We employed advanced DJI Mavic 3 Multispectral (M3M) and Matrice 30 Thermal (M30T) drones equipped with thermal and zoom camera for taking high resolution RGB (Red-Green-Blue) and Near Infrared (NIR) photos. Our hypothesis was to assess the effectiveness of drone technology for identifying nematode damage in specific and, in general biotic/abiotic anomalies in the field. Experimental fields were set up in southern Manitoba during the 2023 growing season on three selected wheat, soybean, and canola fields. Flight missions (50 m altitude/speed of 5m/s) were conducted over entire field or selected strips (200×3 m). Ground photos were also taken by handheld camera from patches and plants having symptoms. To assess the accuracy of size and thermal gradient measurement, 60×180 cm wooden boards with attached soybean leaves exhibiting varying percentages of necrosis and chlorosis were placed in an experimental field. A hovering drone then captured images from altitude of 5,10, and 15 m. The preliminary results in this study proved the usefulness of the drones used for collecting imagery data where scouting and data collection took 24 minutes on average for covering 26437.4 m^2^ presenting efficiency of drone. More than 5000 photos acquired by M30T in NIR and R-JPG format with 640×512@30Hz, (focal length 40mm), and with M3M with multispectral RGB photos (½” CMOS 48 MP, 5-16 Optical Zoom, 200x Digital Zoom, 8K image, 4K video of Zoom camera). DJI Thermal Analysis Tool 3 software was used to process data. Photos in format of RGB, NDVI (Normalized Difference Vegetation Index), GNDVI (Green vegetation Index) and OSAVI (Optimized Soil-Adjusted Vegetation Index) and with palettes such as IronRed and Tint thermal, were created using the same software. Results revealed distinct margins between vegetative greenish parts, and with area having anomalies. In a particular wheat field, three sections are identified when data analyzed with OSAVI index. For the test conducted using wooden boards the photos taken at 5 and 10 m altitude allowed the discrimination between healthy green (22 °C) and necrotic (27.7 °C) leaves, and measured dimensions accurately. Ground photos confirmed symptoms such as blight, wilt, damping off, chlorosis, and stunting. Different background color, intensity and NIR reflectance in aerial imagery could potentially be due to biotic or abiotic stress, diseases and pests including nematodes and nutrient deficiency that were in accordance with the outcome of analysis of the aerial imagery. Drones play a crucial role in precision agriculture by providing a non-invasive, time-efficient, and resource-saving method for data collection. Further research required through soil testing for pathogens and nutrients to precisely validate aerial imagery, NDVI and thermal patterns with specific symptoms/pathogens, including soil-borne and foliar nematodes.

## Simultaneous co-infestation of carrot roots with root-knot (*Meloidogyne*) and cyst nematodes (*Heterodera*) in Ontario, Canada

Madani, Mehrdad^1^, M. R. McDonald^2^ and M. Tenuta^3^

^1^Formerly research associate nematology

^2^University of Guelph. Dept. of Plant Agriculture, Guelph, ON

^3^University of Manitoba, Soil Science Dep. Winnipeg, Canada

### Abstract

Ontario is a leading producer of carrot in Canada, with almost $50 million annual value. Three samples of carrot roots originally collected from separate fields in Ontario were analyzed at the Soil Science Department of the University of Manitoba. The taproots were stunted, stubby with excessive branching and hairy lateral roots. There were pimple-like bumps on the surface of the tap roots. Detailed examination under a stereomicroscope found brownish lemon-shaped cyst nematodes embedded in the hairy roots. White tiny protrusions on the surface of the carrot root were also observed, and further microscopic examination identified the white egg masses and posterior end of the female body characteristics of *Meloidogyne* species. Root-knot (RKN) and cyst nematodes were then isolated from roots and subjected to microscopic and molecular analyses for identification. The perineal pattern of white female of *Meloidogyne,* the vulval cone top structure of the posterior end of the cyst body and the J2 morphology of both species were analyzed for their main morphological characters using a compound microscope. Cyst vulval cone showed ambifenestrate structure with a ‘bean’ shape aperture and no bullae. The perineal patten of RKN showed a low upper arch, extended into lateral wings and the punctations in the tail region. Internal Transcribed Spacer (ITS1,5.8s and ITS2), D2-D3 expansion segments of the 28S rRNA gene, mitochondrial DNA (mt-DNA) and Ribosomal Intergenic Spacer (IGS) were PCR amplified and sequenced in both strands. The sequences of each gene were edited, assembled and subjected to similarity searched in the GenBank NCBI database and used to construct phylogenetic trees along with the sequences of the same species retrieved from GenBank. The results of the examination of the two isolated nematodes were consistent with the descriptions of the northern RKN, *Meloidogyne hapla*, and the carrot cyst nematode (CCN), *Heterodera carotae*, which was also verified by the outcome of the phylogenetic analysis and species-specific diagnostic PCR, and together with same time study conducted in CFIA Ottawa, make it the first report of the cyst in Canada. Co-infestation of the carrot root with RKN and CCN species synergizes the severity of the disease, symptoms, and crop loss. The fact that *M. hapla* has a wide host range which includes vegetables, greenhouse crops, and fruit trees, carrot growers should be particularly aware. Integrated Pest Management (IPM) strategies should account for the risk of coinfestation and target both nematode species effectively.

## Preliminary survey of Plant-Parasitic Nematodes in the sand dunes of Churchill bay, Canada

Madani, Mehrdad^1^ and M. Tenuta^2^

^1^Formerly research associate nematology

^2^University of Manitoba, Dep. of Soil Science, Winnipeg, MB R3T 2N2, Canada

### Abstract

Churchill Bay, where the town of Churchill is located in the northeast territory of the province of Manitoba along the southern edge of Canada’s Arctic on the west shore of Hudson Bay and Nunavut. The plant community in this area includes about 24 species, of which the genus *Elymus* (Poaceae), (Trin.), with two species of *E. arenarius* Sea-Lime Grass and *E. americanus* dune grass (also named *Leymus mollis*), are the dominant plant species in dune grass coastal beaches. From the floral community in the North subarctic of the Churchill area, *L. mollis* has been reported as a harbor for the migratory endo-parasite of *Pratylenchus* species with the abundance at 35,170 kg^−1^ individuals. Several composite samples of plant roots with the rhizosphere and bulk soil were taken from *L. mollis* and from several locations along the Churchill town shoreline to provide further details on the nematode fauna of this area. Examining roots under a stereoscope showed brownish, irregular lesions measuring 2 to 5 mm along the root cuticle, indicative of plant responses to nematode piercing and feeding. Above ground symptoms included necrotic lesions in the margins of the leaves and on the stems. After soaking roots and sand overnight in distilled water, emerged nematodes were examined for their morphology using a compound microscope. A high number of A diverse range of nematode species was observed, including at least three plant-parasitic Tylenchids species, each possessing distinct stylets. From individual nematodes, the Internal Transcribed Spacer (ITS; 747bp), the D2-D3 expansion segments of the 28S rDNA gene (808bp), and the 18S rDNA gene (1549 bp) were PCR-amplified and sequenced directly or after cloning. Microscopy, similarity search of the sequences using NCBI Blastn, and phylogeny analysis support the presence of the genera *Pratylenchoides*, *Pratylenchus,* and *Ditylenchus.* Species of Dorylaimids with high similarity to *Prodorylaimus* and *Epidorylaimus* were also observed. Up to 97% query cover and 94.9% identity were for the first top 10 accessions, highly supporting the presence of *Pratylenchoides* as a predominant species and *P. crenicauda* as a first hit. A lesser support was for *Pratylenchus* sp., *Ditylenchus* sp., and unknown nematode species. More detailed analysis is required to precisely determine the identity of the species. Our DNA sequence analysis did not establish a strong tie with *Pratylenchus dunensis*, which has been reported from the dune plants in Western Europe. Understanding nematode communities and species in the sand dune of Churchill Bay is important as their role in the dune ecological system and succession.

## Cactodera cyst nematodes: two new records for the prairie, Western and Atlantic regions, and two new records and one unknown species for Canada

Madani, Mehrdad^1^, M. Tenuta^2^ and P. Castillo^3^

^1^Formerly research associate nematology

^2^University of Manitoba, Dep. of Soil Science, Winnipeg, R3T 2N2, Canada

^3^Instituto de Agricultura Sostenible, Avenida Menéndez Pidal, 14004 Córdoba, Spain

### Abstract

Cyst-forming nematodes are sedentary, soil-borne endoparasites that cause significant damage to a wide variety of crops across the globe. The six genera *Heterodera, Globodera*, *Cactodera, Dolichodera, Punctodera, and Afenestrata* are known as cyst-forming nematodes. This group of nematodes is a member of the family Heteroderidae, which comprise 18 genera. *Cactodera* spp., are primarily found on non-agricultural crops, are less economically significant than other species such as *Heterodera glycines* Ichinohe 1952, the soybean cyst nematode (SCN), which is the most economically damaging species. Morphologically, cyst nematodes are characterized by the thin cuticle area surrounding the vulva (fenestra) and are classified in two groups: circumfenestrate which have a single fenestra (e.g., *Cactodera* and *Globodera*), and ambifenestrate or bifenestrate, which have two semicircular fenestrate either connected (ambifenestrate) or separated (bifenestrate) as seen in *Heterodera* spp. In Canada information on the presence of circumfenestrate cyst nematodes is limited to a few reports, including *Dolichodera fluvialis* in Quebec, *C. weissi* in Ontario and Quebec and *Punctodera punctata* associated with wheat roots in Saskatchewan and Quebec. The association of *C. estonica* with the roots of *Polygonum aviculare* L. (Polygonaceae) has also been reported. In our study, a Dutch auger soil sampler was used to collect samples from the upper 60 cm of soil in 48 fields across the province of Manitoba, Canada. A total of 60 composite samples were washed through the modified Fenwick elutriation-flotation system for cyst extraction. On a microscope slide, a drop of warm glycerin gelatin (jelly) was used to mount the posterior cyst body cone tops for microscopy analysis. The entire ITS (Internal Transcribed Spacer) regions, including the ITS1, 5.8S, and ITS2 regions, and the D2-D3 expansion segments of the 28S rRNA gene, as the most widely used and reliable markers, were PCR amplified, sequenced and subjected to multiple sequence alignment with the corresponding gene sequences from various species including *Heterodera, Cactodera, Globodera, Punctodera, Betulodera, Meloidodera, Rhizonema, Atalodera, Cryphodera and Rotylenchulus* which retrieved from GenBank for comparative analysis. BLASTn sequence comparison revealed high similarity between the studied samples and known *C. torreyanae* and *C. milleri* accessions. It also showed less sequence coverage and similarity to *C. weissi*, *C. estonica*, and an unidentified *Cactodera* sp. deposited in GenBank. For one sample the BLASTn result which yielded a larger amplicon of 1,080 bp, showed 100% coverage and 95% similarity with *Globodera artemisiae*, and 95.4% similarity with 97% coverage to *G. pallida* and *G. rostochiensis*. From this list *C. torreyanae* and *C. milleri* are new reports to Canada, and *C. weissi* and *C. estonica* to the prairie as previously reported on Ontario. Additionally, one unidentified *Cactodera* species may represent a new species or record for Canada.

## Culturing a newly identified saline nematode found in the Great Salt Lake

Marcue, Morgan C.^1^, Jung, J.^1,2^, Murray, T.^1^ and Werner, M.^1^

^1^University of Utah, Dept. of Biology, Salt Lake City, UT 84112

^2^Weber State University, Dept. of Zoology, Ogden, UT 84408

### Abstract

The Great Salt Lake (GSL) in Northern Utah is known for its extreme environmental conditions of high salinity, pink watercolor in the North arm due to archaea, and as a major stopover for millions of migratory birds. Previously, the only known animals in the GSL were brine flies and brine shrimp. Recently, the Werner lab found nematodes in the benthic zone of the GSL. Observing morphology and conducting 18S sequencing, these nematodes were identified in the genus *Diplolaimoides* (family Monhysteridae). These worms are abundant in diverse aquatic environments, including coastal, fresh, brackish waters, and in the extreme abyssal zone of the deep sea near hydrothermal vents. Given their ability to colonize practically all freshwater and marine environments, the Monhysteridae family represents a fascinating group of Metazoans. However, there is a lack of aquatic model nematodes and extremophile model animals in science, and we intend to develop the GSL nematode to fill this niche. Culturing the GSL nematode is an essential first step towards building a model system. To identify which culturing conditions these GSL nematodes need to survive and reproduce, this study investigates three different culturing agar conditions with two treatment types. Here we demonstrate that one condition provided more than 6 months of culturing viability. Using this culturing method has already provided vital information for species description (manuscript submitted). Culturing this GSL nematode has provided crucial information for species description. It will continue supporting the development of the GSL nematode as an aquatic, extremophile model organism and offer a long lineage of genetically similar worms for whole genome sequencing. Moreover, culturing will allow the opportunity to conduct physiological experiments to identify how these nematodes survive in the GSL, which has high and changing salinity. Studying this group can reveal the evolutionary history of aquatic nematodes and the process of animal adaptation to extreme environments.

## Long-term maintenance of systems with reduced management intensity enhances nematode communities and drives carbon accumulation

Martin, Tvisha

Department of Plant, Soil, and Microbial Sciences, Michigan State University, MI

### Abstract

There is an expectation that soil health promoting practices will enhance soil food web structure and increase soil carbon (C), yet this has rarely been tested over long-term periods. Here, we seek to understand how nematode communities and soil C indicators shift over a 30-year period across a range of agroecosystems within the W. K. Kellogg Biological Station Long-Term Ecological Research Site located in Michigan, USA. The study examines eight systems along a management intensity gradient with varying soil health practices. These include four annual row-crop rotations (corn-soybean-wheat) differing in tillage, fertilizer use, and cover crops; two perennial monocultures (Poplar, Switchgrass); and two unmanaged polycultures (early successional community, mown grassland). Soils were sampled in 1991 and 2021, and nematode communities were extracted and identified using identical techniques for each year. Soil health indicators of permanganate oxidizable carbon (POXC) and mineralizable carbon (MinC) were measured for each year and system. After 30 years, nematode communities shifted from bacterivore and plant parasitic dominance to fungivore dominance, in unmanaged successional systems. Both POXC and MinC values were significantly greater after 30 years, but only in the mown grassland systems. By 2021, MinC was significantly correlated with nematode communities in early successional and mown grasslands and POXC, was significantly correlated with nematode communities in early successional and poplar systems. Together, this decadal study demonstrates that the long-term maintenance of soil health promoting practices can alter soil food web structure and increase soil C indicators in agroecosystems.

## Phytonematicides of essential oils on mortality viability and egg-hatch ability of Root-Knot nematode J2 larvae *in vitro* assays

Mbokota Candy Khosa^1^, T. A. Mokoka^2^, J. Senabe^3^, S. O. Amoo^4^, P. W. Mashela^5^ and L. J. McGaw^2^

^1^ARC-Tropical and Subtropical Crops, Private Bag X11208, Mbombela 1200, South Africa

^2^Phytomedicine Programme, Department of Paraclinical Sciences, Faculty of Veterinary Science, University of Pretoria, Onderstepoort, 0110, South Africa

^3^Department of Biotechnology and Food Technology, Faculty of Science, University of Johannesburg, Doornfontein Campus, P. O. Box 17011, Doornfontein Campus, Gauteng, 2028, South Africa

^4^ARC-Vegetable, Industrial and Medicinal Plants, Private Bag x 293, Pretoria 0001, South Africa

^5^Green Biotechnologies Research Centre of Excellence, University of Limpopo, Private Bag X1106, Sovenga, 0727, South Africa

### Abstract

Root-knot nematodes infection is frequently reported from emerging-smallholder and commercial farming areas and is a major constraint for vegetable crops in South Africa. Alternative low-input, cost-effective and environmentally friendly nematode management strategies need to be developed to assist farmers with techniques to regain and maintain acceptable levels of food production. The effect of a 100 µl (10 %) dilution of each of nine essential oil (EO) extracts *viz*. *Rosmarinus officinalis*, *Pelargonium graveolens*, *Thymus vulgaris*, *Lippia javanica, Zingiber officinale*, *Origanum majorana, Lavandula spica*, *Eucalyptus obliqua*. and *Cinnamomum verum* on the mortality and egg-hatch ability of the J2 nematode larvae was tested in 96-well plates. A 100 µl suspension of distilled water containing 100 ±20 freshly-collected J2 or eggs of *Meloidogyne incognita, M. enterolobii* or *M. javanica* was added to each well containing 100 µl of diluted EO extract (i.e., a concentration of 8 mg ml^−1^ or 8 000 ppm). The treatments were assigned in triplicate to the wells in a completely randomized design with three wells representing replications per treatment. Three controls were included in the assays: a solution of Tween-80 and distilled water, 10 % MeOH/Tween-80 (0.1%) in distilled water and 1 000 ppm salicylic acid dissolved in 10 % MeOH/Tween-80 (0.1%) in distilled water. The mortality and egg-hatch assays were terminated after 24-, 48-, 72- h and 7-, 14- and 21- day exposure times, respectively. Both assays were repeated once. The results indicate that *R. officinalis* and *P. graveolens* extracts did not influence the J2 mortality of *M. incognita* at all concentration levels (8 000-4 ppm) after 24- to 72- h exposure times. *Thymus vulgaris*, *L. javanica, Z. officinale*, *O. majorana, Lavandula spica*, *E. obliqua* and *C. verum* significantly increased the mortality of *M. incognita* between 8 000-125 ppm after 48- to 72- h exposure times. All EO extracts had good activity between 8 000-125 ppm on J2 mortality of *M. enterolobii* and *M. javanica*. The superior efficacy of the EO extracts in comparison to the positive control salicylic acid provides further evidence of the potential usefulness of these EOs in the management of plant-parasitic nematodes.

## The hidden cost of virulence: fitness trade-offs in soybean cyst nematode adaptation to broad-spectrum resistance

Mekidani, Jacob Salu^1^ and M. G. Mitchum^1,2^

^1^University of Georgia, Dept. of Plant Pathology, Athens, GA 30602

^2^University of Georgia, Institute of Plant Breeding, Genetics and Genomics, Athens, GA 30602

### Abstract

The evolution of virulence in plant pathogens is often constrained by fitness trade-offs, yet these costs remain poorly understood in soybean cyst nematode (SCN, *Heterodera glycines*). This study investigated the fitness dynamics of virulent SCN populations capable of overcoming broad-spectrum host resistance found in the soybean plant introduction PI437654. A long-term greenhouse selection experiment tracking shifts in virulence revealed a decline in individuals virulent on the broad-spectrum resistant source when host selection pressure was removed, suggesting potential fitness trade-offs. Comparative reproductive assessments showed that SCN populations adapted to broad-spectrum resistance exhibited lower reproductive success on a susceptible cultivar compared to unadapted populations. Despite similar host penetration rates, post-penetration fitness costs were evident, including delayed development and reduced fecundity of females, likely due to changes in resource allocation necessary for maintaining virulence. These findings highlight the evolutionary constraints associated with SCN virulence, underscoring the importance of understanding fitness costs for resistance genes deployment in soybean production.

## Status of plant parasitic nematode distribution on soybean in Mississippi, USA

Mendoza, Aldwin, C. Liu and C. J. Balbalian

Mississippi State University, Department of Agricultural Science and Plant Protection, Mississippi State, MS 39762

### Abstract

Mississippi ranks 12^th^ in soybean production in the United States, yielding approximately 127.7 million bushels across 2.3 million acres. However, plant parasitic nematodes pose a significant threat on soybean production, causing substantial yield losses and reducing the soybean supply for both local and global markets. In northern Mississippi, soybean farms are often rotated with cotton, corn, and other row and cover crops. Identifying the specific nematode present in a field is crucial for growers to implement effective control strategies. This study analyzed five years (2020–2024) of data from 2,118 soybean field soil samples from farms across 34 counties in Mississippi. Samples were obtained from two primary sources: submission to Mississippi State University Plant Disease and Nematode Diagnostic Laboratory and field collections conducted by project personnel. This study reports the total number of samples analyzed, percentage of samples containing plant parasitic nematodes, and proportion of samples exceeding economic threshold levels (EDT) (nematodes per 100 cm^3^ of soil). Twelve genera of plant-parasitic nematodes were identified, including *Heterodera glycines*, *Meloidogyne* spp., *Rotylenchulus reniformis*, *Helicotylenchus* spp., *Hoplolaimus* spp., *Tylenchorynchus* spp., *Pratylenchus* spp., *Paratrichodorus* spp., *Xiphinema* spp., *Mesocriconema* spp., *Hemicycliophora* spp., and *Belonolaimus* spp. The distribution of these nematodes was mapped across soybean farms in Mississippi. Among them, spiral nematode (*Helicotylenchus* spp.) was the most prevalent, followed by reniform nematode and soybean cyst nematode (SCN). However, an analysis of samples exceeding the EDT revealed that although root-knot nematodes (*Meloidogyne* spp.) were the least frequently observed, they exhibited the highest percentage above EDT, followed by soybean cyst and reniform nematode. In 2024, SCN-positive samples were set up for HG Typing to test SCN populations in Mississippi. This survey remains ongoing across soybean farms throughout Mississippi to further understand the nematode population profile and their impact on soybean production. The findings of this survey are expected to help Mississippi growers in developing more effective nematode management plans.

## Assessing sweetpotato cultivars and breeding lines for resistance to the reniform nematode

Miller, Timothy and T. Watson

Louisiana State University, Department of Plant Pathology and Crop Physiology, Baton Rouge, LA 70803

### Abstract

Sweetpotato (*Ipomoea batatas*) is an economically important specialty crop that is primarily grown in the southern United States. One emerging pest, the reniform nematode (*Rotylenchulus reniformis*), can cause 30–40% yield loss in heavily infested soil, yet its symptoms often resemble nutrient deficiency, making detection challenging. Current management relies on soil fumigants and nematicides, but resistant cultivars would provide a more sustainable solution. However, the status of commercial cultivars is unknown, with very few host resistance studies being published in the last 25 years. This study aims to establish a reliable screening protocol, assess commercial cultivars for susceptibility, and identify potential resistance within genetically diverse germplasm. A 70-day time point was determined as the optimum time of growth for sweetpotatoes inoculated with the reniform nematode because it allowed for high levels of reproduction. Due to a lack of obvious symptoms, the number of eggs per gram of root was used as the metric to grade cultivars for resistance to the reniform nematode. Screening included 13 commercial cultivars, 10 advanced breeding lines from the LSU AgCenter, and 35 plant introductions from the USDA Genetic Resource Information Network (GRIN). Preliminary findings indicate that all 13 commercial cultivars allowed for damaging levels of reproduction (>100 eggs/gram of root) with the widely grown ‘Orleans’ being among the most susceptible. Results from the LSU AgCenter lines and USDA-GRIN plant introductions will determine whether nematode resistance sources are accessible from other closely related cultivars. By establishing a standardized resistance screening method and identifying susceptibility in commercial cultivars, this research lays the groundwork for breeding reniform nematode-resistant sweetpotatoes, offering a sustainable alternative to chemical management.

## RNA-based biopesticides, do we have regulatory and social license to operate?

Mitter, Neena^1,2^, SN Gunasekara^3^, R Tardin-Coelho^4^, SJ Fletcher^1,2^, P Fidelman^3^ and P Ashworth^4^

^1^Charles Sturt University, Wagga Wagga, NSW, Australia

^2^Centre for Horticultural Science, Queensland Alliance for Agriculture and Food Innovation, The University of Queensland, St Lucia, QLD, Australia

^3^Centre for Policy Futures, University of Queensland, St Lucia, QLD, Australia

^4^Curtin Institute for Energy Transition, Curtin University, Bentley, WA, Australia

### Abstract

RNA-based biopesticides provide safe, sustainable, non-GM crop protection by using RNA interference (RNAi) to silence essential genes in target pests and pathogens. Unlike conventional chemical pesticides, they are highly target-specific, minimising environmental and human health impacts. Like many new technologies, they face adoption barriers; in addition to technical factors like cost-effectiveness and safety, broad regulatory approval and Social Licence to Operate (SLO) are essential elements. Globally, annual chemical pesticide usage is 3–4 billion kilograms since 2011, equivalent to US$49 billion in 2022. In contrast, progress on environmentally sustainable RNA-based biopesticides has been slow. The OECD has provided important guidance such as its 2020 recommendations on environmental risk assessment and a 2023 framework for human health risk assessment, but uptake remains limited, mainly due to regulatory challenges. These include outdated regulations, fragmented systems across countries, and a lack of clear guidelines for assessing environmental risks. New data specifications and testing protocols may be needed, including selecting representative non-target organisms for risk assessments. Some regulatory initiatives have gained momentum. In 2018, New Zealand EPA determined that non-transformative RNAi technologies fall outside the scope of the 1996 Hazardous Substances and New Organisms Act. In October 2019, sprayable RNAi-based products were classified as agricultural chemicals, with regulatory oversight assigned to the Australian Pesticides and Veterinary Medicines Authority. In 2023, Canada’s Pest Management Regulatory Agency approved large-scale field trials of a yeast-based dsRNA product. GreenLight Biosciences has been granted registration by the U.S. EPA for Calantha™, the first foliar RNA-based insecticide, a milestone in the adoption of this emerging technology. Broader concerns about RNAi technologies may influence public acceptance of RNA-based biopesticides and consequently impact their SLO. Understanding public perceptions and directing research to address these concerns is therefore key to developing SLO and implementing the product, alongside current research efforts. Public trust depends on factors such as safety for humans and the environment, cost, off-target effects, degradability, resistance, toxicity, and ethical or cultural values. Addressing those factors will help develop social science-informed strategies to support the responsible adoption of RNAi biopesticides, contributing to more sustainable agriculture, improved food safety, and enhanced productivity. Technology development, such as bioinformatics tools like our recently published dsRNAmax, helps meet these regulatory and social licence requirements. These tools are essential for designing RNAi-based biopesticides that are effective against diverse target populations and highly specific to minimise off-target risks. This capability supports regulatory data needs with *in silico* specificity evidence and builds public confidence by proactively addressing environmental concerns. Ultimately, the long-term integration of design technologies, consistent and rigorous regulatory oversight focused on demonstrated safety, and transparent engagement to build public trust is essential for realising the full potential of RNA-based biopesticides as a sustainable crop protection solution.

## Integrative identification and distribution of *Meloidogyne hispanica* (Nematoda: Meloidogynidae) in Florida, USA

Moore, Matthew R.^1^, J. A. Brito^2^, R. Xue^2^, S. Vau^2^, C. G. Roberts^1^, L. A. Combee^1^, J. C. Fulton^3^, C. A. Paez^4^, P. S. Soria^4^ and K. K. Dey^4^

^1^Florida Department of Agriculture and Consumer Services – Division of Plant Industry, Molecular Diagnostics Laboratory, Gainesville, FL 32608

^2^Florida Department of Agriculture and Consumer Services – Division of Plant Industry, Nematology Section, Gainesville, FL 2608

^3^Florida Department of Agriculture and Consumer Services – Division of Plant Industry, Honey Bee Diagnostics Laboratory, Gainesville, FL 32608

^4^Florida Department of Agriculture and Consumer Services – Division of Plant Industry, Plant Pathology Section, Gainesville, FL 32608

### Abstract

*Meloidogyne hispanica* was first reported in Florida in 2022 from *Caladium* × *hortulanum*, *Urena lobata*, and *Eupatorium capillifolium*. Since its initial detection, this root-knot nematode has been found sporadically in a few ornamental nurseries and a vegetable production setting. Accurate identification of this nematode species was obtained by qPCR assays, DNA sequencing, enzymatic and morphological analyses. Updated survey data based on molecular identifications indicate that *M. hispanica* occurs in nine Florida counties. Mitochondrial DNA sequence data revealed the presence of three variants of *M. hispanica* in Florida. One of these variants matches *M. hispanica* reported from Portugal. The other variants match those reported from South Carolina in 2021. A confounding feature of *M. hispanica* is its variable isozyme phenotypes (EST:MDH); in Florida there is evidence of a S2-M1:N3 population (from *Beta vulgaris*) and a A2:N1 population (from *Ulmus parvifolia* cv. Drake). These differ from the isozyme profiles in the original description of *M. hispanica* (S2-M1:N1).

## Nematode diversity from Coiba National Park, Panama: first results

Mundo-Ocampo M.^1,2^, S. Justo^2^, E. Diaz-Ferguson^2^, E. Garrido^2^, J. Beacham^3^ and P. De Ley^1,3^

^1^University of California Riverside, Nematology Department, Riverside CA 92521 USA

^2^Estación científica Coiba AIP, XCW9+MCW casa 145B, C. Gustavo Lara, Panamá, Panamá

^3^New México State University Las Cruces, Department of Entomology, Pl3ant Pathology and Weed Science, Las Cruces NM 88003, USA

### Abstract

The island of Coiba is the largest of the 39 islands off Panama’s southwestern coastline. Covering just over 500 km^2^, it is also the largest Central American island in the eastern Pacific. Coiba National Park was created in 1992 to protect the marine and terrestrial biodiversity of the local forest, mangrove, beach, and coral reef ecosystems. It received UNESCO’s World Heritage Site designation in 2005 and includes Central America’s largest remaining surface area of intact primary rainforest. From 1919 to 2004, the island served as a penal colony for several hundred prisoners; during these years, small coastal valleys on the main island were used for sustenance cropping and cattle raising. In 2024, we conducted a preliminary nematode survey to initiate a nematode biodiversity project on the island. Ten sites in the northwest region of the island were selected to assess the diversity of soil nematode communities. Soil substrates and intertidal sediments were processed for nematode extraction and preserved in a 5.0% formalin solution at the island research station. Identification of nematode taxa was performed at the Taxonomy Unit of the Nematology Program at New Mexico State University-Las Cruces and the University of California-Riverside. The specimens were processed into permanent mounts and identified via light microscopy and scanning electron microscopy. The preliminary results include a total of 41 nematode genera identified to date and suggest a probable introduction of the nematode genus (*Xiphinema*) from the continental mainland. The intertidal nematode diversity partially resembles the composition of communities reported from other coastal regions of the Pacific Ocean. Additional information and explanations of the findings will be provided in the poster presentation.

## Characterization of a new species of the rare genus, *Geraldius* (Nematoda: Chambersiellidae) from Korea

Mwamula, Abraham Okki^1^, C. H. Bae^2^, D. G. Lee^3^, Y. S. Kim^1^, Y. D. Lee^4^ and D. W. Lee^1,3^

^1^Research Institute of Invertebrate Vector, Kyungpook National University, Sangju 37224, Republic of Korea

^2^Biodiversity Research Department, Species Diversity Research Division, National Institute of Biological Resources, Incheon, 22689, Republic of Korea

^3^Department of Ecological Science, Kyungpook National University, Sangju, 37224, Republic of Korea

^4^Hallasan Research Department, World Heritage Office, Jeju Special Self-Governing Province, Jeju. 63143, Republic of Korea

### Abstract

Until recently, the genus *Geraldius* has been monotypic, with only one known species, i.e., *Geraldius bakeri*. Currently, three nominal species comprise the genus and all have only been reported from the Americas (North America and South America). During a nematological survey conducted in 2025 in pine forest ecosystems in the Republic of Korea, a population of an undescribed species belonging to *Geraldius* was recovered from the bark layer of dead pinewood nematode-infected black pine tree stand (*Pinus thunbergii*). The new species is characterized by its lateral fields with two incisures, lip region continuous with body, conoid to rounded, 9.5–11.5 μm in diameter, with six branched cirri, stoma 2.5–3.0 times as long as labial region diameter, sclerotized, clearly subdivided into three regions: cheilostom, gymnostom and stegostom, hemizonid and excretory pore located posterior to nerve ring, excretory pore opening just at the beginning of hemizonid or within the contour of hemizonid, vulva a transverse slit in ventral view, opening in a depression, giving a circular profile in lateral view; rectum, 1.4–1.7 times longer than anal body diameter, phasmids located 55.0–78.5 μm from anal opening, tail elongated, 146.0–177.0 μm long, gubernaculum 27.0–33.5 μm long, caudal papillae arrangement: seven pairs pre-cloacal, two adcloacal, and six post-cloacal pairs, and 3 additional midventral papillae on anterior cloacal lip. The caudal papillae arrangement in males, vulva shape and location, long tail and location of phasmids differentiate the new species from the three known species of the genus. The phylogenetic relationships among species were reconstructed using 18S-rRNA, and 28S-rRNA gene sequences. Inferences from both genes corroborate the close morphological relationships between *Geraldius* and *Diastolaimus*.

## Chemical composition, and nematicidal activity of *Kigelia africana* and *Zanthoxylum chalybeum* against the pine wood nematode, *Bursaphelenchus xylophilus*

Mwamula, Abraham Okki^1^, K. M. Jeon^2^, D. G. Lee^2^, Y. S. Kim^1^, Y. H. Choi^3^ and D. W. Lee^1,2^

^1^Research Institute of Invertebrate Vector, Kyungpook National University, Sangju 37224, Republic of Korea

^2^Department of Ecological Science

^3^Department of Ecology and Environmental Science Kyungpook National University, Sangju, 37224, Republic of Korea

### Abstract

The pine wood nematode, *Bursaphelenchus xylophilus*, is a well-documented devastating pathogen of economic importance causing pine wilt disease on susceptible pine tree varieties in Korea and other countries. In Korea, trunk injection of synthetic nematicides is the preferred method of control. In recent years, there have been attempts towards testing naturally occurring nematicidal compounds isolated from plants to limit and combat the effects of environmental pollution and health related problems associated with the use of the hazardous synthetic nematicides. In this study, the nematicidal activity of *Kigelia africana* and *Zanthoxylum chalybeum* and the chemical composition of their extracts were investigated. The phytochemical analysis of the extracts were analyzed by liquid chromatography–mass spectrometry. The most abundant metabolites in *K. africana* included methyl tetradecanoate, 1,4-benzenedicarboxylic acid, methyl stearate and hexadecenoic acid. In *Z. chalybeum,* 2-Tridecanone, methyl tetradecanoate, hexadecenoic acid and 1,4-benzenedicarboxylic acid were the abundant metabolites. The efficacy of the various extracts against the pine wood nematode were investigated through determining their sublethal toxicities. Nematodes were treated with varying concentrations of the extracts in multi-well culture plates, and rates of mortality were determined after 24 hours. The hexane and chloroform extracts of *K. africana* exhibited nematicidal activity against *B. xylophilus*, with EC_90/24h_ values of 39 and 32 ppm, respectively. The chloroform extracts of *Z. chalybeum* also showed high nematicidal activity against the pine wood nematode, with a EC_90/24h_ value of 187 ppm. These *in vitro* results suggest that extracts from especially *K. africana* may be good potential nematicides against *B. xylophilus*.

## Phylogenetic inference and the diversity of animal-parasitic nematodes

Nadler, Steve

Department of Entomology and Nematology, University of California, Davis, CA 95616

### Abstract

During the last 30 years molecular phylogenies have profoundly changed our understanding of nematode systematics and evolution, ranging from relationships among the deepest lineages to phylogeography within species. Animal-parasitic nematodes, particularly those with vertebrate hosts are well-represented in nematode phylogenies and have been the subject of many comparative evolutionary studies. Unsurprisingly, molecular phylogenies have revealed that parasitism has arisen repeatedly among nematodes, with several such events among parasites of animals. A result frequently reported from phylogenies of animal parasites is recurrent evolution (homoplasy) of various characters and traits. Molecular phylogenies have also upended many conventional views of nematode taxonomy, leading to some revisionary proposals. However, a comprehensive taxonomic revision for the phylum awaits better sampled and stable molecular phylogenetic hypotheses. Of perhaps greater immediate interest are phylogenies of animal parasites revealing recurrent evolution of features that were once believed to be strong evidence of close relationships. One such case is the evolution of tissue tropism or site specificity for parasites of vertebrates such as lungworms (Metastrongyloidea) or among the more taxonomically diverse parasites in clade III. Evolutionary trees have also been instrumental in discovering previously unrecognized (cryptic?) species among parasites. Such studies include both “molecular prospecting” for unrecognized species and multilocus species delimitation. An instructive example of applying molecular data to investigate unrecognized species diversity involves hookworm (*Uncinaria*) parasites of pinniped hosts. Emerging advances in genomics and analytical approaches in comparative phylogenetics have the potential to refine understanding of the relationships and biodiversity of animal-parasitic nematodes.

## Soil nematode composition and ecosystem processes: comparing agricultural ecosystems with natural ecosystems

Neher, Deborah A and T. R. Weicht

Department of Agriculture, Landscape, and Environment, University of Vermont, VT 05405

### Abstract

Generalizing differences between agricultural and natural systems is challenging. An overarching theme is that physical disturbance is damaging to the soil food web both directly and indirectly. Cultivation destroys natural microhabitats in soil and homogenizes microbe and nematode communities. Physically intact soils maintain myriad microhabitats and niche partitioning for soil community assemblages that perform ecosystem functions such as nutrient cycling and decomposition. For example, there is a tighter coupling of nematode communities with mineralized nitrogen in 100-year pine forests than clear-cut forests or agricultural lands. Furthermore, long-term no-till soil high in organic matter is suppressive to soybean cyst nematode and subsequent cultivation destroys the suppressive community. In natural systems, litter and plant cover buffer soils from seasonal extremes in temperature and moisture. Physical disturbance changes water storage and infiltration. The spatio-temporal distribution of air and water films affect the ability of nematodes to survive, reproduce and disperse. Typically, moist soils in agriculture contain relative abundances of rhabditids and plectids. As agricultural soils dry, the biodiversity declines and nematodes capable of surviving dry soils increase in relative abundance including Aphelenchoididae, *Ditylenchus*, Cephalobidae and Aporcelaimidae. It is no surprise that the latter taxa are relatively abundant in desert soils. These taxa are capable of anhydrobiosis which increases survival in dry soils. This survival mechanism allows nematodes to survive temperatures of 60 C in desert soils without rainfall. Temperature decreases and soil humidity increases with depth in desert soils which accounts for the greater proportion of nematodes that are active. Furthermore, natural systems have greater vascular (e.g., grasses, trees) and/or nonvascular (e.g., moss, algae) plant diversity than agriculture. Contrasting plant species and community structure creates more niches to support additional biodiversity. Unfortunately, sampling protocols for agriculture focusing on soil overlook nematodes in litter accumulating on soil surface or epiphytes, underestimating nematode biodiversity and their role in ecosystem function. Nematode bioindicators are laborious and rely on specialized training. In an ideal world, we could predict nematode communities based on plant communities. Two case studies will be presented, one of temperate forests and the other of desert communities. For example, in a desert, nematode communities were consistently more diverse and successionally mature beneath relatively late- than early successional stage biocrusts. Ultimately, the utility of nematode community indicators is to reflect the ecosystem functions they perform. Temporally stable natural communities provide matrices of microhabitats that maximize biodiversity and linkages to ecosystem function.

## Nematodes on the move: management considerations

Noling, J.W

Citrus Research and Education Center, University of Florida, Lake Alfred, FL, United States

### Abstract

Nematodes are capable of movement along a variety of active and passive pathways, all of which being strongly influenced by a diversity of biotic and abiotic factors. This presentation will focus on management of plant-parasitic nematodes through an understanding of their microenvironment and of controls to regulate localized movement and of their geographical dissemination across field, State, and international boundaries. To address the depth distribution of nematodes in Florida, a vertical management zone approach, a prebedding treatment with a deep shank applicator is now used to first inject fumigants to a depth of 40 cm or more. This deep treatment is then immediately followed by an application of another separately applied fumigant into the raised plant bed during the bedding operation. The substantial yield increases using the vertical management zone approach strongly suggests that nematode damage potential to a given crop occurs from migrating individuals from soil depths after and below which fumigants distribute. There is sufficient data from field research to suggest that effective use of the post plant applied biological nematicides will require multiple applications, synchronized with host and nematode biology, to maintain efficacious concentrations in soil and or on plant foliage. The future of nematode management with the new biological nematicides continues to be strongly impacted by our lack of understanding of chemical movement and pest distributions in soil. This presentation will attempt to clarify recent findings of all kinds correlating efficacy with pest and chemical movement considering a variety of physical, chemical, and biological factors. Other measures used to control the movement of nematodes involve the implementation of nematode phytosanitary measures and certification programs that protect agricultural interests from the introduction and establishment of exotic pests within local, state, and federal boundaries. Unfortunately, these programs are only designed to certify that plant materials and field nurseries are free of nematodes that are of regulatory concern to other states and countries, but not necessarily free of other damaging nematode pests which may already be endemic.

## Biosolarization with grape pomace and different forms of winter cover crop termination methods for nematode pests and soil health management

Paudel, Roshan^1^, K.-H. Wang^1^, P. Vieira^2^, D. Joseph^3^, B. Waldo^2^ and C. R. R. Hooks^4^

^1^Department of Plant and Environmental Protection Sciences, University of Hawaii at Manoa

^2^USDA ARS, Beltsville, MD

^3^University of Maryland Extension

^4^Entomology Department, University of Maryland, College Park, MD

### Abstract

Use of red clover (RC) (*Trifolium pratense*) and rye (*Secale cereale*) winter cover crop mix followed by spring strip-tillage has been shown to suppress weeds and manipulate insects. However, RC is susceptible to many plant-parasitic nematodes (PPN). Objectives of this study were to evaluate the efficacy of using 1) grape pomace and solar heat for biosolarization (Sol) in managing PPN, and 2) strip-till cover cropping integration with Sol to mitigate soil health disturbance from Sol alone. Field trials were conducted at the University of Maryland Upper Marlboro Facility in Maryland in 2023 and 2024. Four treatments initially planted with RC and rye as winter cover crop were followed by 4 termination methods before vegetable planting: 1) rye was crimped and strip-tilled in the intra-row (i.e. vegetable planting row), and RC remained as living mulch (LM); 2) rye in the intra-row areas was crimped but left as surface mulch whereas RC remained as a LM (LM-NT), 3) the entire plot was mowed, RC regrew as LM, and grape pomace was applied to the intra-row areas followed by strip-tillage, transparent tarping and drip-irrigation (LM-Sol), and 4) RC+rye were mowed and rotovated followed by Sol. Half of each plot was planted with okra, and the other half with summer squash. Soil was collected soon after cover crop termination, at the beginning of crop harvest, and after harvest completion. Nematodes were extracted, counted, and subjected to nematode community analysis. Spiral (*Helicotylenchus*), lesion (*Pratylenchus*), stunt (*Tylenchorhynchus*) and lance (*Hoplolaimus*) nematodes were the most dominant PPN and were lowest in Sol in 2023 (*P* ≤ 0.05). In 2024, both Sol and LM-Sol lowered the number of *Tylenchorhynchus* than LM and LM-NT, but no differences were detected for the other PPNs. This suggests that the grape-pomace biosolarization was effective against PPNs. Soil health-wise, bacterivorous nematodes were enhanced by LM compared to Sol significantly in 2023 (*P* ≤ 0.05), but only numerically in 2024. Integrating LM with Sol (LM-Sol) mitigated the negative effects of Sol on bacterivorous nematodes in 2023 (*P* ≤ 0.05), though not significant in 2024. LM maintained a higher abundance of omnivorous nematodes and enrichment index (EI) than Sol at the end of 2023. However, this effect was not detected in 2024. Interestingly, LMSol and Sol increased nematode diversity compared to LM-NT in 2023 and 2024, respectively (*P*≤0.05). Using nematode metabolic footprint to depict soil health changes on the EI-SI trajectory, LM resulted in more nutrient-enriched conditions than Sol by season end in both trials. However, integrating LM with biosolarization (LM-Sol) slightly reduced the negative effect of Sol. Therefore, integration of strip-till cover cropping and grape-pomace biosolarization could improve PPN and soil health management practices.

## Convergent evolution of obligate symbiosis between bacteria and fly-parasitic Tylenchid nematodes

Steve Perlman, S. Albalkhi, S. Armstrong and M. N. J. Angeley

University of Victoria, Department of Biology and Centre for Forest Biology, Victoria, British Columbia, V8P 5C2, Canada

### Abstract

Symbiotic bacteria are a major source of adaptation and innovation for many groups of animals, yet they have been relatively little studied in nematodes. In this talk, we will provide an overview of our work on two lineages of *Howardula* (Tylenchida: Allantonematidae) nematodes that are parasites of flies and that have independently acquired obligate intracellular bacterial symbionts. The first harbours a newly discovered supergroup of *Wolbachia*, a diverse and widespread bacterial symbiont of terrestrial arthropods and filarial nematodes. This new *Wolbachia* has a highly reduced genome, with only 550 kilobase pairs of DNA, and has lost many genes, including entire pathways associated with cell division and cell wall synthesis. In contrast, the second lineage harbours a strain of *Symbiopectobacterium*, a recently described group of bacteria that is closely related to the plant pathogenic soft rot Enterobacteriaceae, and that includes free-living strains as well as many symbionts of insects. Unlike the *Wolbachia* nematode symbiont, the *Symbiopectobacterium* nematode symbiont has a ver y large genome that contains over a thousand pseudogenes, indicating a recent transition from a free-living microbe to a symbiont. Although the role of these symbionts is not yet known, we hypothesize that they provide their hosts with essential nutrients to support nematode growth and reproduction, as, interestingly, both symbiont-bearing nematode lineages have ver y large viviparous mother worms. To begin to test this hypothesis, we are performing controlled nutrient manipulation experiments and then measuring fly, nematode, and nematode symbiont fitness and gene expression.

## Optimization of microfiber and cellulose sponges for Entomopathogenic Nematode Formulation and storage

Pitiki, Melanie and B. Sipes

Department of Plant and Environmental Protection Sciences, University of Hawai‘i at Mānoa, Honolulu, HI 96822

### Abstract

Two of the most important aspects for a biological control agent, such as entomopathogenic nematodes (EPNs), to be successful is the ability to be mass-produced and formulated. Freshly harvested EPNs perish rapidly in water due to oxygen depletion, necessitating alternative storage methods to maintain their viability and infectivity. Studies have used substrates and carriers such as polyetherpolyurethane sponge and vermiculite for storage and transport of EPNs. This study evaluates the survival, recovery, and infectivity of *Steinernema feltiae* infective juveniles (IJs) formulated on cellulose and microfiber sponges and stored for 14, 30, or 60 days at 15°C. EPNs were added to a 1 cm^3^ square sponge or a filter paper control contained in a Petri dish. After storage, infectivity was assessed by exposing larvae of *Galleria mellonella* to the stored IJs and recording mortality. The experiment has been repeated twice and results from both experiments indicated that both the cellulose and microfiber sponges maintained IJs infectivity significantly better than the control. However, no significant difference was observed between the two sponge types, suggesting that either can be used for EPN formulation. Storage duration significantly affected infectivity, with IJs stored for 14 and 30 days providing greater larval mortality within 24 hrs compared to IJs stored for 60 days which showed delayed infectivity. No significant interaction was found between sponge type and storage duration, indicating that their effects on IJs infectivity are independent. These preliminary findings suggest that cellulose and microfiber sponges effectively preserve EPN viability for up to 30 days, while longer storage (60 days) reduces infectivity. Further optimization of formulation methods is necessary to enhance the long-term EPN storage for biocontrol applications.

## Limited impact of competitive interactions on nematode community assembly across habitat types

Porazinska Dorota L.^1^, K. Gattoni^1^, E. Gendron^1^, T. O. Powers^2^, C. J. Barnes^3^ and M. Vestergård^3^

^1^Department of Entomology and Nematology, University of Florida, FL 32611

^2^Department of Plant Pathology, 406 Plant Science, University of Nebraska-Lincoln, Lincoln NE 68583

^3^Department of Agroecology, Aarhus University, DK-4200, Denmark

### Abstract

Biotic interactions, such as competition and facilitation, are key deterministic processes in the assembly of biological communities and drive overall ecosystem processes. In belowground communities, biotic interactions occur among bacteria, fungi, and other eukaryotes within and across trophic levels of food webs. Through these interactions, organisms can regulate trophic relationships in soils and sediments. Although nematodes are a major component of belowground food webs, it is still unclear to what extent they interact with each other. Nematode-nematode interactions are hypothesized to shape their communities; however, documented evidence is limited. The most common documented interaction among nematodes is predation. Nematodes may also interact to communicate about food resource quantity and quality, overcrowding, or environmental stressors.

The Nebraska Sandhills is an ideal model ecosystem to study nematode interactions for several reasons. They support a diversity of habitat types, including distinct, naturally co-occurring lakes, shorelines, and prairies, all existing along an alkalinity gradient driven by concentrations of potassium, sodium, and chloride. Generally, the lakes support the lowest taxonomic and trophic diversity, followed by shorelines, then prairies. Additionally, the relative role of biotic factors (i.e., interactions) in shaping nematode communities is highly habitat specific. The goal of this study was to examine the potential for nematode-nematode interactions. Specifically, we expected that with the highest trophic and species diversity, the prairies would contain higher potential for competitive interactions, followed by shorelines, and then lakes, if at all. We collected lake, shoreline (sediment), and prairie (soil) samples from five lake basins along an alkalinity gradient (pH ~7–11) in the Western Nebraska Sandhills during mid-October in 2019, 2020, and 2021. We used 18S nematode metabarcoding to calculate nematode phylogenetic distances. First, we used Faith’s Phylogenetic Diversity (PD_Faith_) to compare nematode diversity among habitats and basins with generalized linear models. Second, we used the standard effect sizes of two metrics: 1. The mean nearest taxon distance and 2. The mean pairwise distance to identify patterns of potential competition via phylogenetic over-dispersion or facilitation via clustering of nematode communities as predicted by the null model. As expected, lakes supported nematode communities with the lowest PD_Faith_, and prairies with the highest. The over-dispersion was generally low across all habitats and lake basins, with lake communities experiencing virtually no over-dispersion regardless of phylogenetic distance metric and alkalinity. In contrast, shoreline communities showed the highest over-dispersion for both indices but only under the most alkaline conditions. Finally, prairie communities indicated moderate over-dispersion, but only for the mean pairwise distance. Overall, our results of low over-dispersion suggest minimal potential for competition and hence stronger facilitative interactions. This likely reflects strong environmental filtering, high resource and space availability, and overall reliance on diverse cross-domain interactions.

## Navigating the teaching terrain of Nematology in the USA: challenges and opportunities

Porazinska Dorota L^1^ and D. A. Neher^2^

^1^Department of Entomology and Nematology, University of Florida, FL 32611

^2^Department of Agriculture, Landscape, and Environment, University of Vermont, VT 05405

### Abstract

Nematodes, despite their fascinating attributes, remain underappreciated in the scientific community. These adaptable organisms thrive in every habitat on Earth, making them the most abundant and megadiverse metazoans. Positioned at various levels of the food web, nematodes contribute significantly to ecosystem functions including primary productivity, nutrient cycling, and biological control. Their versatility makes them excellent bioindicators of ecosystem health.

Despite these remarkable features, the field of nematology faces a shortage of specialists. To better understand our challenges and opportunities, we surveyed six individuals from six U.S. universities who teach nematology courses. Their responses highlight our current educational landscape and areas for improvement. Collectively, these institutions offer 24 courses, with Plant Nematology being the most common. Nematodes are often integrated into other courses, particularly Plant Pathology, with reduced coverage. Courses on plant-parasitic nematode (PPN) management and diagnostics are also common, while those focusing on basic aspects of morphology, taxonomy, or ecology are rare. Most courses emphasize PPNs. While many courses are still conducted in-person, there is a shift towards online courses, often missing practical training.

An alternative approach is to teach about nematodes in soil ecology courses, emphasizing their use as bioindicators of soil health. These courses are offered as a combination of in-person lectures and hands-on laboratory activities with specific core material devoted to nematode ecology. These courses can attract enrollment ranging from 10 to 33 students, both undergraduate and graduate, from a wide spectrum of disciplines including general biology, environmental sciences, forestry, natural resources, and agroecology. To provide a more detailed analysis of these trends, we will discuss two case studies: one from the University of Florida, and the other from the University of Vermont. Overall, challenges include the predominance of PPN-focused courses, small enrollments, and limited practical training. Online courses, while accessible, often lack hands-on experience. Resources such as live cultures, slides, and professional equipment are scarce, and educational materials are scattered. Opportunities lie in sharing online courses to boost enrollment, developing databases for slide collections, cultures, and images, and collating useful resources. Short courses could enhance practical training. By addressing these challenges and leveraging opportunities, we can advance nematology education and prepare future specialists to explore the fascinating world of nematodes.

## Development of a real-time quantitative PCR assay for rapid and direct detection and quantification of the root-lesion nematode, *Pratylenchus penetrans* from potato roots

Poudel, Dinesh and G. Yan

North Dakota State University, Department of Plant Pathology, Fargo, ND 58108

### Abstract

The root-lesion nematode, *Pratylenchus penetrans*, is an endo-parasitic nematode that causes significant yield losses in potato. Infection begins when the nematode penetrates the root tissue and migrates internally, causing necrotic lesions, stunted growth, and reduced tuber quality. Given its endo-migratory behavior, direct detection in potato roots is critical for effective management. Traditional identification and quantification methods are labor-intensive and time-consuming. Existing assays are based on nematodes in soil and tubers, highlighting the need for a direct, rapid, and root-specific detection method. Thus, the objective of this research was to develop and validate a SYBR-Green-based real-time quantitative PCR (qPCR) assay for rapid and direct detection and quantification of *P. penetrans* in infected potato root DNA extracts. Potential qPCR inhibitors in root DNA samples, such as polysaccharides, polyphenols, residual ethanol from DNA extraction, were identified using the pGEM-T Easy vector system. To neutralize inhibition, bovine serum albumin (BSA) was tested at four concentrations, with 0.4 μg/μl proving optimal. The specificity of the primer pair (PpF/PpR) targeting the D2-D3 expansion region of 28s rRNA of *P. penetrans*, originally developed in our lab for detecting *P. penetrans* in soil, was re-evaluated in potato root DNA extracts due to differences in sample composition and potential co-extracted organisms. The primer pair was specific, detecting only *P. penetrans* DNA among the co-extracted DNA samples of potato root and other control nematode species. The assay’s sensitivity was assessed by performing a two-fold serial dilution of DNA extracted from 0.2 g of root tissue inoculated with four *P. penetrans* individuals. The detection limit was as low as 1/64^th^ of a single nematode. A standard curve was generated by inoculating known nematode numbers into non-infected roots, yielding a strong linear relationship (*R*^2^=0.993) between quantification cycle (Cq) values and log-transformed nematode numbers, and high amplification efficiency (E=100.75%). Validation of standard curve with additional inoculation levels showed a high correlation (*R*^2^=0.988) between qPCR estimates and actual *P. penetrans* inoculated. The assay was tested in infected greenhouse root samples of five potato cultivars with varying level of resistance to *P. penetrans*. After 72 days of planting, *P. penetrans* numbers in roots were quantified using both qPCR assay and Whitehead tray extraction method followed by microscopic counting. Across all cultivars each in five replicates, qPCR and microscopic counts per gram of root showed consistent estimates. A strong correlation (*R*^2^=0.814) between qPCR estimates and microscopic counts confirmed the assay’s accuracy in detecting and quantifying *P. penetrans* in infected roots. The developed qPCR assay is specific and sensitive for detecting *P. penetrans* in potato roots, enabling rapid and direct quantification from infected root samples without prior nematode extraction. After further validation at different time points, this assay could speed up large-scale resistance evaluation at early stage, facilitating selection of resistant potato cultivars to manage this nematode pest.

## Exploring host resistance and associated Genomic regions in dry beans against a virulent soybean cyst nematode population of North Dakota

Poudyal, Prabhat^1^, J. F. Cerna^2^, J. M. Osorno^2^ and G. Yan^1^

^1^Dept. of Plant Pathology, North Dakota State University, Fargo, ND 58108

^2^Dept. of Plant Sciences, North Dakota State University, Fargo, ND 58108

### Abstract

Dry bean (*Phaseolus vulgaris*) is a nutritious and most popular grain legume crop consumed globally. North Dakota (ND) is the largest dry bean producer in the United States (US). However, the dry bean production is currently threatened by soybean cyst nematode (SCN; *Heterodera glycines*). The unique cropping system in ND, where dry bean and soybean (*Glycine max*) are sometimes alternated, increases the risk of SCN damage to two major crop industries. Several SCN populations have been previously characterized in ND, including, HG type 1.2.5.7. collected from Richland County, which is of major concern due to its higher virulence, and ability to overcome resistance from broader resistant soybean sources including Peking and PI 88788. In dry beans, SCN can result in a seed yield reduction of 56% depending on egg density in the soil, environment, and market class. Host resistance a suitable approach for SCN management. However, SCN-resistant dry bean cultivars are limited except for ‘ND Falcon’, known to be resistant to HG type 0, the most common SCN population in ND. Thus, the objective of this research was to screen 170 dry bean genotypes (113 breeding lines from the NDSU dry bean breeding program and 57 accessions from USDA-GRIN), for resistance to SCN HG 1.2.5.7 and conduct a Genome Wide Association Study (GWAS) to identify key genomic regions linked to SCN resistance. The genotypes comprised of popular market classes: black, navy, and slow darkening pinto from the Middle American gene pool, and dark and light red kidney from the Andean gene pool. Each genotype with four replications was inoculated with 2,000 SCN eggs and kept in the controlled growth chamber conditions for 30 days. Soybean ‘Barnes’ was employed as the susceptible check and Female Index (FI%) values were calculated for resistance response. Results showed that Middle American genotypes favored lesser SCN reproduction than Andean genotypes, a similar trend previously reported for SCN HG type 0. SCN reproduction was least on black beans followed by slow darkening pinto, navy, light red and dark red kidney. Based on FI, one genotype (PI 313733) was resistant, 35 were moderately resistant, 106 were moderately susceptible, and 28 were susceptible. For GWAS, the NDSU 3.8k BeanChip identified 2044 single nucleotide polymorphisms (SNPs) across 11 dry bean chromosomes. One significant SNP associated with nematode resistance was located each on Pv2, Pv7, and Pv11, marking a novel finding to previously untested SCN HG type 1.2.5.7. Potential candidate genes in the ±50kb region of significant SNP included R genes, transcription factors, and hydrolytic enzymes, all essential for host’s defense responses, nematode recognition and signaling. Further functional validation of these molecular markers and genomic regions will be conducted and marker-assisted selection will be utilized to accelerate the development of SCN-resistant dry bean cultivars.

## The cuticular ultrastructure of the Monhysterid *Theristus*: new insight from studies of *Caenorhabditis elegans*

Powers, Thomas^1^ and N. Schroeder^2^

^1^University of Nebraska-Lincoln, Dept. Plant Pathology, Lincoln, NE 68583

^2^University of Illinois, Champaign, IL 12345

### Abstract

The multilayered structure of nematode cuticle has been studied for over 135 years. Early investigations using light microscopy created a solid foundation for high resolution transmission electron microscopy (TEM) that followed in the 1960’s. A synthesis and limitation of the structural analyses of the nematode cuticle was well-described by A. F. Bird in his 1971 classic book, “The Structure of Nematodes.” Now the use of *C. elegans* as a model system has permitted extensive genetic insight into the function and development of the cuticular layers. A study of a marine species of *Theristus* (Nematoda: Monhysterida) conducted by the senior author in fulfilment of a MS degree, has been reexamined in light of new information derived from *C. elegans.* In the original study a single specimen of *Theristus* was found in which the outer cuticle layers were removed during critical point drying in preparation for scanning electron microscopy. What was revealed were the struts of the mesocuticle. The struts exhibited a regular arrangement of transverse rows of finger-like projections which underlie and flank the transverse striae of the cuticle surface. Pores appear beneath the struts suggesting the possibility of transport of fluids associated with mesocuticle function or formation. Transverse sections of *Theristus* cuticle by TEM show channels that might direct the flow of fluids. Investigations of struts in *C. elegans* have determined that three cuticle collagens BLI-1, BLI-2, and BLI-6 are critical in strut formation, and mutations in their genes are responsible for the Blistered phenotype. Whereas struts appear to be relatively rigid structures that connect the basal and cortical layers of the cuticle, the mesocuticle is primarily a fluid-filled layer. It has long been assumed that the struts provide a degree of structural integrity necessary for nematode movement. Research on mutant collagen genes in *C. elegans* suggest another role in the compartmentalization and transportation of lipids between cuticular layers.

## Paving the way for total control of PCN: PCN action Scotland’s programme for *G. Rostochiensis*

Price, James A and PCN Action Scotland

Cell & Molecular Sciences Department, The James Hutton Institute, Invergowrie, Dundee, DD2 5DA, UK

### Abstract

The UK is the 5th largest potato producer and exporter in Europe with an industry worth approximately £928 million at farmgate value and many billions in downstream industries. 77% of seed potatoes used in Great Britain originate from Scottish farms. However, this industry is under threat from potato cyst nematodes (PCN) which have been spreading across many UK potato growing areas for decades. Legislation in Scotland prevents seed potatoes from being grown on land where PCN have been detected. PCN are known to be present in almost 21,000 ha of Scottish soils. However, it is expected that approximately 35% (55,000 ha) of all Scottish potato land is infected with PCN. Current market varietal requirements and a lack of control options for growers mean that PCN is continuing to spread. Predictions suggest that PCN will cause the end of the Scottish seed potato industry by 2050, potentially only 4–5 rotations away.

Following a report in 2020, a Scottish Government PCN working group was initiated under the management of Scotland’s Plant Health Centre. This group, PCN Action Scotland, consisting of over 50 government, academic and industry partners are collectively working across 9 key work packages. In addition to core research, the group has proposed new policy changes, created new tools to help growers with PCN management and recommended incentives for improved PCN management to SASA. PCN Action Scotland is currently in its final year and this presentation will provide an overview of our work detailing how we anticipate managing total control of *G. rostochiensis* in Scotland. This framework will then be applied to *G. pallida* management ensuring the legacy of this enormous collaborative effort across the potato sector will increase the sectors’ capacity to manage the current potato cyst nematode epidemic and preserve the land base for future generations.

## *Gpa2*-induced selection pressure does not select for 187s allele of the effector *Gp-rbp-1* in virulent *Globodera pallida* populations

Putker, Vera^1,2^, N. Soffree^1^, D. M. te Molder^1^, S. J. S. van de Ruitenbeek^1^, C. C. van Schaik^1^, A. Goverse^1^ and M. G. Sterken^1^

^1^Wageningen University and Research, Laboratory of Nematology, Droevendaalsesteeg 1, Building 107, 6708 PB Wageningen, The Netherlands

^2^University of California, Davis, Siddique Lab, Department of Plant Pathology, 354 Hutchison Hall, 1 Shields Avenue, Davis, CA 95616, USA

### Abstract

Plant-parasitic nematodes pose a significant threat to global food-security, with the cyst nematode *Globodera pallida* being a major pathogen of potatoes. Management of *G. pallida* currently relies heavily on resistant potato cultivars, but these resistances have been repeatedly overcome by field populations. The interaction between the resistance gene *Gpa2* and its matching effector *Gprbp-1* has served as a valuable model in nematology for studying plant-nematode interactions, as it was hypothesised to represent a R/Avr pair. However, if *Gp-rbp-1* indeed contributes to (a)virulence on a pathosystem level, remains unknown. To understand the genetic dynamics at play in selection for virulence, we set out to test the role of *Gp-rbp-1* in virulence against *Gpa2*. Specifically, we hypothesised that the SNP encoding the P187S substitution in *Gp-rbp-1* correlates with (a)virulence of *G. pallida* on plants carrying the *Gpa2* resistance. To test this, we used the avirulent *G. pallida* population D383 and the partially virulent population Rookmaker in bulk segregant analyses of *in vitro* infections and pot experiments on the susceptible LineV and the transformant GPAII:Gpa2. Additionally, we conducted RNA sequencing to identify *Gp-rbp-1* transcripts and monitor the transcriptional responses in resistant and susceptible plants. Finally, we sequenced 20 historic populations with varying levels of virulence on *Gpa2*-carrying potato cultivars. Surprisingly, our genetic analyses refuted our hypothesis; the SNP encoding the P187S substitution in *Gp-rbp-1* is not responsible for virulence on *Gpa2*. Furthermore, we were able to establish that three *rbp-1* alleles segregate within the Rookmaker population, one of which contains a deletion of the signal peptide responsible for secretion that was not detected before. Together, our results open the search for the allele or alleles underlying virulence on *Gpa2*.

## OMICS enable accurate nematode taxonomy, phylogeny, and evolution

Qing, Xue^1^, W. Bert^2^, O. Holovachov^3^, D. Porazinska^4^, S. B. Miyara^5^, M. Y. Zhang^6^ and H. Li^1^

^1^Dept. of Plant Pathology, Nanjing Agricultural University, Nanjing, China

^2^Dept. of Biology, Ghent University, K. L. Ledeganckstraat 35, Ghent, Belgium

^3^Dept. of Zoology, Swedish Museum of Natural History, Stockholm, SE-10405, Sweden

^4^Dept. of Entomology and Nematology, University of Florida, Gainesville, FL 32611, USA

^5^Agricultural Research Organization (ARO), Volcani Center, Bet Dagan 50250, Israel

^6^Institute of Ecology and Evolution, University of Edinburgh, Edinburgh, EH9 3FL, UK

### Abstract

Historically, nematode taxonomy relied heavily on morphological characteristics. However, morphological identification is time-consuming, requires extensive training, and is often hindered by the lack of diagnostic features due to high phenotypic plasticity among species. Molecular barcoding and metabarcoding offer powerful alternatives for species identification and phylogenetic resolution, yet traditional pipelines face significant challenges when applied to nematodes. To address these limitations, we present four recently developed methodologies implemented in our laboratory: (1) Enhanced species identification tools. We developed PPNID, a bioinformatic program for identifying plant-parasitic nematodes within a phylogenetic framework, and for delineating root-knot nematodes using mitochondrial single-nucleotide polymorphisms (SNPs). Additionally, we created NemaRec, an image-based nematode identification tool, and the I-Nema database, both leveraging deep-learning algorithms for automated classification. (2) Metabarcoding and mitochondrial metagenomics for nematode community analysis. We designed degenerate COI primers for nematode metabarcoding, which demonstrate superior taxonomic coverage compared to existing primers. An automated mitochondrial metagenomics pipeline was developed, enabling high-resolution detection of rare or poorly characterized species while simultaneously assembling high-quality mitochondrial genomes. We also applied shotgun metagenomics to investigate nematode community functionality, demonstrating its utility through case studies. (3) Metagenomics for functional community profiling. By sequencing pooled nematode communities using both short- and long-read platforms, as well as RNAseq, we obtained metagenome-assembled genomes (MAGs), predict genes, and performed functional annotation of genes. Moving beyond taxonomy-based assessments, we evaluated community functional profiles by analyzing the composition and abundance of gene functional groups. (4) Phylogenomics and evolutionary insights. We sequenced 60 new nematode genomes spanning terrestrial and marine environments, representing eight orders within the phylum Nematoda—including the first genomic data for six orders. Using these genomes, we reconstructed the first comprehensive phylogenomic tree for Nematoda and estimated divergence times using molecular clock analysis. These integrative omics approaches significantly enhance the accuracy of nematode taxonomy, provide a more robust phylogenetic framework, and deepen our understanding of nematode evolution.

## A new species of *Longidorus* associated with intermediate wood fern (*dryopteris intermedia*) in Ontario, Canada

Qing, Yu^1^ and T. Sultana^2^

^1^Ottawa Research and Development Centre, Agriculture and Agri-Food Canada, Ottawa, Ontario, Canada

^2^London Research and Development Centre, Vineland Station, Agriculture and Agri-Food Canada, Vineland, Ontario, Canada

### Abstract

The genus *Longidorus* (Dorylaimida: Longidoriae), a remarkable group of nematodes of parasitic on numerous plant species including several agricultural crops and trees. Damage is caused by direct feeding on root cells as well as by transmitting nepoviruses that cause disease on those crops. A new species of *Longidorus* was discovered from rhizosphere of intermediate wood fern in southern Ontario. This new species is characterized by its guiding ring located 60 – 80 µm from the anterior end, and odontostyle with pseudo flanges in females. No males were found, it has 4 juvenile stages. This new species belongs to the *L. jonesi* species group, having the guide ring at mid-odontostyle area. Ribosomal DNA ITS, 18S, and 28S sequences are similar to those of morphologically similar *L. didecturus, L. jones, L. juglans* and *L. fangi*. This discovery will enhance our understanding of the diversity, morphology and evolution of the genus and the *L. jonesi* species group.

## The roles of transmission electron microscopy in comparative Nematology: past, present, and future

Ragsdale, Erik J

Department of Biology, Indiana University, Bloomington, IN 47405

### Abstract

The constancy of cell fates and numbers in some nematodes, including the widely studied species *Caenorhabditis elegans*, enables a comparative approach to anatomy at the level of individual cells. This feature has made it possible to study the molecular processes of animal development on a cell-by-cell basis and has provided a uniquely detailed view of how animal morphologies change over macroevolutionary time. In certain traits, the key tool for accessing the identities and relative positions of cells has been transmission electron microscopy (TEM). For over 50 years, TEM has proven its value in comparative nematode anatomy, revealing fundamental principles of how form can evolve. For example, studies reconstructing pharyngeal anatomy across several groups within the order Rhabditida have shown that, even across deep evolutionary timescales, the same number of cells has been conserved. This suggests that the resulting morphological differences—such as stylets, teeth, or other stomatal structures—arise not from changes in cell number, but from changes in the types of cells they become during development and their relative positions in the body. However, in other traits, TEM has revealed a contrasting pattern, such as in sensory dendrite (amphid) morphology, offering insight into how structure and function evolve over more recent timescales. Here, I offer a perspective on some of the principles that TEM has illuminated using nematodes as model organisms, and I highlight potential directions in which future research might leverage TEM to advance the field of comparative nematology.

## Effect of microbialite bacteria on salinity tolerance in Great Salt Lake nematodes

Rassoul-Agha, Maxim and M. Werner

University of Utah School of Biological Sciences, 257 S 1400 E, Rm 201, Salt Lake City, UT, 84103

### Abstract

Halophilic nematodes have been discovered in the Great Salt Lake (Jung et al., 2024). They were found to be associated with bacterial-built mounds called microbialite that line the shallow marginal areas of the lake; significantly higher numbers of nematodes were found living in microbialite sediment than in sediment adjacent to the microbialite. The mechanism of survival in hypersalinity remains unknown, but we hypothesize that cross-kingdom interactions between bacteria and nematodes may provide part of the answer. To test whether bacteria in GSL can increase saline tolerance of nematodes, we grew laboratory nematodes *Caenorhabditis elegans* (*C. elegans*) and *Pristionchus pacificus* (*P. pacificus*) on either 1) standard laboratory bacteria *E. coli* OP-50 or 2) microbialite bacteria. Worm survival 5 minutes and 24 hours after exposure to saline water was then measured. Nematodes raised on microbialite were observed to have increased survival in salinity up to 16% (>50x their normal salinity) over both time points. This suggests that bacteria from microbialites can provide salinity tolerance not only to Great Salt Lake nematodes but also to other species of nematodes. The reason for improved survivability is unknown and requires further investigation to understand how organisms can survive in extreme environments. We have isolated two distinct bacterial colonies that provide salinity tolerance to *C. elegans*. Ongoing work is being conducted to sequence their genomes and look for specific genes that can provide osmoregulation. Subsequently, the function of these candidate genes will be tested using knockouts or insertions into GSL bacteria and *E. coli,* respectively. Using a combination of laboratory and wild nematodes, and their associated bacteria, this research aims to discover the mechanisms behind the Great Salt Lake nematodes’ ability to survive in highly saline environments.

## Organic bloom hydroprotect^®^ for management of nematodes in soybean in the Brazilian Cerrado

Reis, Cristiane Bianchi Loureiro and A. S. Reis Junior^2^

^1^Ingal Agrotechnology, Santa Maria, RS, Brazil

^2^Franciscan University, Santa Maria, RS, Brazil

### Abstract

One of the biggest challenges for increasing productivity in soybean crops in Brazil is the presence of high population levels of nematodes. Nematodes are present in several biomes, from north to south of the country. Several strategies have been used together for integrated management aiming at returning the population balance of species in the soil. To this end, alternatives that are less problematic for the environment and aggressive to animals and humans have emerged. In this context, Organic Bloom HydroProtect® (HP), an organic compound based on phytic acid extracted from rice bran, amino acids extracted from soy protein and organic biopolymers obtained from linseed, is a tool that has been used to protect roots and promote soil health. During the 2024/25 soybean harvest, the use of Organic Bloom HP in the sowing furrow at a dose of 1 L/ha showed relevant results in reducing population levels of the main nematode species that attack soybean crops. The analyses were carried out in different locations, focusing on crops located in the Brazilian Cerrado, an environment highly susceptible to nematode reproduction due to favorable climatic conditions, highlighting the influence of temperature and precipitation. Nematodes of the species *Pratylenchus brachyurus* and *Helicotylenchus dihystera* were found in all analyses. In the seven farms monitored in the state of Goiás, there was a higher incidence of the species *Pratylenchus brachyurus*, in all samples the largest populations of this nematode were found associated with the roots. The reduction levels of nematodes of the species *Pratylenchus brachyurus* with the application of Organic Bloom HP (furrow - 1 L/ha) ranged from 21.38% to 89.62%, with an average reduction of 56.36%. It was also possible to observe the reduction of nematode eggs associated with the roots of soybean plants, which presented reduction levels that totaled values between 39.16% and 65.11%. The species that presented the highest levels in all analyses, secondarily to the lesion nematodes, were the nematodes considered emergent in soybean crops, of the species *Helicotylenchus dihystera*. The same were detected in all samples collected, but presented lower population levels, approximately 50% less, in relation to the nematode *Pratylenchus brachyurus.* The reduction percentages varied from 25.23% to 95.77% for *Helicotylenchus dihystera* in the sampled soybean roots. Regarding the eggs of *Helicotylenchus dihystera,* only two farms showed a reduction, with greater expression in the roots than in the soil, with levels ranging from 39.16% to 62.5%. The NDVI indices of soybean plants in control areas infested with a high index of *Pratylenchus brachyurus* and concomitant with the presence of *Heicotylenchus dihystera* were approximately 20% smaller than in areas treated with Organic Bloom HP up to approximately 60 days after sowing. These field results suggest that Organic Bloom HP has the potential to be included in management strategies for reducing nematodes in soybean crops.

## Discovery, characterization and implementation of genetic resistance resources to *Meloidogyne* and *Pratylenchus* in processing potato

Richael, Craig M. and S. Bali

Plant Sciences Division, J. R. Simplot Company, Boise, Idaho, USA

### Abstract

Currently, there is no genetic resistance to *Meloidogyne* and *Pratylenchus* species in popular potato (*Solanum tuberosum*) cultivars used for processed potato products. When a potato grower faces an infestation of these species, soil fumigation and the application of chemical nematicides represents a practical but expensive means to address the problem. Concerned about the rising costs and long-term availability of chemical control agents, the J. R. Simplot Company has recently embarked on the pursuit of genetic resistance for employment in processing potatoes by bioengineering. Results from the challenge of Cry protein transgenics with *Meloidogyne hapla* will be presented along with a plan to stack Cry proteins with other gene mechanisms for stable genetic resistance. The pursuit of nucleotide-binding and leucine rich repeat immune receptor (NLR) genes from a diversity panel of wild and semi-domesticated *Solanum* germplasm is underway. As the discovery, characterization and implementation of effective genetic resistance to more than one nematode species would benefit from an integrated process, the J. R. Simplot Company extends a hand of collaboration in this ambitious pursuit.

## Phenotypic evaluation of commercial potato varieties for resistance to Potato Cyst Nematodes populations from Bolivia

Sainz, Claudia^1^, C. Villaroel^2^, R. Silvestre^3^, I. Zasada^4^ and L-M Dandurand^1^

^1^Urbanización El Castillo D-42, Cochabamba, Bolivia

^2^Urbanización Magnolias III, Casa D-11, Cochabamba, Bolivia

^3^University of Idaho, Dept. of Entomology, Plant Pathology and Nematology, Moscow, ID 83844

^4^Oregon State University, Corvallis, OR 97331

### Abstract

The quarantine potato cyst nematodes (PCN), *Globodera pallida* and *Globodera rostochiensis*, are devastating plant-parasitic nematodes that threaten the potato production worldwide. In developing countries such as Bolivia, where potato is a staple crop vital for food security and farmer livelihoods, the impact of these nematodes is particularly severe. The Andes Mountain, specifically in southern Peru and northwestern Bolivia, is recognized as the center of origin and diversity for the cultivated potato. The probable origin of PCN in this region, suggested by their co-evolution with the potato host, has contributed to the presence of diverse and potentially virulent nematode populations. Identifying and utilizing sources of resistance within this center of diversity is important for developing future management strategies. This study focuses on the phenotypic evaluation of six commercial potato varieties which are widely cultivated and consumed in Bolivia, South America. These varieties were selected based on their popularity and relevance to local agricultural systems. The potato varieties are being tested for their resistance to three distinct PCN populations collected from potato-growing regions in Bolivia, previously identified as *G. pallida* and *G. rostochiensis* using molecular methods, to identify potential levels of host-plant resistance within commercially relevant germplasm. Discovering and deploying resistant varieties can significantly contribute to reducing nematode damage, improving yields for smallholder farmers, and enhancing long-term food security in developing countries as Bolivia. The phenotypic experiment was initiated between 2023–2024 and is currently in progress, with preliminary observations indicating interesting variations in host response among the tested varieties. Further evaluation will provide critical data on the resistance profiles of these important commercial potatoes against native Bolivian PCN populations. This information will be beneficial for breeding programs seeking to develop PCN potato varieties with durable resistance.

## *Solanum sisymbriifolium* root volatile effects on *Globodera Pallida*

Schulz, Lindsay and L. M. Dandurand

Department of Entomology, Plant Pathology and Nematology, University of Idaho, Moscow, ID 83844-2329

### Abstract

*Globodera pallida*, was first discovered in Idaho in 2006 and is an economically devastating pest of potato. The management goal for this pest is to contain and eradicate the infestation. Many nematicides used against this infestation are environmentally damaging and new environmentally friendly methods of control need to be developed. The plant *Solanum sisymbriifolium* is a trap crop for *G. pallida*, causing hatch but not allowing for reproduction, indicating a toxic effect. Previous research indicates that glycoalkaloids found in *S. sisymbriifolium* may be the cause of this toxic effect. Additionally, research has shown a 99% reduction in *G. pallida* on potato following *S. sisymbriifolium*. In Idaho this trap crop is not used by producers due to seeds being difficult to obtain and the plant not producing an economically viable crop. Due to these issues, producers do not utilize this trap crop in field. This research focuses on volatiles produced by *S. sisymbriifolium* that may be toxic to *G. pallida*. A hydroponics system with air forced from the roots of *S. sisymbriifolium* plants into magenta boxes with *G. pallida* cysts and 2:1 sand: soil mix was used. Potato root volatiles were used as a control. *Globodera pallida* cysts were exposed to volatiles for a week then viability and hatch assays were performed *in vitro*. Viability of *G. pallida* cysts was reduced by 27% compared to the potato control and hatch was reduced by 60% compared to the potato control. Identification of these volatiles is the next step in this research. This research indicates that *S. sisymbriifolium* volatiles are inhibitory or toxic to *G. pallida*. This discovery has the potential to be used as a natural source of fumigant nematicide to combat *G. pallida* infestations in Idaho.

## Plant Parasitic Nematode (PPN) C-terminally Encoded Peptide (CEP) Phytohormone mimics increase nitrogen efficiency in diverse crops

Schweiner, Abby, P. and P. DiGennaro

Department of Plant Pathology, University of Wisconsin-Madison, 1630 Linden Dr, Madison, WI 53706

### Abstract

Nitrogen efficiency and other nutrient uptake in agroecosystems across the globe are of economic and environmental importance, yet only 30–50% of applied nitrogen is utilized by crops, leading to substantial losses. While nitrogen management strategies optimize fertilizer applications, they often overlook biotic stressors such as plant-parasitic nematodes (PPN), which can influence nutrient uptake, including stimulating nitrogen absorption. Two genera of PPN--the root-knot nematode (RKN; *Meloidogyne* spp.) and the reniform nematode (*Rotylenchulus reniformis*)--secrete C-TERMINALLY ENCODED PEPTIDE (CEP) phytohormone mimics that hijack plant nitrogen signaling. Endogenously, these peptides are secreted from root to shoot, interact with a CEP receptor, and elicit a downstream signal which travels back to the roots and increases expression of nitrate transporters and enhances nitrogen absorption. Given the wide host range of these PPN, we screened the effects of eight nematode-encoded CEP mimics from six *Meloidogyne* species and one *Rotylenchulus* species on nine crops from seven plant families. Briefly, uniform seedlings were established in hydroponic culture for one week and then treated exogenously with synthetic peptide. One week after the addition of the peptide, leaf and root tissue were collected and analyzed for carbon/nitrogen ratio and gene transcription compared to non-peptide treated controls and plant-peptide treated controls. Additionally, we examined CEP receptor-ligand kinetics and performed molecular modeling to better understand the interactions between CEP and nitrogen uptake pathways. Together, these *in vitro* and *in silico* approaches highlight the potential of nematode-derived CEP mimics to increase nitrogen efficiency, reduce nitrogen inputs, minimize environmental runoff and leaching, and improve plant productivity, offering a novel strategy for sustainable nitrogen management in agriculture.

## Assessing nematicide mobility in a golf green soil profile

Scramoncin, Isabella, C. Green and W. T. Crow

University of Florida, Entomology and Nematology Dept., Gainesville, FL 32611

### Abstract

Plant-parasitic nematodes are important pests and pathogens of turfgrasses; on golf courses, the primary management tactic are nematicides. Unlike for most agriculture crops, the target treatment zone for turfgrass nematicides is very shallow, 2 to 10 cm deep, depending on the target nematode. Therefore, the relative mobility of the nematicide is often a key component of efficacy. This study aimed to compare the soil mobility of abamectin, fluopyram, fluensulfone, and fluazaindolazine in a golf course putting green environment. The experiment was conducted in two phases. First, nematicide treatments were applied to turfgrass plots on a golf green, then soil from the plots was collected at different time intervals to a depth of 15.25 cm into the soil profile. Soil samples were next segmented into six depth intervals for second phase bioassay testing. Results revealed that abamectin was the least mobile, followed by fluopyram, and that fluensulfone and fluazaindolazine were more mobile than other tested nematicides. These findings provide valuable insights into the mobility of turfgrass nematicides, informing best application strategies to optimize nematode management on golf turf.

## Co-operative Research Programme: Sustainable agriculture and Food Systems

Se-Yeoun, Cha

OECD, Jeonbuk National University, South Korea

### Abstract

The OECD Co-operative Research Programme (CRP): Sustainable Agricultural and Food Systems is a unique international initiative launched in 1978 to support evidence-based policy-making in agriculture, food, fisheries, and forestry. By funding international fellowships and conferences aligned with its three strategic research themes (1) Managing Natural Capital, (2) Strengthening Resilience in the Face of Multiple Risks, and (3) Transformational Technologies and Innovation—the CRP fosters global scientific collaboration and exchange. This presentation highlights the objectives, structure, and current activities of the CRP, emphasizing its function as a bridge between innovation policy and practical applications for sustainable agricultural productivity. The CRP is an opt-in programme, sustained through voluntary contributions from participating countries, and administered by the Trade and Agriculture Directorate (TAD) of the OECD. It is governed by a Scientific Advisory Body (SAB), which reviews applications for scientific merit, and a Governing Body, comprising one representative per member country, which approves sponsorships based on policy relevance. Key outputs include short-term international research fellowships (6–26 weeks) and support for international scientific events. In 2025, seven events—such as the present nematology symposium in Victoria, Canada—have been selected for sponsorship, with an average support of approximately €36,000 per event. The selection process prioritizes scientific excellence, international relevance, and alignment with CRP priorities. Through these activities, the CRP enables long-term global research partnerships, enhances scientific understanding, and contributes directly to the formulation of policy responses to agricultural and environmental challenges. By connecting researchers and policy-makers, the CRP plays a vital role in promoting innovation-driven, sustainable solutions for the future of global agri-food systems.

## Biotechnology and genomics for sustainable nematode management

Siddique, Shahid

Department of Entomology and Nematology, University of California Davis, 95616, Davis, CA, USA

### Abstract

Plant-parasitic nematodes (PPNs) are among the most destructive pests in agriculture, causing significant yield losses by invading plant roots and disrupting nutrient uptake and plant immunity. In California, root-knot nematodes (*Meloidogyne* spp.) and root lesion nematodes (*Pratylenchus vulnus*) pose substantial threats to tomato and walnut production, respectively. My lab integrates genomics and biotechnology to develop sustainable, non-chemical management solutions for these pests. In this presentation, I will highlight two key areas of our recent research: nematode resistance in walnuts and tomatoes. First, I will describe our strategies for managing *P. vulnus* in walnut, as current rootstocks provide limited resistance. We have sequenced the genome of *P. vulnus*, identified candidate RNAi target genes, and are currently testing their roles in infection. This approach aims to suppress nematode infectivity and reproduction through host-induced gene silencing. Next, I will address the challenge posed by root-knot nematode populations that have overcome traditional resistance in tomatoes. Our efforts include the introgression of the heat-stable Mi-9 resistance gene from wild tomato species, comparative genomic analysis to identify critical nematode effectors, and the use of CRISPR-Cas9 technology to edit host susceptibility genes, enabling durable, broad-spectrum nematode resistance.

## Non-fumigated nematicides for the management of Root-Knot nematodes in vegetable crops in California

Sidhu, Jaspreet, J. Dubose, and J. Fernberg

University of California Agricultural and Natural Resources, Bakersfield, California, 93313

### Abstract

Root knot nematodes (RKN), *Meloidogyne* spp. are the most important plant parasitic nematodes affecting the vegetable crop production in California. The root-knot nematodes can cause substantial damage by stubbing, forking, and galling the roots thereby reducing marketable yields. The galled feeder roots are unable to sustain the water and nutrient needs of the plants leading to yield reduction. Currently, there are no resistant cultivars available for the California carrot and melon industry and management has mainly relied on the use of pre-plant soil fumigants such as Telone II (a.i. 1,3-dichloropropene) and metam sodium or metam potassium. There are also incidences of resistance breakdown in tomatoes with Mi gene resistance. In addition, the new fumigant regulations by the Department of Pesticide Regulations (DPR) have been put in place and due to these regulations, substantial parts of the field or the entire field may not be treated by fumigation because of buffer zone requirements. New nematicides with novel modes of actions have emerged from major agricultural chemical companies in the last few years that have shown excellent performance in managing RKN. Fluazaindolizine (Corteva), fluensulfone (Adama), fluopyram (Bayer), and a new developmental Product have shown excellent nematicide properties in the field trials conducted in moderate to high RKN infested soils. These new chemistries have effective new modes-of action, lower mammalian toxicity, and lesser environmental impacts than previous generations of nematicides.

## Policy development to drive knowledge transfer, extension and technology adoption

Sikora, Richard A

Prof. Emeritus, Institute of Crop Science and Resource Conservation,(INRES), Plant Pathology and Nematology, University of Bonn, Germany

### Abstract

There are five terms in the title of this talk that represent what I believe are important components of the Agricultural Knowledge Chain. Innovative *technologies* for example are discoveries that only become *innovative* when they are *adopted* by growers. Adoption requires *knowledge transfer* which is driven by public and private *extension* programs. *Policy development* is extremely important, but funding is usually directed to agricultural production and is almost non-existent when it comes to extension programs. Therefore, there is an urgent need to improve the link between extension and agricultural policy development. If this link is not strengthened, research innovations will not move down the extension knowledge transfer system. In many cases, extension plant pathologists have been given responsibility for integrated nematode management. There are very few nematologists in the knowledge transfer system. Extension has also been weakened with the expansion of split-appointments over full time staffing. The former often place stress on basic research over direct farmer support. Furthermore, political support for extension on a global scale has either: 1) decreased 2) is only marginally available or 3) is non-existent. The OECD, in their *Agricultural policy monitoring and evaluation report 2024* showed that general services and support for agricultural research and innovation has declined significantly in 54 countries since 2000. These reductions impact the extension knowledge chain and ultimately sustainable productivity growth. On a global scale, funding cuts by the USA, the EU and many other donor countries for international cooperative programs, will significantly affect applied nematology research and extension in regions where it is urgently needed. The future of extension depends on whether we can effectively influence government policy on both national and international scales. Extremely important is maintaining support for initiatives targeting extension in countries where smallholder farms predominate, e.g. the 250, 126, 35, and 15 million smallholders in China, India, Africa, and South America, respectively. The question we need answer is what can be done to improve and promote extension nematology? Vision and anticipation will be important in determining the structure of agricultural extension in the future. We need to consider building consortia such as a: 1) National Center of Excellence in Extension, 2) Global Extension Hub, 3) Internet Knowledge Platform, 4) Partnerships with farm and industrial lobbies, and 5) Multidisciplinary Extension Platforms together with plant pathology and entomology. We need to reverse the downward trend in support for applied research and extension by activating government lobbying through our nematology societies: IFNS, SON, ESN, ONTA, BNS, and NSI. Finally, I believe it would be advantageous to fund a Lobby/Influencer company to promote knowledge transfer systems in the media, as well as, to identify key political decision makers that could be convinced to support to extension at government policy levels.

## Efficacy evaluation of organic bloom and organic bloom HP in the control of nematodes in soybean

Silva, Monique Thiara Rodriguese^1^, C. B. L. dos Reis^2^, A. Miamoto^1^, A. Calandrelli^1^, E. T. Sonda^1^, S. M. Santana-Gomes^1^ and C. R. Dias-Arieira^1^

^1^State University of Maringá, Dept. of Agronomy, Maringá, PR, Brazil

^2^Ingal Agrotecnology, Santa Maria, RS, Brazil

### Abstract

Nematodes such as *Meloidogyne javanica* and *Pratylenchus brachyurus* are among the main phytopathogens affecting soybean crops in Brazil, causing substantial economic losses due to their wide host range and rapid reproductive cycles. Traditional control methods face limitations, particularly in mixed infestations, which has driven research toward sustainable alternatives. Biofertilizers have emerged as promising tools, not only for their potential to modulate the rhizosphere and promote plant development but also for their capacity to induce plant resistance mechanisms against nematode invasion. This study aimed to evaluate the effectiveness of two such products, Organic Bloom and its enhanced formulation, Organic Bloom HP, in controlling *M. javanica* and *P. brachyurus* under controlled greenhouse and laboratory conditions. The experiments followed a completely randomized design with four treatments: untreated control, in-furrow application, seed treatment (ST), and combined ST plus in-furrow application. The assessments focused on nematode reproduction, plant enzymatic defense responses, juvenile nematode penetration and development, ovicidal and nematicidal activity, and overall plant growth and nodulation. Evaluations were conducted at 60 and 70 days after inoculation, and the data were statistically analyzed using the Scott-Knott test at a 5% significance level. In the 2022–2023 trial, Organic Bloom significantly reduced the number of *M. javanica* per gram of root by up to 64.22%, and *P. brachyurus* reproduction by up to 62.70%. Additionally, it triggered a marked increase in the activity of key defense-related enzymes such as phenylalanine ammonia-lyase (PAL), peroxidase (POX), polyphenol oxidase (PPO), and catalase (CAT), especially within six days of inoculation, supporting the hypothesis of induced resistance. While no significant differences were observed in shoot development, there was a clear trend toward increased root biomass in treated plants. In the subsequent 2023–2024 trial, Organic Bloom HP demonstrated superior control, reducing *M. javanica* and *P. brachyurus* by up to 73% and 76%, respectively. It also significantly reduced the penetration of *M. javanica* juveniles in the early stages post-inoculation and completely inhibited egg hatching *in vitro*. Furthermore, the product caused 100% mortality of both nematode species in laboratory assays. Importantly, none of the treatments negatively affected nodulation; on the contrary, certain application modes enhanced both the number and biomass of nodules. These findings suggest that both Organic Bloom and Organic Bloom HP were effective in reducing nematode infestation in soybean. Organic Bloom primarily acted through the induction of plant defense mechanisms, whereas Organic Bloom HP also exhibited direct nematicidal and ovicidal effects. These products did not impair plant development and even stimulated root growth and nodulation, highlighting their potential as sustainable components in integrated nematode management strategies.

## Temperature impacts on the life cycle of two Root-Knot nematode species

Silva, Monique Thiara Rodriguese^1^, C. R. Dias-Arieira^1^, I. M. C. Pereira^2^, A. F. da Silva Lima^2^, C. Freitas^2^, D. Daloso^2^, R. O. Rocha^3^

^1^State University of Maringá, Dept. of Agronomy, Maringá, PR, Brazil

^2^Federal University of Ceará, Dept of Biochemistry and Molecular Biology, Fortaleza, CE, Brazil

^3^The Connecticut Agricultural Experiment Station, Dept. of Plant Pathology and Ecology, New Haven, CT, USA

### Abstract

Nematodes significantly impact global agriculture, causing annual losses of up to $100 billion. Root-knot nematodes (RKN, *Meloidogyne* sp.) are particularly destructive. In the U.S., RKNs are widespread, and temperature changes can influence the distribution of different species. The northern root-knot nematode (*M. hapla*, NRKN) is prevalent north of 39°N latitude, while the southern root-knot nematode (*M. incognita*, SRKN) is more common south of this line. However, warmer winter temperatures—evidenced by a shift to new hardiness zones in nearly 50% of the country (USDA Plant Hardiness Zone Map, 2023)—are allowing tropical RKN species to expand their range into higher latitudes. This study aimed to investigate how NRKN and SRKN develop in response to environmental temperatures within and outside each nematode’s optimal infection range. In Experiment 1, we simulated intercropping field conditions to assess the effect of a freezing regime (4°C acclimation for 24 hours followed by −1°C incubation for 12 hours) on the egg hatching of NRKN and SRKN. The results observed indicated that SRKN had a higher hatching percentage (48.35%) than NRKN (25.43%) under the freezing treatment. In Experiment 2, the hatching of both nematodes at 23°C and 30°C was evaluated in the presence and absence of root exudates previously collected from tomatoes kept at the test temperatures. At 23°C, root exudates stimulated egg hatching, while at 30°C, hatching rates remained consistent regardless of the presence of exudates. Experiment 3 evaluated penetration, development, and reproduction of nematodes, as well as photosynthesis-related variables, in susceptible tomato plants maintained at 23°C and 30°C. Both nematode species showed slower penetration and development at 23°C; however, SRKN exhibited greater penetration and reproduction rates than NRKN at both testing conditions. Temperature did not significantly affect nematode population densities in tomatoes, although NRKN was found to be less aggressive than SRKN. NRKN reproduction rates varied from 1,727 ± 891 eggs per gram of root at 23°C to 1,177 ± 417 eggs at 30°C. In comparison, SRKN reproduction rates were 3,426 ± 1,050 eggs at 23°C and 2,996 ± 1,227 eggs at 30°C. Photosynthesis-related variables, such as SPAD, effective quantum yield of photosystem II, and linear electron flow, showed reduced activity at 30°C, indicating increased stress in plants at this temperature compared to 23°C, with SRKN inducing a more pronounced stress effect. These findings suggest that both nematode species are adapting to temperatures outside their optimal ranges. As global temperatures rise, tropical RKN species are expected to spread northward more rapidly, leading to increased crop damage and more nematode generations per growing season. Therefore, additional strategies will be necessary for effective control.

## Entomopathogenic nematodes for control of plant diseases

Slusher, Eddie Kyle^1^ and D. Shapiro-Ilan^2^

^1^Texas A&M AgriLife Research and Extension Center, Stephenville, TX

^2^USDA-ARS Southeastern Fruit and Tree Nut Research Station, Byron, GA

### Abstract

Plant diseases are a major threat to global food security through the reduction of crop yield and quality. It is estimated that global annual losses due to plant diseases exceed 30% a year. Traditionally, pathogens have been controlled using chemical management often in the form of synthetic pesticides. While these methods have been effective and necessary, overuse can lead to negative effects on biodiversity and human health while also increasing the risk of pathogen resistance. Thus, there is a need to develop alternative methods of managing plant pathogens that are more environmentally benign and cost effective. Entomopathogenic nematodes have a symbiotic relationship with *Xenorhabdus* and *Photorhabdus* bacteria. These bacteria produce secondary metabolites that have antibiotic, fungicidal, and nematocidal properties. Studies in both the lab and field have shown that these metabolites are toxic to a wide variety of plant pathogens including fungal pathogens such as *Phytophora cactorum*, *Glomerella cingulata* (anthracnose), *Monilinia fructicola* (brown rot), and plant parasitic nematodes including several species of root-knot nematode. While most of these studies have been done in the lab, field trials with peach scab yielded positive results providing evidence for future field application. In addition, studies have shown that these metabolites can enhance current commercial fungicides. Some metabolites are also very stable both at room temperature and cold storage, with some being stable after as long as 9 months. Future research should focus on further field efficacy trials and making commercial compounds that are competitive from both a management and cost standpoint with current commercial products.

## Characterizing the relationship between Soybean Cyst Nematode, soil health, and other edaphic factors in Ohio soybean fields

Small, Ambria^1^, S. Mondal^1,2^ and H. D. Lopez-Nicora^1^

^1^Department of Plant Pathology, Ohio State University, Columbus, Ohio 43210, U.S.A.

^2^Department of Plant Pathology, North Dakota State University, Fargo, North Dakota 58105, U.S.A.

### Abstract

Soybean cyst nematode (SCN), a major threat to soybean crops in North America, causes significant yield loss. While SCN relationships with soil texture have been well documented, the relationship between SCN population dynamics and indicators of soil health remains less understood. This study investigated the influence of soil health parameters and soil texture on SCN reproductive factor (RF) in Ohio with the aim to contribute data for developing integrated soil management strategies for sustainable soybean production and SCN management. In 2023 and 2024, 189 agronomic fields in Ohio were sampled at planting and post-harvest, with most following a corn-soybean rotation and some incorporating wheat or forage crops. The analysis focused on fields that grew soybeans during the study and had existing SCN populations. Relationships between soil texture and SCN reproductive factor (RF) were evaluated through Kruskal-Wallis and Spearman’s correlation analysis. A multiple linear regression model was used to explore the relative importance of soil texture components compared to the presence of a host crop influencing SCN population dynamics. Soil health indicator relationship with SCN RF was investigated by stepwise logistic regression. Soil texture showed no significant effect on SCN RF. However, the presence of soybean significantly increased SCN RF (*P* = 0.013), indicating that SCN reproduction was higher in fields planted with soybean, regardless of soil texture. Organic matter (OM), macroaggregates, and permanganate-oxidizable carbon (POxC), all indicators of soil biological activity, were associated with SCN RF, suggesting that SCN reproduction may increase under conditions of improved soil health. This study does not directly assess the impact of soil health indicators on SCN population dynamics. Improved soil health may enhance soil moisture retention and support biodiversity, creating a more favorable environment for soybean growth. A healthier host could, in turn, serve as a more suitable food source for SCN. These findings highlight the need to monitor soil health indicators and SCN abundance in soybean production fields to mitigate SCN pressure and optimize soybean production. While enhancements in soil health may support plant vigor, they should not replace effective integrated SCN management strategies, such as crop rotation and the use of soybean cultivars with genetic resistance to SCN.

## *Hyalorbilia* SPP. Isolated from parasitized sugarbeet cyst nematodes also suppress populations of soybean cyst nematodes

Smith Becker, Jennifer^1^, J. Borneman^2^, J. Yang^1^ and J. O. Becker^1^

^1^Dept. of Nematology

^2^Dept. of Microbiology and Plant Pathology, University California, Riverside, CA 92521

### Abstract

Three nematophageous *Hyalorbilia oviparasitica* clade strains, DoUCR50, HsImV27, and ARF, were tested for their ability to suppress the reproduction of soybean cyst nematodes (SCN, *Heterodera glycines*) in greenhouse tests. DoUCR50 is a hyperparasite of the sugarbeet cyst nematode *Heterodera schachtii*. It was discovered as the primary biological entity responsible for a long-term (>35 years) population suppression of *H. schachtii* in field 9E at the University of California Riverside’s Agricultural Operations. HsImV27 was isolated from *H. schachtii* females reared in field soil from a commercial sugar beet field in the Imperial Valley, CA. In a prior study, HsImV27 suppressed *H. schachtii* populations by more than 80% in soil-based greenhouse assays. ARF was isolated from *H. glycines* cysts collected from soybean fields in Arkansas. The strain was previously shown to suppress *H. glycines.* The three fungi were cultured in powdered peat for use as soil inoculum in nematode suppression tests. Autoclaved soil was amended with 500 CFU/cm^3^ of each fungal strain. Controls were amended with autoclaved peat inoculum. Plastic cones (200 cm^3^) containing treatment soils were seeded with soybean (*Glycine max* cv. Williams 82) and inoculated with 250 J2 of *H. glycines* 3 weeks later. After about 2 months (1260 degree days), cysts and females from each cone were collected by flotation sieving and enumerated under a dissecting scope. DoUCR50, HsImV27, and ARF suppressed nematode (cyst + female) numbers of *H. glycines* by 73%, 87%, and 0%, respectively. The failure of ARF to suppress *H. glycines* in these experiments was likely due to its long-term culture on artificial media. Egg parasitism by DoUCR50 and HsImV27 was tested *in vitro* by incubating eggs of *H. schachtii* and *H. glycines* on water agar cultures of the fungi. Forty undifferentiated eggs from females of each nematode species were placed either singly or in groups of 2 or 4 on the fungi, and the percentage of eggs that were parasitized was determined after 6 days. The susceptibility of eggs to DoUCR50 depended on the number of eggs grouped together. Individually spaced eggs of *H. schachtii* or *H. glycines* were parasitized at a rate of 15% or 13%, respectively. Eggs placed in pairs on DoUCR50 were parasitized at 38% and 43%, and eggs placed in groups of 4 were parasitized at 70% and 63%, respectively. HsImV27 parasitized fewer than 10% of eggs of either nematode, regardless of egg grouping size. HsImV27’s ability to reduce nematode populations is likely due to female parasitism. The ability of *Hyalorbilia* spp. isolated from *H. schachtii* to suppress *H. glycines* with similar efficacies offers promising opportunities to expand the use of these fungi as potential biocontrol agents.

## Impacts of Root-Knot nematodes (*Meloidogyne chitwoodi* and *M. hapla*) on potato yield and quality in the Pacific Northwest

Studebaker, Gabrielle^1,2^, I. Zasada^2^, D. I. Thompson^3^, and V. Sathuvalli^3^

^1^Oregon State University, Dept. of Botany and Plant Pathology, Corvallis, OR 97331

^2^USDA-ARS, HCRPMRU, Corvallis, OR 97331

^3^Oregon State University, Hermiston Agricultural Research and Extension Center, Hermiston, OR 97838

### Abstract

The root-knot nematodes, *Meloidogyne chitwoodi* and *M. hapla*, are important pests in the potato production regions of the Pacific Northwest. *M. chitwoodi*, is a quarantine pest and is known to cause galling and blemishes on the tuber flesh, resulting in crop rejection. Given the low tolerance to these nematodes in the potato industry, our work aims to understand the relationship between initial nematode density in soil and tuber quality and yield. A fumigated field was planted with three varieties of potato (Russet Burbank, Ranger Russet, and Clearwater Russet) and inoculated with initial densities of either *M. hapla* (0, 50, and 200 eggs/250 cc of soil) or *M. chitwoodi* (0, 25, 50, and 200 eggs/250 cc of soil). At harvest, tubers were sorted and assessed for total weight, tuber number, and external and internal quality. Graded tubers were then stored for three months at 4 °C before a subset (n = 10) from each treatment group was peeled and graded for damage caused by *M. chitwoodi* and *M. hapla*. Our results, based on two years of data, indicate that *M. chitwoodi* at the 25 eggs/250 cc of soil inoculation density caused more than 10 nematode infections across all varieties. *M. chitwoodi* reduced yield of ‘Clearwater Russet’ by approximately 40% at the highest initial density compared to the non-inoculated control in the first year (*P* = 0.025). Increasing severity of galling was observed with increasing initial inoculation densities for *M. chitwoodi*. Further, it was observed that ‘Ranger Russet’ tubers were infected by *M. hapla* more readily compared to other varieties. ‘Ranger Russet’ tubers had greater than six nematode infections at the highest initial density compared to ‘Clearwater Russet’ and ‘Russet Burbank’ that had less than five at the highest initial density. Overall, *M. hapla* had no impact on total yield or tuber number. In the second year, there was a non significant observed reduction in total yield of ‘Clearwater Russet’ and ‘Russet Burbank’ compared to the non-inoculated control for *M. hapla*, this difference was not observed between the low and high inoculation densities. Results from this research will allow for a better understanding of the relationship between root-knot nematode densities in soil and their impacts on tuber yield and quality in the region.

## Phylogeny and phylogeography of the cyst nematode species of the *Goettingiana* group from the genus *Heterodera*

Subbotin, Sergei A.^1^, J. E. Palomares-Rius^2^, M. Córdoba-Sánchez^2^, T. V. Roubtsova^3^, R. M. Bostock^3^, S. Fournet^4^, E. Grenier^4^, P. Veronico^5^, Z. Tanha Maafi^6^ and P. Castillo^2^

^1^Plant Pest Diagnostic Center, California Department of Food and Agriculture, Sacramento, CA, USA

^2^Institute for Sustainable Agriculture (IAS), Spanish National Research Council (CSIC), Department of Crop Protection, Córdoba, Spain

^3^Department of Plant Pathology University of California, Davis, CA 95616, USA

^4^IGEPP, INRAE, Institut Agro Rennes-Angers, University of Rennes, Le Rheu, 35653, France

^5^Institute for Sustainable Plant Protection, National Research Council of Italy, Bari, Italy

^6^Iranian Research Institute of Plant Protection, Agricultural Research Education and Extension Organization, Tehran, Iran

### Abstract

Cyst nematodes of the genus *Heterodera* are obligatory sedentary endoparasites of great economic importance throughout the world. The *Goettingiana* group of this genus consisted of 17 species parasitizing dicotyledons and are characterized by lemon-shaped cysts having an ambifenestrate cone, long vulval slit, weak underbridge and presence or absence of bullae. In this study, we provided comprehensive phylogenetic analyses of 164 (126 new) *COI* and 108 (46 new) ITS rRNA gene sequences from 45 nematode populations of eight nominal species and several unidentified species of the *Goettingiana* group, including *H. carotae, H. circeae, H. cruciferae, H. goettingiana, H. microulae, H. persica, H. scutellariae* and *H. urticae* using Bayesian inference, maximum likelihood, and statistical parsimony. The carrot cyst nematode *H. carotae* in California and on wild *Daucus* sp. sampled in France is reported for the first time. The nettle cyst nematode in Spain and France is also reported for the first time. Our study showed that the ITS rRNA gene sequence can be used for discrimination of some species from the *Goettingiana* group, however, it did not allow differentiating the *H. cruciferae* species complex species consisting of *H. carotae, H. cruciferae, H. urticae* from each other. The *COI* gene sequences clearly distinguished all studied species of the *Goettingiana* group from each other and can be recommended as a DNA barcoding marker for this group. *COI* intraspecific sequence diversity of *H. goettingiana* was highest (11.4%) among the *Goettingiana* group. It has been hypothesised that the majority of the *Goettingiana* group species originated and diversified in regions located in Western and Eastern Asia and Central and Western Europe during the Pleistocene and then dispersed from these regions across the world. Further cyst nematode surveys should be conducted in different world regions to obtain new datasets, which allow identifying centers of diversification of this species group.

## Soil nematode communities impacted by human decomposition: where ecology meets forensic science

Taylor, L. Stacy^1^, E. C. Bernard^2^ and J. M. DeBruyn^1^

^1^Department of Biosystems Engineering and Soil Science

^2^Department of Entomology and Plant Pathology, University of Tennessee, Knoxville, TN 37996

### Abstract

Soils subjected to vertebrate (human and animal) decomposition undergo considerable biogeochemical and microbial transformation across timescales ranging from months to years. Vertebrate tissue is a high-quality resource containing elevated quantities of nitrogen (N) as well as a unique microbiome. The decomposition of these tissues exerts a profound effect on soil physicochemistry and microbial communities producing localized hotspots with altered pH, elevated temperature, temporary hypoxia, elevated N (including ammonium and nitrate) and a coalesced microbial community. The dynamic changes in the soil are expected to impact higher orders of the soil food web. To examine these impacts we assessed population dynamics of free-living soil nematodes across two year-long seasonal human decomposition experiments (beginning in spring and winter) at the University of Tennessee Anthropology Research Facility, an outdoor human decomposition laboratory. Six deceased human subjects were enrolled, divided between the two trials. Over a year of decomposition, soils were sampled underneath both subjects at two depths 0–1 cm (interface) and 1–16 cm (core). Nematodes were extracted by sugar flotation centrifugation and identified to family level by microscopy. Overall, nematode abundance, richness, alpha-, beta-, and functional diversity responded strongly to decomposition, and the magnitude of changes differed seasonally. In the spring study, the period of greatest mass loss (active and early advanced decay) was characterized by a sharp decrease in nematode abundance and all diversity metrics. Abundance decreases (< 30 taxa 100 gdw^−1^ soil) coincided with soil acidification, elevated soil temperatures, and reduced oxygen in both soil depths. In the winter study, soil physicochemical changes were more subdued: soil heating from insect thermogenesis was not present, and there was a protracted seepage of decomposition fluids in contrast with the pulsed fluid release observed in the spring. As a result, nematode abundances were an order of magnitude greater compared to spring samples, peaking at 116,248 ± 76,810 and 29,708 ± 23,622 taxa 100 gdw^−1^ soil, respectively. Community composition in impacted soils was dominated by bacterial enrichment opportunists (Rhabditidae, Diploscapteridae, and Diplogastridae) with Enrichment and Structure Index values of 100 (EI) and zero (SI), respectively. Later timepoints showed periodic increases of *Aphelenchoides* and *Acrobeloides*, however successional patterns did not emerge. Our findings demonstrate that seasonality plays a role in nematode community dynamics in decomposition hotspots and offers insights into the specific influence of soil temperature. This work has the potential to contribute to two unique applications: evaluating environmental impacts of animal mortality events, and modelling combined changes in soil parameters to form time-since death estimates in forensic taphonomy.

## Genome reannotation and effector candidate identification in *Meloidogyne chitwoodi* through gland-specific transcriptome analysis

Teixeira, Marcella^1^, I. Ko^1^, S. Bali^1,#^, P. Vieira^2^, T. Maier^3^, T. Baum^3^ and C. Gleason^1^

^1^Department of Plant Pathology, Washington State University, Pullman, WA 99164

^2^Mycology and Nematology Genetic Diversity and Biology Laboratory, USDA Agricultural Research Service, Beltsville, MD 20705

^3^Department of Plant Pathology, Entomology and Microbiology, Iowa State University, Ames, IA, 50011

^#^Current Address: Simplot Plant Sciences, J. R. Simplot company, 5369 W Irving St., Boise, ID 83706

### Abstract

The Columbia root-knot nematode (CRKN), *Meloidogyne chitwoodi*, infects potato roots and tubers, leading to substantial reductions in tuber yield, product quality, and export potential. This nematode secretes effector proteins that suppress plant immune responses and facilitate successful parasitism. Investigating these effectors is essential for developing alternative strategies to manage CRKN. Because effectors are typically produced in the esophageal glands, we conducted the first gland-specific transcriptome analysis for CRKN to identify novel effectors. This analysis led to the discovery of previously uncharacterized, gland-specific proteins. In situ hybridization confirmed gland localization for a selected subset of transcripts. Expression profiling across the CRKN life cycle revealed elevated expression of these transcripts in pre-parasitic second-stage juveniles (J2s), suggesting a role in the early stages of host infection. Furthermore, transgenic plants ectopically expressing two of the putative effectors exhibited altered susceptibility to CRKN, supporting their involvement in parasitism. Our findings demonstrate that integrating gland-specific transcriptomics with high-quality genome annotations and stringent selection criteria significantly improves effector identification. Preliminary evidence also supports the functional role of these novel effectors in CRKN parasitism. Future research will focus on identifying their molecular targets in potato plants.

## The compatibility of Salibro™ with non-target nematode species of different feeding habits

Thapa, Sita^1^ and T. Thoden^2^

^1^Corteva Agriscience, Indiana, IN 46268

^2^Corteva Agriscience, München, Germany, 81677

### Abstract

Salibro™ (Reklemel™ active, fluazaindolizine) is a novel, nonfumigant, chemical nematicide developed by Corteva Agriscience. It has excellent activity on root-knot nematodes and in previous studies has shown to be selective to various non-target nematode species. Approximately 30 thousand nematodes have been described, of which most have an important role in soil ecosystems and consequently soil health as well as sustainability. The main objective of this project was to extend our understanding of the compatibility of Salibro with additional species and feeding behaviors of non-target nematode species in comparison to other commercially available nematicides. Three species of bacterial feeders (*Acrobeloides butschlii*, *Mesorhabditis spiculigera* and *Panagrellus redivivus*); one fungal feeder (*Aphelenchus avenae*), one predator (*Mononchus aquaticus*), one omnivore *Dorylaimus* sp. and two entomopathogenic nematodes (*Heterohabditis bacteriophora* and *Steinernema feltiae*) were studied. During our talk we will present novel data from various testing methods (*in vitro* vitality studies, horizontal movement studies, etc.) that will confirm the overall selectivity of Salibro to non-target beneficial nematodes.

## Impact of government policies on global trends in nematicide and bio-control development – industry perspective

Thoden, Tim, A. Alix, T. Wilson, J. A. Wiles, C. Hazel and K. Bosc

Corteva Agriscience, Germany

### Abstract

Government policies such as regulator y and environmental legislations, trade policies or public health programs have manifold impacts on the discovery, development as well as the life cycle of crop protection (CP) products – including chemical or biological nematicides. We will share our industry perspective on certain aspects of current regulatory systems around the world and discuss how those might be streamlined or leveraged to support the development of innovative, and effective nematode management solutions that can address both farmers’ and society’s expectations of safety and sustainability. We will: (1) include a case study on the socio-economic and ecosystem benefits of using nematicides; (2) show the benefits of EPA’s reduced risk pesticide program (with a link to Reklemel™ active); and (3) discuss challenges that we foresee with respect to the development of integrated nematode management programs that try to combine the benefits of both synthetic and biological nematicides.

## How can we make the agroecosystems more resilient towards nematodes?

Topalović, Olivera^1^, M. Vestergård^2^, H. Heuer^3^, S. Geisen^4^ and F. Ekelund^1^

^1^Department of Biology, University of Copenhagen, Copenhagen, Denmark

^2^Department of Agroecology, Section for Plant Pathology and Microbiology, Aarhus University, Slagelse, Denmark

^3^Institute of Epidemiology and Pathogen Diagnostics, Julius Kuehn Institute, Braunschweig, Germany

^4^Laboratory of Nematology, Wageningen University and Research, Wageningen, The Netherlands

### Abstract

The biological management of plant-parasitic nematodes relies on beneficial microorganisms in soil that can directly or indirectly suppress these notorious pests. Nonetheless, the biological management of nematodes is challenging partly because complex interactions between nematodes, plants and associated microbiomes are overlooked in current management strategies. Here, I summarize our research in the field of nematode biocontrol, where we study how nematode-microbe associations in nematodesuppressive and nematode-conducive soils impact the nematode and plant performance. Firstly, we showed that microbiomes specifically attach to the nematode surface. Factors such as the soil microbiomes, nematode species and interactions among the surrounding microbiota influence microbial attachment to nematodes, nematode activity and invasion into the roots. Moreover, we found that the surface of active nematodes harbors microbial taxa with a potential to protect nematodes against the antagonists, hence interfering with nematode biocontrol. Secondly, we demonstrated that nematode-associated microbes mediate the plant responses to nematode invasion. Microbes from nematode-suppressive soils induced systemic resistance in plants including transcriptional responses and ROS production, limiting the nematode performance on plants. Thirdly, we showed that nematode parasitism is followed by a restructuring of the microbiomes in the plant rhizosphere and endosphere and that these changes depend on the plant host as well as the introduced microbial agent. Finally, I will discuss the challenges in the current biological strategies to control plant-parasitic nematodes and propose future perspectives that rely on a holistic understanding of nematodes as holobionts and their interactions with plants and the surrounding soil biota.

## Utilizing essential oils for organic nematode control in a new soil disinfectant, RevoCURB™

Trainer, Emma, B. Hatfield and E. Fuerst

Kemin Crop Technologies, Des Moines, IA 50317

### Abstract

Soil-borne pests damage 60–75% of crop yields annually through disease, weed competition, and plant-parasitic nematodes. While conventional crop growers can combat these pressures with soil fumigants applied prior to planting, the chemistries used for fumigation are often hazardous, have strict application requirements, and exclude use on organic acreages. With the market need for organic pre-plant soil disinfectants in mind, Kemin Crop Technologies set out to create a safe-to-use product leveraging the natural activity of thyme, clove, cinnamon, and garlic essential oils. These four essential oils are rich in bioactive compounds like thymol, eugenol, cinnamaldehyde, and allyl sulfides, respectively, which have demonstrated efficacy against plant pests through cellular membrane disruption, repellency and olfactory effects, and nervous system interactions. RevoCURB™, a new OMRI-certified pre-plant soil treatment, was formulated to target soil-borne plant pathogens, weed seeds, and parasitic nematodes by leveraging the combined activity and modes of action of these four essential oils. Multiple studies were conducted against plant-parasitic nematode species (*Meloidogyne* spp.), ranging in scale from bioassays and greenhouse pot trials to in-field evaluations. Bioassays completed at the University of Florida demonstrated complete control of *Meloidogyne javanica* juveniles, inducing dissolution of J2 juveniles in under 24 hours. In a follow-up greenhouse trial challenging cucumbers with *Meloidogyne enterolobii*, RevoCURB showed a clear dose response in predictive fit models with 90% egg reduction at a concentration of 0.53% and a 90% reproductive factor reduction at a concentration of 0.70%. RevoCURB provided control of all nematode factors at a rate of 0.5% compared to the inoculated negative control and had improved cucumber vigor metrics often comparable to fluopyram treatments. Field assays performed across the United States on a variety of crops, including cucumber, tomato, and onion, demonstrated consistent control of both root-knot nematodes and pin nematodes resulting in improved crop health and/or yields. Furthermore, soil retention assays completed on both organic matter-rich clay loam soil and loamy sand soil demonstrated 30–100% residency of active molecules past 14 days, indicating that the essential oils in RevoCURB can provide extended control of soil-associated pests in a safe-to-use and organic formulation.

## Providing resistant soybean variety information and research results to help farmers actively manage Soybean Cyst Nematode

Tylka, Gregory, C. Marett, G. Gebhart, M. Mullaney and J. Rasmussen

Iowa State University, Dept. of Plant Pathology, Entomology, and Microbiology, Ames, IA 50011

### Abstract

The soybean cyst nematode (SCN), *Heterodera glycines*, is the most damaging pathogen of soybean, *Glycine max*, in the United States. A primary tool for managing SCN is resistant soybean varieties, which limit SCN parasitism and therefore reduce yield loss. We annually compile a list of SCN-resistant varieties for farmers, providing agronomically important characteristics for each variety including the soybean breeding line used to confer SCN resistance. Availability of resistant varieties increased from 29 in 1991 to over 900 in 2025. The PI 88788 breeding line has been the primary or sole source of resistance in the varieties every year. Varieties with resistance from the Peking breeding line started to become increasingly available in 2023. The amount of SCN control each variety offers can vary due to the complex nature of genes conferring resistance. Also, in the United States, resistant varieties are not required to provide a specific level of SCN control. Consequently, we have evaluated resistant soybean varieties in experiments in SCN-infested fields throughout Iowa since 1994, measuring yields and beginning- and end-of-season SCN population densities in replicated plots. Data are statistically analyzed by variety and location, and results are compiled into annual reports made available in print and online. Also, in every field experiment the reproduction, or virulence, of the SCN population on resistant soybean breeding lines is measured by determining female indices in an HG type test. Results of 225 HG type tests from 2000 to 2024 revealed a steady increase in virulence on PI 88788, but not on Peking. Scientists in other states also have reported SCN populations with increased reproduction on PI 88788. As a result, the SCN Coalition was launched in 2018 to educate farmers about this increasing problem. The SCN Coalition involves university scientists from soybean-producing states in the United States and in Ontario, Canada plus numerous personnel from private companies, all organized, coordinated, and led by an agricultural marketing agency using a multifaceted approach to educate farmers and agribusiness personnel about decreased effectiveness of PI 88788 SCN resistance and how to mitigate the associated yield loss. In 2023, the SCN Coalition created an online calculator, the SCN Profit Checker, using data from >35,000 variety evaluation research plots mentioned above to estimate soybean yield reduction in a field due to ineffective PI 88788 resistance. For any SCN-infested field, users can provide values for soil pH and sand content, SCN population density, and SCN female index on PI 88788 from an HG type test and the calculator will estimate the likely yield reduction and associated economic value of the yield loss. Overall, the activities above have increased awareness among farmers of the complexities of managing SCN with resistance and resulted in more farmers actively working to reduce SCN yield loss.

## Global soil nematode abundance under current and future climate scenarios

Van den Hoogen, Johan^1^, Stefan Geisen^2^ and Thomas Crowther^1^

^1^Department of Environmental Systems Science, ETH Zürich, Switzerland

^2^Laboratory of Nematology, Wageningen University, The Netherlands

### Abstract

Soil nematodes are a crucial part of the terrestrial biosphere and are the most abundant animals on Earth, filling all trophic levels in the soil food web. Despite their importance for ecosystem functioning, few quantitative, spatially explicit models of the active belowground community exist. I will present our 2019 study on the patterns of the global abundance and functional group composition of soil nematodes. The resulting maps show that 4.4 × 10^20^ nematodes (with a total biomass of approximately 0.3 gigatonnes) inhabit surface soils across the world, with higher abundances in sub-Arctic regions (38% of total) than in temperate (24%) or tropical (21%) regions. In addition, I will present preliminary results from ongoing projects that focus on potential future trajectories of soil nematode communities under different climate scenarios, highlighting regions that may be at risk.

## Developing database-driven Plant-Parasitic Nematode risk assessments for Hawai’i with machine learning

Wei, Xing^1^ and J. Marquez^2^

^1^Department of Plant and Environmental Protection Sciences, University of Hawaii at Manoa, Honolulu, HI 96822

^2^Hawaii Department of Agriculture, Honolulu, HI, 96814

### Abstract

Hawaii is centrally located between Asia and the Americans and can be a gateway for invasive species introduction, including plant parasitic nematodes (PPNs) between the East and West. Proactive quarantine measures for important PPNs can reduce significant costs associated with plant damage and management. However, the biosecurity threats of new PPN introductions in Hawaii are poorly understood for many newly discovered and described species. This study aims to develop a risk assessment tool to evaluate a large list of PPNs, helping to identify potential threats that may otherwise be overlooked. An impact metric system (IMS) was developed to understand the threat level to Hawaii’s agriculture and ecosystem from PPNs either not known to occur or currently limited in distribution within the state. The IMS was built upon scoring 14 different metrics based on data from plant host and PPN databases to assess their ability to establish and impact plants in Hawaii. This includes county-level distribution data for inter-island quarantine purposes. Once each host-PPN relationship was scored, each PPN was ranked with machine learning from a training set of PPNs that are currently established with known impact to Hawaii. A pilot run was conducted by collecting a list of potentially high-risk PPNs from the Hawaiian Invasive Species Council, California Department of Food and Agriculture, Cooperative Agricultural Pest Surveys, and CABI Horizon Scanning Tool. Limitations in distribution data and the lack of severity of yield and symptoms caused by the nematode can contribute to poorly ranked PPNs, therefore, human investigation on the impact is still required to validate IMS ranks. Currently, no computer-assisted pest risk analysis is available for Hawaii’s biosecurity. Therefore, this tool will enhance the state’s ability to conduct horizon scans to proactively prevent the introduction of new PPNs.

## Thermal tolerance of peach tree rootstocks

Westerdahl, Becky and C. Anderson

University of California, Dept. of Entomology and Nematology, Davis, CA 95616

### Abstract

Pretreatment fumigation of soil is a common practice in the production of fruit tree rootstocks. Recent restrictions on the use of some fumigants and reductions in allowable rates of others increase the potential for production of contaminated planting stock. Thermal treatments of planting stock for nematode management have been used commercially in California for daffodil, Easter lily, garlic, and strawberry planting stock. Hot water treatment of infected fruit tree rootstocks is a potential option in the event of a fumigation failure. To establish thermal tolerances, for ‘Lovell’ and ‘Nemaguard’ rootstocks, each year for three successive years, the roots of commercially grown rootstocks were treated in a water bath for 5 lengths of time at each of 5 temperatures (43.3 °C, 46.1 °C, 48.9 °C, 51.7 °C, 54.4 °C) plus an untreated control with 5 replicates per treatment. Rootstocks treated each year were planted in a 0.4 ha field site and evaluated for survival and growth over a two-year period. For ‘Lovell’, 2-year survival of untreated rootstocks ranged from 40 to 60 percent. Eighty percent or greater survival was achieved at the following temperature/time combinations: 43.3 °C for 30, 40, and 50 minutes; 46.1 °C for 20 and 25 minutes; and 54.4 °C for 1.5 minutes. For ‘Nemaguard’, 2-year survival of untreated rootstocks ranged from 60 to 100 percent. Eighty percent or greater survival was achieved at the following time/temperature combinations: 43.3 °C for 30, 40, 50, 60, and 70 minutes; 46.1 °C for 15, 20, and 25 minutes; 48.9 °C for 5 and 10 minutes; and 51.7 °C for 1.5 and 2.5 minutes. Available information on thermal tolerance of nematodes indicates these time/temperature combinations would be sufficient to kill *Meloidogyne javanica* and *Pratylenchus vulnus* but not *M. incognita*. For ‘Lovell’, for all three years, trunk diameter of surviving trees at 2-years was numerically greater than untreated trees at 43.3 °C for 50 minutes; at 46.1 °C for 15, 20, and 30 minutes; at 48.9 °C for 5 minutes; and at 51.7 °C for 4.5 and 6.5 minutes. For ‘Nemaguard’, for all three years, trunk diameter of surviving trees at 2-years was numerically greater than untreated trees at 43.3 °C for 70 minutes; at 46.1 °C for 15 minutes; and at 54.4 °C for 1.5 minutes. Overall, at 2-years, there was a trend for treated trees to have a greater trunk diameter than untreated trees.

## Management considerations for Plant-Parasitic Nematodes in annual and perennial crops based on their distribution patterns and migratory abilities

Westphal, Andreas

University of California, Riverside, Dept. of Nematology, Kearney Agricultural Research and Extension Center, Parlier, CA 93648

### Abstract

Cropping systems greatly impact the severity and prevalence of nematode diseases in crops. Commonly, nematode management strategies for annual crops focus on the upper 30 cm of soil depth that is most impacted by tillage. Management provides at least annual intervention opportunities in these crops and includes tillage intensity, crop sequence/rotation, cover cropping, nematicide applications, and the choice of resistant or tolerant crop cultivars. But nematode movement from deeper soil layers was reported in annual crops. For example, *Heterodera schachtii* infected new plantings of sugar beet that were grown in a *H. schachtii*-free 30-cm topsoil layer, from infested layers below 30-cm depth, soon after planting. In fumigation experiments with *Rotylenchulus reniformis*, shallower soil layers were recolonized from much deeper soil depths during one growing season, while limited recolonization of deeper soil layers was observed during this time. So, in annual systems, greater soil depths can serve as reservoir for nematode infestations. Because of narrow economic margins, active management of population densities below plowing depth is challenging. In contrast, perennial systems offer the most effective window for preplant soil treatments for nematode suppression, which occurs only once during the 25–40 year cropping cycle for almond and walnut, respectively. Because almond, pistachio, and walnut are all susceptible to *Pratylenchus vulnus* rotating among these crop species when developing a new orchard has limited value. Sensitivity to *P. vulnus* increases from relatively low in pistachio to almond to the most sensitive walnut. Soils under these crops can harbor similarly high population densities throughout the upper 1.5 m of soil depth. This entire depth is targeted by preplant treatment options to reduce nematode population densities below damage thresholds. Intensive soil tillage to at least this depth renders soils treatable by chemical and biorational preplant options. Soil fumigation with 1,3-dichloropropene, often in mixes with chloropicrin, has been a standard soil treatment. With the increasing restrictions and cost limitations, alternative preplant soil treatments are being developed. Some of which may be less efficacious and require postplant remedies to combat fluctuations in remaining population densities. Because roots grow into greater soil depths, residual nematodes at these depths, which are significantly more challenging to access with conventional treatments, can quickly reach tree roots, further impeding tree growth. In nut tree crops, emphasis is placed on rootstock development to combat nematode infection and growth reduction. Although the breeding process is tedious, several elites have been identified in almonds and walnuts. Field testing and implementation of these rootstocks is a time-consuming process, but it offers the appeal of sustainable plant protection.

## Implementing velvet bean cover crop for management of reniform nematodes on sweetpotatoes in Hawaii: A case study for collaborating with farmers in pest management innovation

Wiseman, Benjamin^1^, T. Le^2^ and K.-H. Wang^1^

^1^Department of Plant and Environmental Protection Sciences, University of Hawai‘i at Mānoa, HI 96822

^2^Department of Family and Consumer Sciences, University of Hawai‘i at Mānoa, Honolulu, HI 96822

### Abstract

There is a need to incorporate farmers in dialogue and reflection in the iterative process of developing pest management tools, especially for organic pest management which often requires proactive and coordinated implementation of multiple techniques. This research uses community-based participatory research to develop velvet bean (*Mucuna pruriens*) cover cropping as an organic nematode management technique for sweetpotatoes (*Ipomea batatas*). Using five case studies, we investigated the on-farm performance of velvet bean cover crop as a technique to manage reniform nematodes (*Rotylenchulus reniformis*) and promote soil health in a sweetpotato agroecosystem while exploring the attitudes, perceptions, and values that guide adoption of organic nematode management techniques by farmers. Five farms were selected using convenience sampling from the contacts of the researchers. At each farm, velvet beans were grown in three replicated plots at a rate of 34 kg seeds/ha and compared to three plots grown according to the current practice of the farmer of either buckwheat (*Fagopyrum esculentum*) cover crop or fallow. The soil nematode community, soil microbial respiration, total carbon, and soil aggregate stability were measured at planting and termination of the cover crop and three months after planting the sweetpotatoes. Velvet bean enhanced soil health while mitigating reniform nematodes in plots with high reniform pressure. The enrichment index (EI) showed that farms that started with depleted soil food webs transitioned to enriched food webs during the velvet bean cover crop (marginally significant, *P* < 0.10). Only one farm showed an initial population of reniform nematodes greater than 500 nematodes/cm^3^ soil (870 nematodes/cm3 soil) in velvet bean plots, and the velvet bean reduced the population to 330 nematodes/cm^3^ soil at termination of the velvet bean (*P* < 0.01). Throughout the study, the attitudes, values, and priorities of farmers were explored through qualitative inquiry using semi-structured in-person inter views, photovoice, field notes, and a farm-log. The interviews were coded to identify major themes, and triangulation between qualitative methods identified attitudes, perceptions, and values of farmers related to velvet bean as a nematode management technique. Initial results indicated that farmers seek to avoid pest damage by growing crops that they have previously grown successfully with little or no pest management. Farmers stated that they were not typically able to experiment with pest management techniques due to time limitations and financial risk. The findings point to a need to minimize risk for farmers trialing new nematode management techniques. This study demonstrates participatory research as a method for nematology researchers to collaborate with farmers in nematode management innovation and builds support for velvet bean as an effective cover crop for suppressing reniform nematodes and promoting soil health.

## Molecular characterization of *Meloidogyne konaensis* from the type locality and insights into the molecular diagnosis of coffee Root-Knot Nematode species

Wong, Landon G. K.^1,2,*^, B. Sipes^1^ and R. Myers^2^

^1^University of Hawaii at Manoa, College of Tropical Agriculture and Human Resilience, Honolulu, HI 96822, USA

^2^USDA, Agricultural Research Service, DKI-PBARC, Hilo, HI 96720, USA

### Abstract

The Kona coffee root-knot nematode, *Meloidogyne konaensis* Eisenback, Bernard & Schmidt, 1993 was isolated from coffee roots in Hawaii and described based on morphology, esterase pattern (F1), and chromosome number (2n=44). Initial genetic studies generated a genomic DNA sequence from the 28S D2-D3 expansion segment and a mitochondrial DNA sequence from the cytochrome oxidase 1 (COX1) from a population collected on coffee roots from the type locality by Serracin in 1997. Increased reliance on molecular tools for identification of *Meloidogyne* spp., the limited molecular characterization of specimens from Hawaii, and the morphological and biochemical resemblance of *M. konaensis* to other coffee-parasitizing *Meloidogyne* species has led to confusion regarding its taxonomic description and placement. To clarify the taxonomic placement of *M. konaensis*, the 1997 reference culture maintained on coffee at the University of Hawaii was characterized morphologically, biochemically, cytogenetically, and genetically. Morphological features (6–12 projections on male stylet), esterase (F1), and chromosome number (2n=44) matched the original description. Key features such as the perineal pattern, 6–12 projections on the male stylet, and coffee corky root symptoms indicate the nematode is nearly identical to specimens from the original description. Subsequently, sequences from the ITS gene region, the 18s gene region, as well as mitochondrial DNA from the COX2 and NAD5 gene regions were generated. Phylogenetic analysis of the five DNA sequences from the reference culture placed the type locality population in the *Meloidogyne incognita* group (MIG). Bayesian trees clustered *M. konaensis* from the type locality with *M. paranaensis* and *M. izalcoensis*. Five populations of *M. konaensis,* including the reference culture, were evaluated using an array of SCAR primers for the identification of coffee root knot nematodes and MIG species. Males, females, and juveniles amplified par-Co9F/R for *M. paranaensis* at the expected band size of 208 bp, but did not amplify Finc/Rinc, Fjav/Rjav, and Far/Rar SCARs. Malate dehydrogenase (N1) from the *M. konaensis* reference culture was similar to numerous MIG species. The results suggest the par-Co9F/R SCAR primer is not species specific and can be used to rapidly detect *M. paranaensis* and *M. konaensis.* Further, molecular assays and phylogenetic analysis suggest *M. konaensis* and other species of *Meloidogyne* on coffee are a complex needing further investigation.

## Update of the guava Root-Knot Nematode *Meloidogyne enterolobii* in North Carolina, USA

Ye, Weimin

Nematode Assay Section, Agronomic Division, North Carolina Department of Agriculture & Consumer Services, Raleigh, NC 27607

### Abstract

*Meloidogyne enterolobii* Yang & Eisenback, 1983 was originally described from the pacara earpod tree (*Enterolobium contortisiliquum* (Vell.) Morong) in China and later was found in over 31 countries, mainly in tropical areas. It is considered to be one of the most damaging species of root-knot nematodes (RKN) in the world because of its wide host range, aggressiveness, and ability to overcome the resistance that has been developed against RKN in many crops. In the USA, it was first detected from ornamental plants in Florida in 2001, but is now confirmed in North Carolina, South Carolina, Georgia and Louisiana, probably through movement of infested plants. *Meloidogyne enterolobii* is a recently detected and emerging RKN species in North Carolina, causing severe damage to sweetpotato, soybean, and cotton. From 2006 to 2025, thousands of RKN populations were collected from North Carolina field crops, ornamental plants and turfgrasses for species identification in the Nematode Assay Laboratory in the North Carolina Department of Agriculture & Consumer Services. Root systems showing galling symptoms were dissected under the microscope and females were obtained for DNA analysis. When samples were submitted as soil only, the second-stage juveniles or males were used instead. Molecular characterization was performed by PCR using species-specific primers and DNA sequencing on the ribosomal DNA 18S, ITS and 28S D2/D3, intergeneric spacer, RNA polymerase II large subunit, and mitochondrial DNA cytochrome oxidase gene subunit II. From these samples, *Meloidogyne arenaria, M. enterolobii, M. graminis, M. hapla, M. incognita, M. javanica, M. marylandi* and *M. naasi* were identified. *M. enterolobii* was first detected in 2011 and quickly became an emerging RKN species. *M. enterolobii* has been confirmed in limited fields in Columbus, Craven, Cumberland, Duplin, Edgecombe, Greene, Harnett, Johnston, Jones, Lenoir, Nash, New Hanover, Pitt, Sampson, Wake, Wayne, and Wilson counties as of July 1, 2025. *Meloidogyne enterolobii* is believed to be an introduced species in North Carolina. This species is a major concern from sweetpotato growers in North Carolina and the southeastern USA because it affects both yield and quality of sweetpotato. To date, there are no commercially acceptable resistant cultivars available against this species, and thus *M. enterolobii* poses a significant threat to crop production in the region.

## The biogeography of free-living soil nematodes of the Atlantic region of Canada

Young, Erika H.^1,2^, C. Goyer^3^, L-P Comeau^3^, L. Jewell^4^, A. Unc^5,6^

^1^Atlantic Forestry Centre, Canadian Forest Service, Natural Resources Canada, Corner Brook, NL, Canada (erika.young@nrcan-rncan.gc.ca)

^2^Environmental Science program, Memorial University of Newfoundland, St. John’s, NL, Canada

^3^Fredericton Research and Development Centre, Agriculture and Agri-Food Canada, Fredericton, NB, Canada

^4^St. John’s Research and Development Centre, Agriculture and Agri-Food Canada, St. John’s, NL, Canada

^5^School of Science and the Environment, Grenfell Campus, Memorial University of Newfoundland, Corner Brook, NL, Canada

^6^Natural Resource Science, McGill University, Sainte-Anne-de-Bellevue, QC, Canada

### Abstract

The transformation of carbon and energy to and from most nodes of the food-web are essential functions that free-living nematodes provide to soil systems. These essential soil food-web functions underpin the goods and services that humans derive from soils including the storage of carbon and the production of food and fibre. Free-living nematode communities may be used as indicators of functional soil quality due to their inherent ability to reflect relative carbon and nutrient dynamics. Despite this, comprehensive nematode surveys are rare in Canada and the national distribution of nematode taxa remains poorly understood. An extensive soil survey was conducted in the Atlantic region of Canada from 2019 to 2021. The nematode communities of 248 samples from New Brunswick (NB), Nova Scotia (NS), Prince Edward Island (PEI), and Newfoundland and Labrador (NL) were extracted for DNA and the V7-V8 region of the 18S rDNA was sequenced. The effects of land use (crop agriculture, pasture, forest, wetland), geographic distance, and soil properties on the free-living nematode taxa and diversities were evaluated. The study showed that nematode community compositions were significantly different between the provinces and land uses. Shannon-Wiener diversity was significantly higher in crop agriculture than forest (2.64 vs 2.44 respectively) possibly due to the low biodiversity in the dominant acidic forest soils of the study area. All measured soil properties explained more variance in the nematode community compositions of soils under crop agriculture and pasture management than of forests. However, in the absence of land use, individual soil properties had no significant relationships with individual genera. Geographic distance was a good descriptor of the beta diversity of the soil nematode communities for managed systems but not for forests. It is likely that the biogeographical patterns of nematodes in managed systems were related to the differences in management across the Atlantic region of Canada rather than absolute spatial separation.

## Secretory biology and anthelmintic targets in human vector-borne nematodes

Zamanian, Mostafa

Department of Pathobiological Sciences, University of Wisconsin-Madison

### Abstract

Soil and vector-transmitted parasitic nematodes infect over one billion people and are a major cause of global morbidity. Parasite control in both human and animal medicine is suboptimal and threatened by the growing prospects of anthelmintic resistance. Motivated by the need for new treatments and curiosity about basic parasite biology, I will present recent work on how mosquito-transmitted nematodes navigate host tissues and manipulate their host environments to survive. I outline how cell-specific methods can be paired with high-content phenotyping approaches to help resolve the sensory and secretory determinants of host compatibility. These projects leverage related techniques to answer fundamental questions about molecular communication and the druggability of essential parasite behaviors at the host interface.

## Nematode biodiversity in Hong Kong and Southern China

Zhongying, Zhao, T. Wen, W. Bai, P. Shen, M. Ng, and K. Kwok

Department of Biology, Hong Kong Baptist University, Hong Kong SAR

### Abstract

The phylum Nematoda represents one of the most abundant and diverse groups of metazoans on Earth. Recent advances in sequencing technologies have enabled cost-effective DNA barcoding and classification of wild-isolated nematodes, significantly enhancing our understanding of their biodiversity and evolutionary history. In this study, we collected soil nematode samples from approximately 80 locations, primarily in Hong Kong and Southern China. Our analysis identified 30 distinct nematode species across various clades, including Rhabditomorpha and Diplogasteromorpha in Clade V, Cephalobomorpha, Panagrolaimomorpha, and Tylenchomorpha in Clade IV, as well as Monhysterida in Clade C. While most identified nematodes appear to be free-living, members of the Alloionematidae and Aphelenchoididae families are predicted to be parasitic based on their close relatives. Notably, eight of the species are likely to be new nematode species, exhibiting only modest rDNA sequence similarity to those in existing databases. Our taxonomic classification is grounded in ribosomal DNA sequencing, particularly the internal transcribed spacer (ITS) regions, utilizing either Sanger DNA sequencing or Oxford Nanopore Technologies. Species of representative phylogenetic groups were morphologically characterized using differential interference contrast (DIC) microscopy, scanning electron microscopy (SEM), or a combination of both. The isolated species and strains offer a snapshot of nematode biodiversity in these regions, reflecting soil health and serving as a foundation for ecological monitoring. Furthermore, these cultivated species and strains provide a valuable resource for advancing studies in nematode phylogenomics and population genetics within the scientific community.

